# Mollusc species from the Pontocaspian region – an expert opinion list

**DOI:** 10.3897/zookeys.827.31365

**Published:** 2019-03-05

**Authors:** Frank P. Wesselingh, Thomas A. Neubauer, Vitaliy V. Anistratenko, Maxim V. Vinarski, Tamara Yanina, Jan Johan ter Poorten, Pavel Kijashko, Christian Albrecht, Olga Yu. Anistratenko, Anouk D’Hont, Pavel Frolov, Alberto Martínez ándara, Arjan Gittenberger, Aleksandre Gogaladze, Mikhail Karpinsky, Matteo Lattuada, Luis Popa, Arthur F. Sands, Sabrina van de V lde, Justine Vandendorpe, Thomas Wilke

**Affiliations:** 1 Naturalis Biodiversity Center, P.O. Box 9517, 2300 RA Leiden, The Netherlands; 2 Department of Animal Ecology and Systematics, Justus Liebig University, Heinrich-Buff-Ring 26–32 IFZ, 35392 Giessen, Germany; 3 Department of Invertebrate Fauna and Systematics, Schmalhausen Institute of Zoology, National Academy of Sciences of Ukraine, B. Khmelnytsky Str. 15, 01030 Kiev, Ukraine; 4 Laboratory of Macroecology and Biogeography of Invertebrates, Saint-Petersburg State University, 7/9 Universitetskaya Naberezhnaia, 199034 Saint Petersburg, Russia; 5 Omsk State Pedagogical University, Tukhachevskogo Emb. 14, 644099 Omsk, Russia; 6 Moscow State University, Faculty of Geography, Leninskie Gory 1, 119991 Moscow, Russia; 7 Department of Zoology (Invertebrates), Field Museum of Natural History, 1400 S. Lake Shore Drive, Chicago, IL 60605–2496, USA; 8 Zoological Institute, Russian Academy of Sciences, Universitetskaya nab. 1, 199034 St. Petersburg, Russia; 9 Department of Cainozoic Deposits, Institute of Geological Sciences, National Academy of Sciences of Ukraine, O. Gontchar Str. 55b, 01054 Kiev, Ukraine; 10 Gittenberger Marine Research, Inventory & Strategy (GiMaRIS), BioScience Park Leiden, J.H. Oortweg 21, 2333 CH Leiden, The Netherlands; 11 Grigore Antipa National Museum of Natural History, Sos. Kiseleff Nr. 1, 011341 Bucharest, Romania; 12 Russian Federal Research Institute of Fisheries and Oceanography, V. Krasnoselskaya 17, 107140 Moscow, Russia

**Keywords:** Aral Sea, bivalves, Black Sea, Caspian Sea, conservation, gastropods, nomenclature, taxonomy

## Abstract

Defining and recording the loss of species diversity is a daunting task, especially if identities of species under threat are not fully resolved. An example is the Pontocaspian biota. The mostly endemic invertebrate faunas that evolved in the Black Sea – Caspian Sea – Aral Sea region and live under variable salinity conditions are undergoing strong change, yet within several groups species boundaries are not well established. Collection efforts in the past decade have failed to produce living material of various species groups whose taxonomic status is unclear. This lack of data precludes an integrated taxonomic assessment to clarify species identities and estimate species richness of Pontocaspian biota combining morphological, ecological, genetic, and distribution data. In this paper, we present an expert-working list of Pontocaspian and invasive mollusc species associated to Pontocaspian habitats. This list is based on published and unpublished data on morphology, ecology, anatomy, and molecular biology. It allows us to (1) document Pontocaspian mollusc species, (2) make species richness estimates, and (3) identify and discuss taxonomic uncertainties. The endemic Pontocaspian mollusc species richness is estimated between 55 and 99 species, but there are several groups that may harbour cryptic species. Even though the conservation status of most of the species is not assessed or data deficient, our observations point to deterioration for many of the Pontocaspian species.

## Introduction

**Figure 1.**
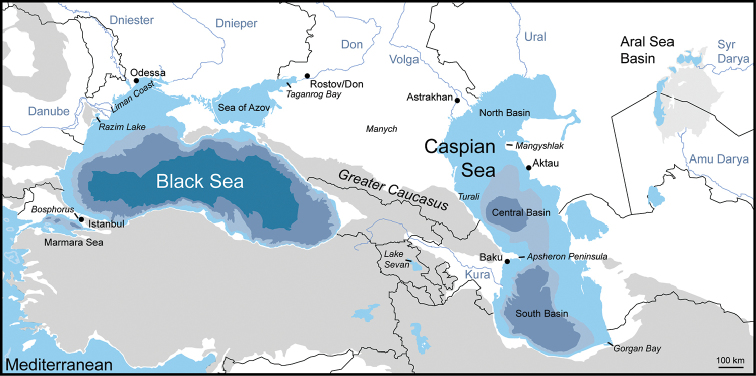
Map of the Pontocaspian region with the indication of major basins, rivers, regions, and cities referred to in the text.

The aquatic Pontocaspian (or Ponto-Caspian) biota is constituted by taxa that evolved in saline water bodies in the Caspian Sea – Black Sea – Aral Sea region and surrounding rivers in the past few million years. They include diverse groups such as diatoms, dinoflagellates, foraminiferans, crustaceans, molluscs, as well as fish and the Caspian seal. Major Pontocaspian habitats are located in the northern coastal zone of the Black Sea (mostly confined to the Romanian and Ukrainian coasts) and the Sea of Azov (mostly in the Taganrog Bay), cover the entire Caspian Sea and, until recently, the Aral Sea (Fig. 1). However, Pontocaspian habitats are impacted by human activities such as pollution, habitat modification and introduction of invasive species (Bologa et al. 1995, Zolotarev 1996, Zaitsev and Mamaev 1997, Gomoiu et al. 2002, Grigorovich et al. 2003, Occhipinti-Ambrogi and Savini 2003, Barannik et al. 2004, Shalovenkov 2005, UNEP 2006, Stolberg et al. 2006, Selifonova 2008a, b, Popa et al. 2009), as well as the entire obliteration of environments in the case of the Aral Sea in the second half of the 20^th^ century (Mainguet and Létolle 1997, Andreeva and Andreev 2003, Plotnikov et al. 2016).

Faunas in the Pontocaspian region have strongly changed in the past century. Pontocaspian species that were abundant only a century ago, such as *Dreissenaelata* and *D.caspia* in the Caspian Sea, have vanished in the mid-20^th^ century (Kosarev and Yablonskaya 1994). For the Aral Sea, the faunas appear to have largely disappeared with the demise of the lake system since the 1950s (Andreeva and Andreev 2003). Abundances of several other species in the Caspian Sea and Black Sea Basin have severely declined (Bologa et al. 1995, Zaitsev and Mamaev 1997, Barannik et al. 2004, Popa et al. 2012).

However, we cannot evaluate the extent or nature of biodiversity loss as there is no general agreement on the species that it might concern. Much of the diversity in Pontocaspian mollusc groups is contained within a limited number of genera. Changing taxonomic approaches through time (e.g., Zhadin 1952, Logvinenko and Starobogatov 1969, Alexenko and Starobogatov 1987, Sitnikova and Starobogatov 1999, Munasypova-Motyash 2006a, b, Anistratenko 2007b, Kijashko in Bogutskaya et al. 2013, Vinarski and Kantor 2016, Neubauer et al. 2018) combined with large morphological variability and few diagnostic characters in certain groups, as well as the paucity of living material and partial disappearance of type material, has precluded critical reassessment of species boundaries and thus species richness. For the Caspian Sea, multiple efforts to collect fresh material in the past decade failed to produce sufficient living material to elucidate these taxonomic matters for most of the groups. Sampling efforts include coastal sampling around Turali, Russia (FW, 2003); northern Azerbaijan (FW, 2016), middle and southern Azerbaijan (VA, ML, AFS, TW, 2017); Mangyshlak region coastal areas, Kazakhstan (OA, VA, 2016, 2017); the transition of the northern to middle Caspian Sea Basin in Kazakhstan (PRIDE expedition, 2017); and the Gorgan Bay in Iran (AFS, 2018). A faunal inventory of the deep-water southern Caspian Sea Basin (> 200 m water depth) of southern Azerbaijan was published lately by Mirzoev and Alekperov (2017). We are uncertain whether it concerns living material nor can we assess the species identities. Their records are mentioned below but require further confirmation. We did find some living endemic species ourselves, and from coastal areas low numbers of such species have been reported elsewhere (e.g., Latypov 2015). Yet, many species and even groups of species (e.g., *Turricaspia* species) have not been encountered alive despite our attempts. Our inability to collect life specimens for several groups has made a combined molecular-morphological approach to delineate species impossible. As a result, a reliable estimate of the number of species involved is lacking, and therefore the potential magnitude of the biodiversity decline is speculative. Hence, we need an alternative approach to outline the species boundaries and estimate the numbers affected.

By pooling all insights, data (published and unpublished) and expert opinions on the Pontocaspian mollusc species through taxonomists we aim to provide a list of Pontocaspian mollusc species that can serve as a base for further research. We use molluscs as a model group since they are (1) an important, representative and well-known part of the Pontocaspian fauna, (2) have a number of taxonomic specialists available, and (3) can often be identified based on their shell characters even when living populations have vanished. The Pontocaspian aquatic mollusc species list will highlight uncertainties in species complexes as to give guidance to further research in resolving taxonomic matters. The aim of this work is to compile a list of Pontocaspian mollusc species with the underlying arguments why we consider these species as (likely) valid species, to outline taxonomic uncertainties and to provide an updated estimate of species richness.

## Materials and methods

A preliminary Pontocaspian mollusc species list was assembled during a PRIDE program workshop in Giessen, Germany, in May 2018. The PRIDE (“Drivers of Pontocaspian Rise and biodiversity Demise”) program is an EU funded Innovative Training Network that studies the drivers of the rise and demise of Pontocaspian faunas. Using listings in Vinarski and Kantor (2016) supplemented with further information from the participants, this initial list was then circulated among a wider community of taxonomic workers for further updates and comments. Data on distribution and type material were derived from Vinarski and Kantor (2016) and further completed and amended. The systematic order above the species level follows Bouchet et al. (2017) and MolluscaBase (2018a). In cases where we deviate from the supraspecific classification, arguments are discussed below.

The list comprises aquatic Holocene Pontocaspian mollusc faunas. A substantial number of Pontocaspian species has been described from empty shells from beach material or derive from grab samples. Such samples typically are dominated by time-averaged Holocene shell assemblages, which may or may not yield living specimens and in very rare occasions also contain older (Pleistocene) material (see, e.g., Leroy et al. 2018). For the Black Sea Basin, the Holocene time interval largely coincides with the date of the marine flooding through the Bosphorus and subsequent marginalisation of Pontocaspian species to the NW coastal zone (Danube Delta to Dnieper Estuary) and the Sea of Azov (Mordukhay-Boltovskoy 1960). For the Caspian Sea, the time interval corresponds to the so-called Novocaspian period that started after the very deep Mangyshlak regression 8 ka (Fedorov 1953, Nevesskaja 1958, 2007, Yanina 2005). The time interval contains the earliest impact of humans on native faunas, such as the introduction of *Cerastodermaglaucum* in the Caspian Basin during the early Holocene (Fedorov 1957, Yanina 2009). It also contains the large faunal changes of the 20^th^ century related to pollution, invasive species, and obliteration of habitats (Kosarev and Yablonskaya 1994).

One of the greatest difficulties is to establish the identities of taxa reported as geographic subspecies. Many species have forms, varieties, and subspecies described from the Aral Sea, the Caspian Sea Basin, and the Black Sea Basin (including the Azov Sea). Often, such distinctions are made based on the geographical isolation alone or on a range of morphological characters whose variation seems to be overlapping in geographical subpopulations. In order to assess whether the geographical populations are indeed species, we need combined morphological, ecological, and molecular data, but only few studies produced this information to date (e.g., Popa et al. 2012 for Black Sea Basin *Monodacna*). For the Aral Sea, we expect difficulties to obtain fresh material of almost all species for molecular analyses due to the obliteration of most of the lake and its fauna in the 20^th^ century (Andreeva and Andreev 2003, Plotnikov et al. 2016). To date, hardly any molecular data on closely related species that are (potentially) shared between the Caspian and Black Sea have been published with the exceptions of *Dreissenagrimmi/D.bugensis* (e.g., Therriault et al. 2004, Stepien et al. 2013) and *Ecrobiamaritima/E.grimmi* (Haase et al. 2010). For several potentially shared species, ecological tolerances and preferences between Caspian and Black Sea Basin populations are overlapping, but in some cases (like for *D.grimmi*/*D.bugensis*) they are not. We have adopted a conservative approach, and as long as no additional arguments (morphological, ecological, or genetic differences) were found, we consider the Aral, Caspian and Black Sea varieties/subspecies synonyms. Another difficulty in especially Caspian taxa is the erection of so-called “bathymetric” subspecies, which seem to be distinguished mostly based on their depths of occurrence. As long as no other (morphological, genetic) arguments are available, we synonymise such bathymetrical forms.

A listing of synonyms and important past misidentifications from the literature is given. The list is not exhaustive and intended to show major shifts in taxonomic thinking about Pontocaspian and invasive species. The format of synonymy lists follows mostly suggestions of Matthews (1973). Asterisks in front of a record indicate valid first descriptions, a superscript “o” a prior yet invalidly introduced synonym. The status of each species is defined according to criteria outlined in Table 1.

**Table 1.** Definitions we use to characterise the status of species.

Pontocaspian	Centre of evolutionary history in Pontocaspian lakes
Native	Present in the Pontocaspian region today and in the Quaternary (not introduced by man) but centre of evolution not necessarily in that region: e.g., planorbid species with a Palearctic distribution, *Cerastodermaglaucum*.
Introduced	Species introduced in the Pontocaspian from elsewhere, usually anthropogenic: some Pontocaspian species have migrated between Pontocaspian basins and their status is explained in detail there (e.g., *Monodacnacolorata/Dreissenabugensis*: introduced in Caspian from natural ranges in Black Sea Basin).
Invasive	Species that have become disruptive in the ecosystem after introduction.

## Systematic catalogue

### Bivalvia

**Remarks.** Within the endemic bivalve species groups, a general lack of combined molecular, morphological, and ecological approaches has led to partially unresolved taxonomy, especially within the genera *Monodacna* and *Dreissena*. Much of the bivalve taxonomy follows the latest review of Caspian bivalves by Kijashko in Bogutskaya et al. (2013), and we discuss deviations from his schedule. The list of Aral bivalves published by Vinarski and Kantor (2016) is based chiefly on Andreeva and Andreev (2003), and it is used here as a base with appropriate changes in nomenclature.

#### Family Mytilidae Rafinesque, 1815

***Mytilasterminimus* (Poli, 1795)**

*1795 *Mytilusminimus* Poli: 209–210, pl. 32, fig. 1.

1932 *Mytilasterlineatus* (Gmelin, 1790). – Bogachev: 38, pl. 1, figs 5–11 [**non***Mytiluslineatus* Gmelin, 1791].

1952 *Mytilasterlineatus* (Gmelin, 1789). – Zhadin: 285, fig. 248 [**non** Gmelin, 1791].

1969 *Mytilasterlineatus* (Gmel.). – Logvinenko and Starobogatov: 311–312, figs 339a, b, pl. 5, figs 1, 2 [**non** Gmelin, 1791].

1969 *Mytilasterlineatus* (Gmelin, 1790). – Vekilov: 155–157, pl. 35, figs 1–25 [**non** Gmelin, 1791].

2013 *Mytilasterlineatus* (Gmelin, 1791). – Kijashko in Bogutskaya et al.: 316, fig. 104 [**non** Gmelin, 1791].

**Status.** Native to Black Sea Basin, invasive in Caspian Sea, introduced in Aral Sea but extinct there.

**Type locality.** Sicily, Italy.

**Distribution.** Native to the Mediterranean and Black Sea. Introduced in the Caspian Sea between 1917 and 1919 (Grigorovich et al. 2003).

**Taxonomic notes.** This species has commonly been mentioned as *Mytilasterlineatus* (Gmelin, 1791), but the Caspian-Aral species lacks the ribbing typical for that species. The attribution to *M.minimus* is based on shell morphology but confirmation from molecular analyses is required.

**Remarks.***Mytilasterminimus* has successfully replaced *Dreissenacaspia* and *D.elata* between 1938 and 1957 (Kostianoy and Kosarev 2005) in the Caspian Sea. Logvinenko and Starobogatov (1969) reported this species from the southern areas of the northern Caspian Sea, in the middle and the southern Caspian Sea down to 35–50 m water depth. Rarely, small individuals were found at depths down to 100 m. The species does not tolerate salinities below 7–8‰. This species was mentioned from depths between 200 and 600 m in the South Caspian Basin of Azerbaijan (Mirzoev and Alekperov 2017, who reported the species as *M.lineatus*). These deep records are unusual given other records and will require confirmation.

**Conservation status.** Not assessed.

#### Family Cardiidae Lamarck, 1809

**Remarks.** For the genus *Cerastoderma*, the species status of Pontocaspian material is subject of debate where morphological and increasingly molecular arguments show the possibility of sibling species occurrences (Sromek et al. 2016). The genus *Didacna* is relatively well established, however much uncertainty exists over distinction between the genera *Monodacna*, *Adacna*, and *Hypanis*. The generic concepts have shifted through time. Only lately, Kijashko in Bogutskaya et al. (2013) treated *Monodacna* as a subgenus of *Adacna*. Büyükmeriç and Wesselingh (2018) discussed the distinction between the three genera and considered *Monodacna*, *Adacna*, and *Hypanis* as valid.

***Adacnalaeviuscula* (Eichwald, 1829)**

*1829 *G.* [*lycymeris*] *laeviuscula* Eichwald: 279, pl. 5, fig. 1a, b.

1838 *AdacnaLaeviuscula* m. – Eichwald: 170–171.

1841 *Adacnalaeviuscula*. – Eichwald: 281–282, pl. 39, fig. 1a–d.

1905 *Adacnalaeviuscula* (Eichwald, 1829). – Ostroumov: pl. 2, fig. E.

1907 *Adacnalaeviuscula*. – Ostroumov: 25, text fig., pl. 4, figs 6–8.

1952 Adacna (Adacna) laeviuscula (Eichwald, 1829). – Zhadin: 353–354, pl. 9, fig. 331.

1958 Adacna (Adacna) laeviuscula (Eichwald), 1829. – Nevesskaja: 49–50, pl. 9, figs 15–18.

1969 *Hypanislaeviusculalaeviuscula* (Eichw.). – Logvinenko and Starobogatov: 337, fig. 353(5).

1973 *Hypanislaeviusculalaeviuscula* Eichwald, 1829. – Grossu: 144–145, text fig. 29.

2013 *Adacnalaeviuscula* (Eichwald, 1829). – Kijashko in Bogutskaya et al.: 377, fig. 154, photo 48.

2016 Adacna (Adacna) laeviuscula (Eichwald, 1829). – Vinarski and Kantor: 64.

**Status.** Pontocaspian species, endemic to Caspian Sea and possibly Black Sea Basin.

**Type locality.** Azerbaijan, Caspian Sea, Gulf of Baku is the type locality given by Vinarski and Kantor (2016) and this is written on the label of the type material. However, the type description reads “Hab. australem ripam maris caspii, in sinu Astrabadensi” [southern border of the Caspian Sea, in bight of Astrabad (= Gorgan, Iran)]. Further research on the labels and documentation is required to assess whether a new lectotype or even neotype must be assigned for *Adacnalaeviuscula*.

**Distribution.** Caspian Sea; limans, coastal lakes, and Danube Delta in Black Sea Basin (in case *A.fragilis* will be shown to be a synonym of *A.laeviuscula*).

**Taxonomic notes.** See discussion under *A.fragilis*.

**Remarks.**Kijashko in Bogutskaya et al. (2013) list the presence of this species at 30–60 m water depth in the Caspian Sea from muddy, sandy-mud, and rarely sandy bottoms. Logvinenko and Starobogatov (1969) reported the species from the northern, middle, and southern Caspian Sea basins down to 80–85 m water depth. In the Caspian Sea, the species has not been found in areas with salinities below 4‰. However, the common occurrence of fresh (paired) specimens on beaches seen at Turali (Dagestan, Russia) and northern Azerbaijan indicates this species maintains viable populations in foreshore and possibly even shoreface habitats.

**Conservation status.** Not assessed.

***Adacnafragilis* Milaschewitsch, 1908**

*1908 *Adacnafragilis* Milaschewitch: 992–993.

1973 *Hypanislaeviusculafragilis* Milaschevitsch, 1916. – Grossu: 145.

?2006b Hypanis (Adacna) laeviusculafragilis (Milachevitch, 1908). – Munasypova-Motyash: 522.

2009 Adacna (Adacna) fragilis Milaschevich, 1908. – Popa et al.: 13, fig. 5.

2016 Adacna (Adacna) fragilis Milaschewitsch, 1908. – Vinarski and Kantor: 64.

**Status.** Pontocaspian species, Black Sea Basin, status uncertain.

**Type locality.** Odessa region, Dniester liman and Katlabhuk Lake (Ukraine: Vinarski and Kantor 2016).

**Distribution.** Danube Delta region and NW Black Sea Basin coastal areas of Ukraine.

**Taxonomic notes.** We are uncertain about the status of *Adacnafragilis* Milaschewitch, 1908. The Black Sea Basin material has a wide variety of shapes and often is thinner and sometimes more elliptical than the Caspian *A.laeviuscula*. Both forms were synonymised by Graf and Cummings (2018) and indicated as a possible synonym in MolluscaBase (2018b). However, the Black Sea Basin occurrences are recorded from (coastal) lakes and small rivers suggesting little or only partial overlap in the ecological (and especially salinity) preferences of *A.laeviuscula* (e.g., Munasypova-Motyash 2006a, b, Popa et al. 2009). We are uncertain if *A.fragilis* might constitute a geographical subspecies (a status advocated by Grossu 1973), and further molecular analyses are needed to clarify the status of the Black Sea taxon.

**Remarks.** The species has been reported alive by Popa et al. (2009) from the Razim Lake complex on the Romanian Black Sea coast.

**Conservation status.** Not assessed.

***Adacnaminima* Ostroumov, 1907**

*1907 *Adacnaminima* Ostroumov: 23, text fig., pl. 4, figs 1–5.

1952 Adacna (Adacna) vitreavar.minima (Ostroumoff, 1907). – Zhadin: 353.

1967 *Hypanisminimaostroumovi* Logvinenko and Starobogatov: 233.

1969 *Hypanisminimaostroumovi* Logv. et Star. – Logvinenko and Starobogatov: 338, fig. 354(3).

1973 *Hypanisminimaostroumovi* Logvinenko et Starobogatov, 1968. – Grossu: 146, text fig. 31.

?1974 *Hypanisminimasidorovi* Starobogatov: 246, fig. 213.

2003 *Hypanisminimaminima* (Ostroumov, 1907). – Andreeva and Andreev: 88, fig. 5.1(3, 4).

?2009 *Hypania* [sic] *minima* (Ostroumoff, 1907). – Filippov and Riedel: 75, fig. 4s, t.

2013 *Adacnaminimaostroumovi* (Logvinenko et Starobogatov, 1967). – Kijashko in Bogutskaya et al.: 378, fig. 146.

2016 Adacna (Adacna) minimaminima (Ostroumov, 1907). – Vinarski and Kantor: 64.

2016 Adacna (Adacna) minimaostroumovi Logvinenko et Starobogatov, 1967. – Vinarski and Kantor: 64.

**Status.** Pontocaspian species, endemic to Caspian Sea and Aral Sea; likely disappeared from the latter.

**Type locality.** The northern Caspian Sea and the Aral Sea (Vinarski and Kantor 2016).

**Distribution.** Aral Sea (probably extinct there; Andreeva and Andreev 2003), Caspian Sea.

**Taxonomic notes.**Graf and Cummings (2018) consider this species as a synonym of *A.vitrea*, but Kijashko in Bogutskaya et al. (2013) regards it as a valid species. The latter considers *A.minimaminima* from the Aral Sea and *A.minimaostroumovi* syn. n. from the Caspian Sea as distinct geographical subspecies. The likely disappearance of the species from the Aral Sea makes a molecular assessment of their distinctness very difficult and given the lack of other arguments we synonymise both. Furthermore, we are uncertain about the status of the subspecies *Hypanisminimasidorovi* Starobogatov, 1974 from the western Aral Sea. Without further data we assume it concerns a form that falls within the wide morphological variation of *A.minima*. We moreover are very uncertain as to the status of *Hypanisminima* from Holocene deposits of Aral Sea as illustrated by Filippov and Riedel (2009, fig. 4s, t). The juvenile specimen has relatively strong cardinal teeth, onset of clear ribs, and a general shape that more resembles *Monodacnacaspia*.

**Remarks.** The species has been recorded mostly from the middle and southern Caspian Sea and more rarely from the eastern areas in the northern Caspian Sea down to 35 m water depth (Logvinenko and Starobogatov 1969) as well as from the Aral Sea from where it may have disappeared.

**Conservation status.** Not assessed.

***Adacnavitrea* (Eichwald, 1829)**

*1829 *G.* [*lycymeris*] *vitrea* Eichwald: 279, pl. 5, fig. 3.

1838 *Adacnavitrea* m. – Eichwald: 172–173.

1841 *Adacnavitrea*. – Eichwald: 282–283, pl. 39, fig. 2a, b.

1905 *Adacnaglabra* Ostroumov: 18–19.

1932a *Adacnavitrea* (Eichwald, 1829). – Bogachev: pl. 1, figs 3, 4, 11.

1932b *Adacnavitrea* (Eichwald, 1829). – Bogachev: 33, pl. 3, figs 13–16, 28–29.

1952 Adacna (Adacna) vitrea (Eichwald, 1829). – Zhadin: 352–353, fig. 330.

1958 Adacna (Adacna) vitrea (Eichwald), 1838. – Nevesskaja: 47–48, pl. 9, figs 19–22.

1969 *Hypanisvitreavitrea* (Eichw.). – Logvinenko and Starobogatov: 337, fig. 354(1), pl. 5, fig. 11.

1969 *Hypanisvitreaglabra* (Ostr.). – Logvinenko and Starobogatov: 338, fig. 354(2).

1973 *Hypanisvitreavitrea* Eichwald, 1829. – Grossu: 145–146, text fig. 30A.

1973 *Hypanisvitreaglabra* Ostroumoff, 1905. – Grossu: 146, text fig. 30B.

2003 *Hypanisvitreabergi* Starobogatov, 1974. – Andreeva and Andreev: 86, fig. 5.1(1, 2).

2013 Adacna (Adacna) vitreavitrea (Eichwald, 1829). – Kijashko in Bogutskaya et al.: 378, fig. 148.

2013 Adacna (Adacna) vitreaglabra Ostroumoff, 1905. – Kijashko in Bogutskaya et al.: 379, fig. 149.

2016 Adacna (Adacna) vitreavitrea (Eichwald, 1829). – Vinarski and Kantor: 65.

2016 Adacna (Adacna) vitreaglabra Ostroumov, 1905. – Vinarski and Kantor: 65.

2016 Adacna (Adacna) vitreabergi (Starobogatov, 1974). – Vinarski and Kantor: 65.

**Status.** Pontocaspian species, endemic to Caspian Sea Basin, Black Sea Basin, and Aral Sea Basin.

**Type locality.** “Australem oram caspii maris, Astrabadensem” [southern coast of Caspian Sea, near Astrabad (= Gorgan, Iran)].

**Distribution.** Black Sea Basin (also in Azov Sea and adjacent lower Don River), Caspian Sea Basin, and Aral Sea (including delta of Amu-Darya River). The Aral populations may have gone extinct in the 1980s (Andreeva and Andreev 2003).

**Taxonomic notes.** The species has been subdivided into three geographical subspecies which were not recognised by Graf and Cummings (2018). It concerns a species with thin shells that yield very few diagnostic characters that show overlap. Here, we synonymise the subspecies pending molecular assessments of their status.

**Conservation status.** Not assessed.

***Cerastodermaglaucum* (Bruguière, 1789) s.l.**

*1789 *Cardiumglaucum* Bruguière: 221–222.

1789 *CardiumGlaucum* Poiret: 13–15.

1869 *Cardiumisthmicus* Issel: 74–76.

1952 *Cardiumedule* L., 1758. – Zhadin: 344–345, fig. 318 [**non***Cardiumedule* Linnaeus, 1758].

2003 *Cerastodermaisthmicum* (Issel, 1869). – Andreeva & Andreev: 54, 62, figs 6.1(b), 6.7.

2013 *Cerastodermaglaucum* (Poiret, 1789). – Kijashko in Bogutskaya et al.: 342, fig. 126, photo 39.

2016 *Cerastodermaglaucum* (Bruguière, 1789). – Vinarski and Kantor: 69.

2016 *Cerastodermaisthmicus* (Issel, 1869). – Vinarski and Kantor: 70.

**Status.** Native Pontocaspian species (Black Sea Basin), Holocene invasive in Caspian Sea and Aral Sea.

**Type locality.** French Mediterranean.

**Distribution.** NE Atlantic, Baltic Sea, Mediterranean, Black Sea Basin, Caspian Sea Basin, Aral Sea, isolated Saharan lakes (Plaziat 1991).

**Taxonomic notes.** DNA studies have shown a strong structuring between Atlantic–western Mediterranean, Ionian, and Aegean-Pontocaspian populations of *C.glaucum* (Nikula and Väinölä 2003, Sromek et al. 2016). According to Sromek et al. (2016: 515), the “strong genetic differentiation and the occurrence of private alleles may hint at the presence of cryptic species within *C.glaucum*”. For a discussion on the authority of *C.glaucum*, see Vinarski and Kantor (2016: 69–70).

**Remarks.** The arrival of *Cerastodermaglaucum* in the Caspian Sea circa 8000 years ago has been linked to human settlement expansion through the Manych corridor (Fedorov 1957, Yanina 2009). It would be among the earliest human-mediated dispersal events for invertebrate species known to date.

**Conservation status.** Not assessed.

***Cerastoderma* sp. A [non *C.rhomboides* (Lamarck, 1819)**]

1916 Cardiumedulevar.nuciformis Milaschewitch: 257–259, pl. 7, figs 7, 8 [**non***Cardiumnuciforme* d’Orbigny, 1850].

2003 *Cerastodermarhomboidesrhomboides* (Lamarck, 1819). – Andreeva and Andreev: 93, fig. 6.1(A) [**non***Cardiumrhomboides* Lamarck, 1819].

2013 *Cerastodermarhomboides* (Lamarck, 1819). – Kijashko in Bogutskaya et al.: 343, fig. 127, photo 40 [**non** Lamarck, 1819].

2016 *Cerastodermarhomboides* (Lamarck, 1819). – Vinarski and Kantor: 70 [**non** Lamarck, 1819].

**Status.** Native Pontocaspian species (Black Sea Basin), introduced to Caspian Sea and Aral Sea.

**Distribution.** Black Sea (including Sea of Azov), Caspian Sea, Aral Sea, Aegean.

**Figure 2.**
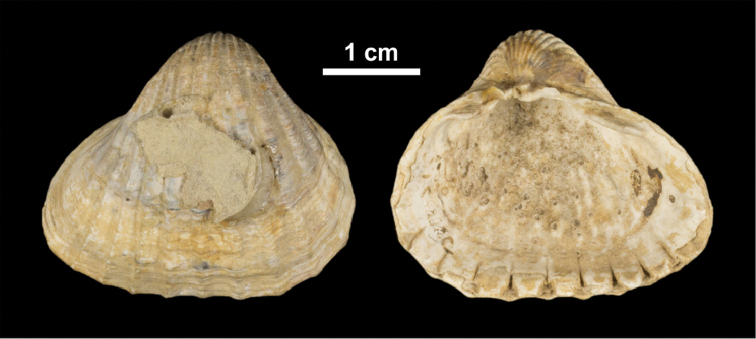
Syntype of *Cerastodermarhomboides* (Lamarck, 1819), stored in the Muséum national d’Histoire naturelle Paris (MNHN.F.A50142), Pliocene, Tuscany, Italy. Photograph by E Porez. https://science.mnhn.fr/institution/mnhn/collection/f/item/a50142?lang=fr_FR

**Taxonomic notes.** This concerns a common rhomboid-shaped species in the Pontocaspian region whose name is uncertain. It has a short ligament in common with *C.glaucum* and the persistent occurrence of ribs on the posterior margin, the well-defined character of the ribs and the regular occurrence of scales in common with western European *C.edule*. This form has been often referred to as *C.rhomboides* (Lamarck, 1819) that has been described from the Italian Pliocene but that concerns a typical *glaucum* form (Fig. 2), not the rhomboid form of the Pontocaspian *Cerastoderma*. The species has been named Cardiumedulevar.nuciformis by Milaschewitch (1916), but that name is a junior primary homonym of *Cardiumnuciforme* d’Orbigny, 1850. Even though some morphological features mentioned in the description of *C.lamarcki* (Reeve, 1845) may resemble those of the Pontocaspian species, the former has been traced to southern Great Britain from where molecular analyses only show the presence of *C.glaucum* (Nikula and Väinölä 2003).

**Conservation status.** Not assessed.

***Didacnabaeri* (Grimm, 1877)**

Fig. 3a

*1877 *CardiumBaeri* Grimm: 51–54, pl. 8, figs 2, 3.

1914 *DidacnaBaeri* (Grimm, 1877). – Nalivkin & Anisimov: 4, pl. 1, figs 4, 5.

1932 *DidacnaBaeri* (Grimm, 1877). – Bogachev: 29, pl. 3, figs 1–7.

1933 *DidacnaBaeri* (Grimm, 1877). – Zhizhchenko: 34, pl. 2, figs 5–8.

1952 *Didacnabaeri* (Grimm, 1877). – Zhadin: 347–348, figs 321, 322.

1953 *Didacnabaeri* (Grimm, 1877). – Fedorov: 129, pl. 20, figs 10, 11.

1968 *Didacnabaeri* (Grimm, 1877). – Gadzhiev: 76–77, pl. 1, figs 1, 2.

1969 *Didacnabaeri* (Grimm). – Logvinenko & Starobogatov: 324, fig. 344(2).

1969 *Didacnabaeri* (Grimm, 1877). – Vekilov: 139–144, pl. 25, figs 1–8.

1973 *Didacnabaeri* Grimm, 1877. – Grossu: 131, text fig. 7.

1983 *Didacnabaeri* (Grimm, 1877). – Popov: 180, pl. 16, figs 20–23.

1988 *Didacnabaeri* (Grimm, 1877). – Yanina & Svitoch: 129, pl. 3, figs 7–13.

2005 *Didacnabaeri* (Grimm, 1877). – Yanina: 242–244, pl. 14, figs 12–15.

2007 *Didacnabaeri* (Grimm, 1877). – Nevesskaja: 940–941, pl. 23, figs 11–17.

2013 *Didacnabaeri* (Grimm, 1877). – Kijashko in Bogutskaya et al.: 352, fig. 136, photo 41 [pars, excluding synonymy of *Didacnacrassa*].

2016 *Didacnabaeri* (Grimm, 1877). – Vinarski & Kantor: 71 [pars, excluding synonymy of *Didacnacrassa*].

**Status.** Pontocaspian species, endemic to Caspian Sea.

**Type locality.** Caspian Sea, offshore Turkmenistan, station 132, 40°32'N, 52°23'E.

**Distribution.**Logvinenko and Starobogatov (1969) reported *Didacnabaeri* from the southern basin (mostly on the eastern side) and from the middle basin down to 60 m water depth.

**Figure 3.**
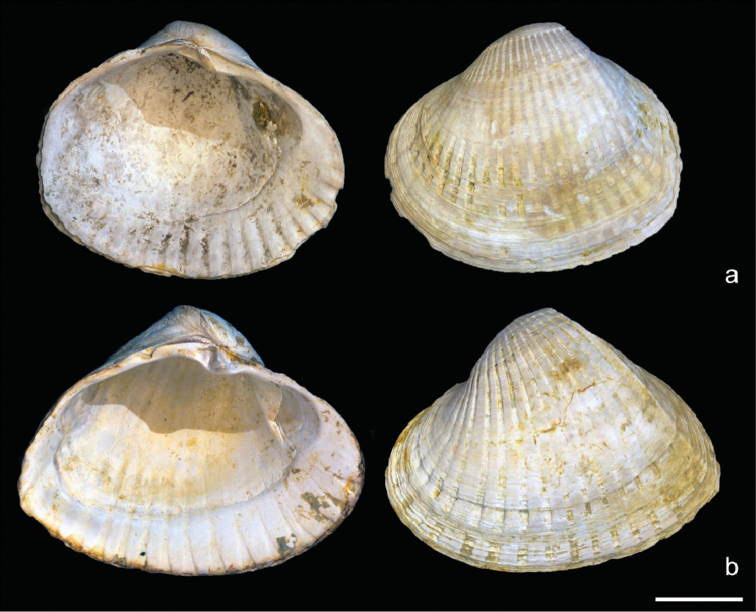
*Didacnabaeri* versus *D.eichwaldi* from Holocene (Novocaspian) deposits of Turali Lagoon (Dagestan, Russia). **a** RGM.961899, *Didacnabaeri* (Grimm, 1877) **b** RGM.961900, *Didacnaeichwaldi* (Krynicki, 1837), same locality. Scale bar: 1 cm.

**Taxonomic notes.** In recent works (e.g., Kijashko in Bogutskaya et al. 2013), the species *Didacnacrassa* (Eichwald, 1829) [= *D.eichwaldi* (Krynicki, 1837)] has been considered a synonym of *D.baeri*. However, both species can be distinguished. *Didacnabaeri* has a less extended, more roundish shell, a less developed keel, and a low top with less projecting beak and in general more ribs than *D.eichwaldi* (Fig. 3). *Didacnabaeri* occurred for the first time in the Novocaspian transgressive deposits whereas *D.crassa* already occurred in the late Khvalynian (Late Pleistocene). Both became very common during the Novocaspian.

**Conservation status.** Not assessed.

***Didacnabarbotdemarnii* (Grimm, 1877)**

*1877 *Cardium Barbot-de-Marnii* Grimm: 56–58, pl. 8, figs 5, 6.

1952 *Didacnabarbot-de-marnyi* [sic] (Grimm, 1877). – Zhadin: 348, fig. 323.

1969 *Didacnabarbotdemarnyi* [sic] (Grimm). – Logvinenko and Starobogatov: 326–327, fig. 346, pl. 5, fig. 8.

1973 *Didacnabarbotdemarnyi* [sic] Grimm, 1877. – Grossu: 133, text fig. 10.

2007 *Didacnabarbotdemarnyi* [sic] (Grimm, 1877). – Nevesskaja: 941–943, pl. 24, figs 10–14.

2013 *Didacnabarbotdemarnii* (Grimm, 1877). – Kijashko in Bogutskaya et al.: 353, fig. 139, photo 42.

2016 *Didacnabarbotdemarnii* (Grimm, 1877). – Vinarski and Kantor: 71.

**Status.** Pontocaspian species, endemic to Caspian Sea.

**Type locality.** Caspian Sea, station 116, 44°17'N, 50°22'E.

**Distribution.** Southern, middle, and southern part of the northern Caspian Sea down to 40 m water depth, preferentially on sandy sediments (Logvinenko and Starobogatov 1969).

**Conservation status.** Not assessed.

***Didacnaeichwaldi* (Krynicki, 1837)**

Fig. 3b

°1829 *C.* [*ardium*] *crassum* Eichwald: 283 [**non***Cardiumcrassum* Gmelin, 1791].

*1837 *CardiumEichwaldi* Krynicki: 61 [**nom. nov.** pro *C.crassum* Eichwald, 1829, **non** Gmelin, 1791].

1841 *Didacnacrassa*. – Eichwald: 273, pl. 39, fig. 6a, b.

1876 *Cardiumcrassum* Eichwald, 1829. – Grimm: 136–138, pl. 6, fig. 3.

1905 *Didacnacrassa* (Eichwald, 1829). – Ostroumov: 15, 69, pl. 2(A).

1932 Didacnaaff.crassa (Eichwald, 1829). – Bogachev: 27, pl. 2, figs 11–14.

1952 *Didacnacrassa* Eichwald, 1841. – Zhadin: 349, fig. 325.

1953 *Didacnacrassa* (Eichwald, 1829). – Fedorov: 130, pl. 20, figs 8, 9, 12, 13.

1958 *Didacnacrassacrassa* Eichwald, 1829. – Nevesskaja: 39–40, pl. 7, figs 8, 9.

1969 *Didacnacrassa* (Eichwald, 1829). – Vekilov: 134–139, pl. 24, figs 1–6, pl. 27, figs 1, 2.

1988 *Didacnacrassacrassa* (Eichwald, 1829). – Yanina and Svitoch: pl. 12, figs 8, 9, pl. 13, figs 1–5.

2005 *Didacnacrassa* (Eichwald, 1829). – Yanina: 242, pl. 14, figs 3–6.

2007 *Didacnacrassa* (Eichwald, 1829). – Nevesskaja: 939–940, pl. 23, figs 1–5.

2013 *Didacnabaeri* (Grimm, 1877). – Kijashko in Bogutskaya et al.: 352 [pars, non fig. 136, photo 41, **non***Cardiumbaeri* Grimm, 1877].

2016 *Didacnabaeri* (Grimm, 1877). – Vinarski and Kantor: 71 [pars, **non** Grimm, 1877].

**Status.** Pontocaspian species, endemic to Caspian Sea.

**Type locality.** “Caspium mare” (Caspian Sea) (for *C.crassum* Eichwald, 1829).

**Distribution.** Caspian Sea. *Didacnaeichwaldi* is known from the middle and southern Caspian Sea basins down to 35 m water depth and cannot tolerate lowered salinities.

**Taxonomic notes.** In recent works (Kijashko in Bogutskaya et al. 2013), the species *Didacnacrassa* (Eichwald, 1829) [= *D.eichwaldi* (Krynicki, 1837)] has been considered a synonym of *D.baeri*. However, we see morphological discontinuities in our extensive material from the northern Caspian Sea Basin that implies that *D.eichwaldi* with its protruding umbo and shouldered appearance is distinct from *D.baeri* that is characterised by a rounded umbo (see discussion above under *D.baeri*). Despite being in common usage, the name *Didacnacrassa* is invalid as it is a junior homonym of *Cardiumcrassum* Gmelin, 1791; Krynicki (1837) introduced *Cardiumeichwaldi* as replacement name.

**Conservation status.** Not assessed.

***Didacnalongipes* (Grimm, 1877)**

*1877 *Cardiumlongipes* Grimm: 54–56, pl. 8, fig. 4a–c.

1952 *Didacnalongipes* (Grimm, 1877). – Zhadin: 349–350, fig. 326.

1969 *Didacnalongipes* (Grimm). – Logvinenko and Starobogatov: 326, fig. 345.

1973 *Didacnalongipes* Grimm, 1877. – Grossu: 132, text fig. 9, pl. 1, fig. 2.

?2007 *Didacnacarinata* Nevesskaja: 943, pl. 24, figs 15–19.

2013 *Didacnalongipes* (Grimm, 1877). – Kijashko in Bogutskaya et al.: 354, fig. 137, photo 43.

2016 *Didacnalongipes* (Grimm, 1877). – Vinarski and Kantor: 71.

**Status.** Pontocaspian species, endemic to Caspian Sea.

**Figure 4.**
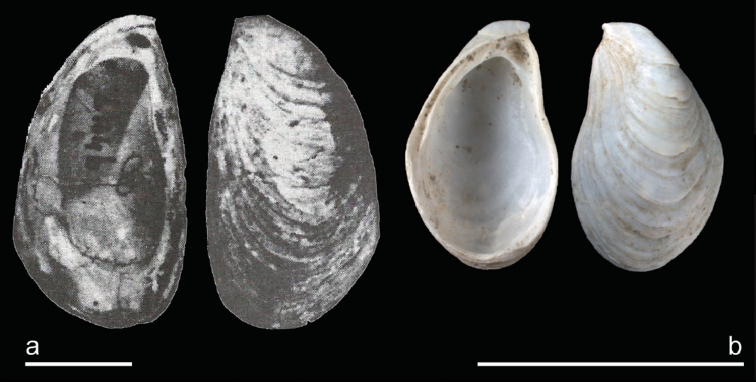
Lectotype *Dreissenarostriformis* versus *D.grimmi*. **a***D.rostriformis* Deshayes, 1838. Lectotype. Pliocene, Crimea. Reproduced from Archambault-Guezou (1976, pl. 6, fig 2a-2c) **b** RGM.961901, *D.grimmi* (Andrusov, 1890). Caspian Sea offshore Aktau, Kazakhstan, sample KAZ17-21, depth 44.3 m. Scale bar: 1 cm.

**Type locality.** Caspian Sea, offshore Azerbaijan, approximately 40°39'N, 50°26'E.

**Distribution.** Southern and middle Caspian Sea basins and southern part of the northern Caspian Sea down to 30–40 m water depth. The species often co-occurs with *D.barbotdemarnii*.

**Remarks.** We are uncertain about the status of *Didacnacarinata* Nevesskaja, 2007. The overall outline resembles that of *D.barbotdemarnii*, but the former species appears smaller and thinner. Kijashko in Bogutskaya et al. (2013) considered *D.carinata* as a synonym of *D.longipes*.

**Conservation status.** Not assessed.

***Didacnaparallela* Bogachev, 1932**

*1932a *Didacnaparallela* Bogachev: pl. 2, figs 2, 3.

1932b *Didacnaparallela* Bogachev: 44, pl. 5, figs 1–7, 9.

1953 *Didacnaparallella* [sic] Bogatchev, 1932. – Fedorov: 126, pl. 17, figs 1–11.

1969 *Didacnaparallella* [sic] Bog. – Logvinenko and Starobogatov: 324–325, fig. 344(3).

1969 *Didacnaparallella* [sic] Bogatchev, 1932. – Vekilov: 117–120, pl. 21, figs 1–8.

1973 *Didacnaparallella* [sic] Bogatchev, 1922 [sic]. – Grossu: 131, text fig. 8, pl. 1, fig. 4.

2005 *Didacnaparallella* [sic] Bogatchev, 1932. – Yanina: 237–238, pl. 12, figs 1–8.

2007 *Didacnaparallella* [sic] Bogatchev, 1932. – Nevesskaja: 933–935, pl. 21, figs 1–5.

2013 *Didacnaparallela* Bogachev, 1932. – Kijashko in Bogutskaya et al.: 355–356, fig. 138.

2016 *Didacnaparallela* Bogachev, 1932. – Vinarski and Kantor: 72.

**Status.** Pontocaspian species, endemic to Caspian Sea.

**Type locality.** Khala, Apsheron Peninsula, Azerbaijan (early Khvalynian, Late Pleistocene).

**Distribution.** Caspian Sea, southern basin and western part of middle basin between 50–85 m water depth (Logvinenko and Starobogatov 1969). This species was mentioned from depths between 200 and 300 m in the South Caspian Basin of Azerbaijan (Mirzoev and Alekperov 2017), but we are not certain whether it concerns living specimens.

**Remarks.***Didacnaparallela* has been considered as extinct by Nevesskaja (2007) but was nevertheless treated in Kijashko in Bogutskaya et al. (2013). Live records are known at least until 1986 and we have no particular reason to assume it is extinct.

**Conservation status.** Not assessed.

***Didacnapraetrigonoides* Nalivkin & Anisimov, 1914**

*1914 *Didacnapraetrigonoides* Nalivkin & Anisimov: 5–6, 16–17, pl. 1, figs 1, 2.

1932a *Didacnapraetrigonoides* Nalivkin & Anisimov, 1914. – Bogachev: pl. 2, fig. 1.

1932b *Didacnapraetrigonoides* Nalivkin & Anisimov, 1914. – Bogachev: 42, pl. 4, figs 1–8, pl. 5, fig. 8.

1948 *Didacnapraetrigonoides* Nal. – Fedorov: pl. 2, figs 10–13.

1953 *Didacnapraetrigonoides* Nalivkin & Anisimov, 1914. – Fedorov: 128, pl. 18, figs 1–6, pl. 19, figs 1–6.

1958 *Didacnapraetrigonoides* Nalivkin & Anisimov, 1914. – Nevesskaja: 17–20, pl. 1, figs 1–14.

1969 *Didacnatrigonoidespraetrigonoides* Nal. & Anis. – Logvinenko and Starobogatov: 324, fig. 343(2).

1969 *Didacnapraetrigonoides* Nalivkin & Anisimov, 1914. – Vekilov: 120–128, pl. 22, figs 1–9.

1973 *Didacnatrigonoidespraetrigonoides* Nalivkin & Anisimov, 1915. – Grossu: 129, text fig. 5.

1983 *Didacnapraetrigonoidespraetrigonoides* Nalivkin & Anisimov, 1914. – Popov: 195, pl. 15, figs 1, 2.

1988 *Didacnapraetrigonoides* Nalivkin & Anisimov, 1914. – Yanina and Svitoch: pl. 8, figs 4–7.

2005 *Didacnapraetrigonoides* Nalivkin & Anisimov, 1914. – Yanina: 241, pl. 14, figs 1, 2.

2007 *Didacnapraetrigonoidespraetrigonoides* Nalivkin & Anisimov, 1914. – Nevesskaja: 927, pl. 19, figs 9, 10.

**Status.** Pontocaspian species, endemic to Caspian Sea. Possibly extinct.

**Type locality.** Apsheron Peninsula, Azerbaijan, Quaternary.

**Distribution.** Caspian Sea. Logvinenko and Starobogatov (1969) reported the species from the southern Caspian Sea Basin and the southern part of the middle Caspian Sea Basin down to 60 m water depth. The species has been collected from Holocene deposits and beach occurrences the western part of the middle Caspian Sea Basin as well (FW, pers. obs.). The species is reportedly extinct, not mentioned in Kijashko in Bogutskaya et al. (2013).

**Remarks.** The first appearance of *Didacnapraetrigonoides* is in lower Khvalynian deposits, it became widespread during the late Khvalynian and was rare during the Novocaspian.

**Conservation status.** Not assessed. *Didacnapraetrigonoides* has been reported to occur ‘rarely in the modern Caspian Sea’ (Nevesskaja 2007: 927), but material from recent assemblages has not been found.

***Didacnaprofundicola* Logvinenko & Starobogatov, 1966**

*1966a *Didacnaprofundicola* Logvinenko & Starobogatov: 13–14, fig. 1.

1969 *Didacnaprofundicola* Logv. & Star. – Logvinenko and Starobogatov: 328–329, fig. 349.

1973 *Didacnaprofundicola* Logvinenko & Starobogatov, 1966. – Grossu: 134, text fig. 13.

2007 *Didacnaprofundicola* Logvinenko & Starobogatov, 1966. – Nevesskaja: 944, pl. 20, fig. 28a–c.

2013 *Didacnaprofundicola* Logvinenko & Starobogatov. – Kijashko in Bogutskaya et al.: 356, fig. 140, photo 45.

2016 *Didacnaprofundicola* Logvinenko & Starobogatov. – Vinarski and Kantor: 72.

**Status.** Pontocaspian species, endemic to Caspian Sea.

**Type locality.** Central part of the Caspian Sea, 39°38'N, 52°02'E(offshore Turkmenistan).

**Distribution.** Middle and southern basins of Caspian Sea between 75 and 409 m water depth (Logvinenko and Starobogatov 1969). This species was mentioned from depths between 200 and 600 m in the South Caspian Basin of Azerbaijan (Mirzoev and Alekperov 2017).

**Conservation status.** Not assessed.

***Didacnaprotracta* (Eichwald, 1841)**

*1841 *Adacnaprotracta* Eichwald: 280, pl. 40, figs 10, 11 [non figs 9, 10 as indicated in the text].

1877 *Cardiumcatillus* Eichw. – Grimm: 58, pl. 8, figs 7, 8 [**non***Monodacnacatillus* Eichwald, 1841].

1910 *Didacnaprotracta* (Eichwald, 1841). – Andrusov: 67, pl. 8, figs 22, 33, pl. 9, figs 1–9.

1952 *Didacnaprotracta* (Eichwald, 1841). – Zhadin: 348–349, fig. 324.

1953 *Didacnaprotracta* (Eichwald, 1829). – Fedorov: 127, pl. 14, figs 12–15, pl. 15, figs 1–16.

1967 *Didacnaprotracta* Eichwald, 1841. – Svitoch: 42–43, pl. 6, figs 6–9, pl. 7, figs 1, 2.

1969 *Didacnaprotractaprotracta* (Eichw.). – Logvinenko and Starobogatov: 327, fig. 347.

1973 *Didacnaprotractaprotracta* Eichwald, 1841. – Grossu: 133, text fig. 11.

1973 *Didacnaprotractasubmedia* Andrusov, 1911. – Grossu: 133–134, text fig. 12.

1999 *Didacnaprotracta* (Eichwald, 1829). – Fedorov: pl. 12, figs 4–7.

2005 *Didacnaprotracta* (Eichwald, 1829). – Yanina: 238–239, pl. 12, figs 9–19.

2007 *Didacnaprotractaprotracta* (Eichwald, 1829). – Nevesskaja: 938–939, pl. 22, figs 4–13.

2013 *Didacnaprotracta* (Eichwald, 1829). – Kijashko in Bogutskaya et al.: 356, fig. 141.

2013 *Didacnaprotractasubmedia* Andrusov, 1910. – Kijashko in Bogutskaya et al.: 356, fig. 142.

2016 *Didacnaprotracta* (Eichwald, 1841). – Vinarski and Kantor: 72.

**Status.** Pontocaspian species, endemic to Caspian Sea.

**Type locality.** The type series (?Recent, Caspian Sea) was reported as lost by Nevesskaja (2007) who introduced a neotype from the Elton Lake surroundings in the northern Caspian plains, Russia (early Khvalynian, Late Pleistocene).

**Distribution.** Middle and southern Caspian Sea basins; it is most common in the middle basin at 25–85 m water depth (Logvinenko and Starobogatov 1969).

**Taxonomic notes.** According to Logvinenko and Starobogatov (1969), two subspecies occur in the Caspian Sea at different depth ranges: *D.protractaprotracta* at 25–50 m and *D.protractasubmedia* Andrusov, 1910 at 50–85 m. The latter differs from *D.p.protracta* by the relative posterior location of the umbo that is furthermore subdued. Both forms of *Didacnaprotracta* are widespread in the Khvalynian deposits of the Caspian Sea and Manych depression. According to Kijashko in Bogutskaya et al. (2013) morphological differences characteristic for the subspecies of *Didacnaprotracta* are due to allometric growth. The mere difference in depth distribution, with overlapping depths and intermediate forms, does not provide any argument to maintain these subspecies. *Didacnaprotracta* is the type species of the subgenus Protodidacna Logvinenko & Starobogatov, 1966.

**Remarks.** The authorship attribution of this species to Eichwald (1829) as proposed by several authors was rejected in Vinarski and Kantor (2016). According to them, *Cardiumprotractum* Eichwald, 1829, described from the western Ukraine, probably refers to a different species.

**Conservation status.** Not assessed.

***Didacnapyramidata* (Grimm, 1877)**

*1877 *Cardiumpyramidatum* Grimm: 46–49, pl. 8, fig. 1a–d.

1932 *Didacnapyramidata* (Grimm, 1877). – Bogachev: 28–29, pl. 2, figs 15, 16.

1952 *Didacnapyramidata* (Grimm, 1877). – Zhadin: 347, fig. 320.

1969 *Didacnapyramidata* (Grimm). – Logvinenko and Starobogatov: 324, fig. 344(1).

1969 *Didacnapyramidata* (Grimm, 1877). – Vekilov: 144–147, pl. 26, figs 1–5.

1973 *Didacnapyramidata* Grimm, 1877. – Grossu: 130, text fig. 6, pl. 1, fig. 1.

2007 *Didacnapyramidata* (Grimm, 1877). – Nevesskaja: 940, pl. 23, figs 6–10.

2013 *Didacnapyramidata* (Grimm, 1877). – Kijashko in Bogutskaya et al.: 357, fig. 135, photo 47.

2016 *Didacnapyramidata* (Grimm, 1877). – Vinarski and Kantor: 73.

**Status.** Pontocaspian species, endemic to Caspian Sea.

**Type locality.** Caspian Sea, offshore Azerbaijan, 39°47'N, 49°59'30"E (Kijashko in Bogutskaya et al. 2013).

**Distribution.** Caspian Sea: southern basin and southern part of the middle basin at depths between 30–100 m (Logvinenko and Starobogatov 1969).

**Conservation status.** Not assessed.

***Didacnatrigonoides* (Pallas, 1771)**

*1771 *Cardiumtrigonoides* Pallas: 478.

1831 *Cardiumtrigonoides* (Pallas, 1771). – Eichwald: 282.

1838 *Didacnatrigonoides* n. – Eichwald: 166–167.

1841 *Didacnatrigonoides*. – Eichwald: 271–272, pl. 39, fig. 5a–c.

1876 *Cardiumtrigonoides*, Pall. – Grimm: 138–140, pl. 6, fig. 2.

1914 *Didacnatrigonoides* (Pallas, 1771). – Kalitskiy: pl. 3, figs 1, 2.

1914 *Didacnatrigonoides* (Pallas, 1771). – Nalivkin and Anisimov: 6, pl. 1, fig. 3.

1932a *Didacnatrigonoides* (Pallas, 1771). – Bogachev: pl. 1, figs 5, 6.

1932b *Didacnatrigonoides* (Pallas, 1771). – Bogachev: 25, pl. 2, figs 1–9.

1933 *Didacnatrigonoides* (Pallas, 1771). – Zhizhchenko: 35–36, pl. 2, figs 9, 10.

1950 *Didacnatrigonoides* (Pallas, 1771). – Pravoslavlev: 21–22, figs 1–4.

1952 *Didacnatrigonoides* (Pallas, 1771). – Zhadin: 346, fig. 319.

1953 *Didacnatrigonoides* (Pallas, 1771). – Fedorov: 129, pl. 20, figs 7–9.

1969 *Didacnatrigonoidestrigonoides* (Pall.). – Logvinenko and Starobogatov: 323, fig. 343(1), pl. 5, fig. 7.

1969 *Didacnatrigonoides* (Pallas, 1771). – Vekilov: 128–134, pl. 23, figs 1–9, pl. 27, fig. 6.

1973 *Didacnatrigonoidestrigonoides* Pallas, 1771. – Grossu: 129, text fig. 4, pl. 1, fig. 3.

1977 *Didacnatrigonoidestuzetae* Tadjalli-Pour: 97, pl. 1, fig. 3.

1983 *Didacnatrigonoides* (Pallas, 1771). – Popov: 204, pl. 16, fig. 19.

1986 *Didacnatrigonoides* (Pallas, 1771). – Yakhimovich et al.: 79, pl. 10, fig. 1.

1988 *Didacnatrigonoides* (Pallas, 1771). – Yanina and Svitoch: pl. 9, figs 7–12.

2005 *Didacnatrigonoides* (Pallas, 1771). – Yanina: 244–245, pl. 14, figs 7–11.

2007 *Didacnatrigonoides* (Pallas, 1771). – Nevesskaja: 941, pl. 24, figs 1–9.

2013 *Didacnatrigonoides* (Pallas, 1771). – Kijashko in Bogutskaya et al.: 358, fig. 134.

2016 *Didacnatrigonoides* (Pallas, 1771). – Vinarski and Kantor: 70.

**Status.** Pontocaspian species, endemic to Caspian Sea.

**Type locality.** Caspian Sea, a neotype has been designated based on a specimen from Chechen Island by Nevesskaja (2007, pl. 24, fig. 4).

**Distribution.** Caspian Sea, mostly eastern part of northern Caspian Sea Basin (Logvinenko and Starobogatov 1969). Furthermore found in living position in Novocaspian deposits near Turali, Dagestan (western part middle basin; FW).

**Remark.** Genetic data are available through Albrecht et al. (2014).

**Conservation status.** Not assessed.

***Hypanisplicata* (Eichwald, 1829)**

*1829 *G.* [*lycymeris*] *plicata* Eichwald: 279, pl. 5, fig. 2a, b.

1838 *Adacne* [sic] *plicata* m. – Eichwald: 171–172.

1916 *Adacnarelicta* Milaschewitch: 274–276, pl. 8, figs 10–13 [non figs 10–12 as indicated in the text].

1926 Adacnarelictavar.dolosmiana Borcea: 468–469, pl. 18, figs 156–158, pl. 21, fig. 2.

1952 Adacna (Hypanis) plicata (Eichwald, 1829). – Zhadin: 354–355, fig. 332.

1958 Adacna (Hypanis) plicata (Eichwald), 1829. – Nevesskaja: 50–51, pl. 9, figs 9–14.

1969 *Hypanisplicataplicata* (Eichw.). – Logvinenko and Starobogatov: 331–332, fig. 350.

1973 *Hypanisplicataplicata* Eichwald, 1829. – Grossu: 136, text fig. 14, pl. 1, fig. 5.

1973 *Hypanisplicatarelicta* Milaschevitsch, 1916. – Grossu: 136, text fig. 15, pl. 1, figs. 6, 20–23.

1973 *Hypanisdolosmaniana* [sic] Borcea, 1826. – Grossu: 136, text fig. 16, pl. 1, figs 16–19.

1977 *Hypanisplicatagolbargae* Tadjalli-Pour: 99, pl. 1, fig. 5.

2006a *Hypanisplicatarelicta* (Milachevitch, 1916). – Munasypova-Motyash: 45–46.

2009 Adacna (Hypanis) plicatarelicta Milaschevich, 1916. – Popa et al. 12, fig. 4.

2013 *Hypanisplicata* (Eichwald, 1829). – Kijashko in Bogutskaya et al.: 387, fig. 164, photo 56.

2016 *Hypanisplicataplicata* (Eichwald, 1829). – Vinarski and Kantor: 73.

2016 *Hypanisplicatarelicta* (Milaschewitsch, 1916). – Vinarski and Kantor: 74.

**Status.** Pontocaspian species, endemic to Caspian Sea Basin and Black Sea Basin.

**Type locality.** “Sinum Astrabadensem” [Caspian Sea near Astrabad (= Gorgan, Iran)].

**Distribution.** Caspian Sea, western liman coast Black Sea Basin.

**Taxonomic notes.** The Black Sea populations of *H.plicata* show a large range of morphological variation with elongated specimens that cannot be distinguished from Caspian *H.plicata* to severely stunted and irregularly shaped specimens that have been considered as a subspecies (*H.plicatarelicta*) or as distinct species (*H.dolosmiana*) (e.g., Munasypova-Motyash 2006a). These forms have intermediates indicating that the Black Sea Basin specimens are a single species that should be attributed to *H.plicata* even though the latter appear to have lived under lower salinities than their Caspian counterparts. Molecular studies are required to elucidate the status of the Black Sea Basin material.

**Conservation status.** Not assessed. Fresh shells (including paired specimens) have been found at several beaches around the Caspian Sea (Turali, Dagestan, Russia; Şuraabad, Azerbaijan; FW). The species has been reported alive from the Razim lake complex of the Romanian Black Sea coast by Popa et al. (2009).

***Monodacnaacuticosta* (Logvinenko & Starobogatov, 1967)**

*1967 *Hypanisacuticosta* Logvinenko & Starobogatov: 232.

1969 *Hypanisangusticostataacuticosta* Logvinenko & Starobogatov: 334, fig. 353(1).

1973 *Hypanisangusticostataacuticosta* Logvinenko et Starobogatov, 1967. – Grossu: 141, fig. 23.

2013 Adacna (Monodacna) acuticosta (Logvinenko & Starobogatov, 1967). – Kijashko in Bogutskaya et al.: 379, fig. 160, photo 50.

2016 Adacna (Monodacna) acuticosta (Logvinenko & Starobogatov, 1967). – Vinarski and Kantor: 66.

**Status.** Pontocaspian species, endemic to Caspian Sea.

**Type locality.** “Northern Caspian Sea on the central part of the slope” (Vinarski and Kantor 2016: 66), which likely refers to northern slope of the middle Caspian Sea Basin.

**Distribution.** Caspian Sea (middle Caspian Sea Basin).

**Conservation status.** Not assessed.

***Monodacnaalbida* (Logvinenko & Starobogatov, 1967)**

*1967 *Hypanisalbida* Logvinenko & Starobogatov: 232.

1969 *Hypanisalbida* Logv. & Star. – Logvinenko and Starobogatov: 336, fig. 353(3).

1973 *Hypanisalbida* Logvinenko & Starobogatov, 1967. – Grossu: 144, text fig. 28.

2013 Adacna (Monodacna) albida (Logvinenko & Starobogatov, 1967). – Kijashko in Bogutskaya et al.: 380, fig. 162, photo 51.

2016 Adacna (Monodacna) albida (Logvinenko & Starobogatov, 1967). – Vinarski and Kantor: 66.

**Status.** Pontocaspian species, endemic to Caspian Sea.

**Type locality.** “Western Caspian Sea southeastwards from Derbent” (Vinarski and Kantor 2016: 66).

**Distribution.** Caspian Sea (middle and southern Caspian Sea Basin). This species was mentioned from depths between 200 and 400 m in the South Caspian Basin of Azerbaijan (Mirzoev and Alekperov 2017, who reported the species as *Hypanisalbida*).

**Taxonomic notes.** This species is part of a group of Caspian *Monodacna* with relative flat and wedge-shaped shells with low and sometimes poorly defined ribs (*M.albida, M.polymorpha*). Like for the *Monodacnacaspia* group (see below), we are in need of studies to assess whether these taxa might form ecomorphs of a single species.

**Conservation status.** Not assessed.

***Monodacnacaspia* (Eichwald, 1829)**

*1829 *C.*[*orbula*] *caspia* Eichwald: 281, pl. 5, fig. 6a, b.

1841 *Monodacnacaspia*. – Eichwald: 274, pl. 39, fig. 4a–c.

1905 *Monodacnacaspia* (Eichwald, 1829). – Ostroumov: pl. 3, fig. C.

1932a *Monodacnacaspia* (Eichwald, 1829). – Bogachev: pl. 1, figs 10, 13.

1932b *Monodacnacaspia* (Eichwald, 1829). – Bogachev: 30, pl. 3, figs 21–27.

1952 Monodacnaedentula(Pallas, 1771)var.caspia Eichwald, 1841. – Zhadin: 350, fig. 327B.

1958 *Monodacnacaspia* (Eichwald), 1829. – Nevesskaja: 44–46, pl. 9, figs 1–8.

1963 *Monodacnacaspiacaspia* (Eichwald, 1829). – Nevesskaja: 66, pl. 8, figs 1–4.

1965 *Monodacnacaspiacaspia* (Eichwald). – Nevesskaja: 187–198, pl. 9, figs 6–15, 17–19, 23–26, 29.

1969 *Monodacnacaspia* (Eichwald, 1829). – Vekilov: 147–150, pl. 31, figs 9–11.

1973 *Hypaniscaspiacaspia* Eichwald, 1829. – Grossu: 139, text fig. 19B.

1977 *Hypaniscaspiaassalae* Tadjalli-Pour: 99, pl. 1, fig. 4.

1977 *Hypaniscaspianahali* Tadjalli-Pour: 99, pl. 1, fig. 6.

2013 Adacna (Monodacna) caspiacaspia (Eichwald, 1829). – Kijashko in Bogutskaya et al.: 380, fig. 154.

2016 Adacna (Monodacna) caspiacaspia (Eichwald, 1829). – Vinarski and Kantor: 67.

**Status.** Pontocaspian species, endemic to Caspian Sea.

**Type locality.** “Caspium mare” [Caspian Sea].

**Distribution.** Caspian Sea.

**Taxonomic notes.** The *Monodacnacaspia* group (*M.caspia*, *M.filatovae*, and *M.knipowitschi*) comprises three (sub-) species that all share the relatively convex and rounded shell and well-defined ribbing. These species have been described from different areas and habitats in the Caspian Sea and have been morphologically characterised by Kijashko in Bogutskaya et al. (2013). However, neither morphological analyses of intermediate populations nor genetic analyses have been performed to clarify if the three taxa are distinct or ecomorphs of a single species. We are therefore uncertain whether *M.filatovae* and *M.knipowitschi* should be maintained.

**Conservation status.** Not assessed.

***Monodacnacolorata* (Eichwald, 1829)**

*1829 *G.* [*lycymeris*] *colorata* Eichwald: 279–280, pl. 5, fig. 4a, b.

1838 *Adacnacolorata* m. – Eichwald: 169–170.

?1838 *Monodacnapontica* Eichwald: 168–169.

1926 Monodacnacoloratavar.ialpugensis Borcea: 452, pl. 15, fig. 16.

1926 Monodacnacoloratavar.angusticostata Borcea: 452–453, pl. 15, figs 27, 28, pl. 16, figs 90, 91, pl. 18, figs 143, 169, 173, pl. 21, fig. 7.

1926 *Adacna Luciae* Borcea: 469–471, pl. 18, figs 146, 148–149, 151–153, pl. 21, figs 8, 9.

1952 *Monodacnacolorata* (Eichwald, 1829). – Zhadin: 351, fig. 328.

?1972 *Hypaniscaspiagrossui* Scarlato and Starobogatov: 214, pl. 4, fig. 1a, b.

1973 *Hypaniscaspiagrossui* Scarlato & Starobogatov, 1971. – Grossu: 140, text fig. 21, pl. 1, fig. 8.

1973 *Hypanisangusticostataangusticostata* Borcea, 1926. – Grossu: 141, pl. 1, fig. 12.

1973 *Hypanisluciae* Borcea, 1926. – Grossu: 138, text fig. 18.

1973 *Hypanisialpugensis* Borcea, 1926. – Grossu: 142, fig. 24, pl. 1, figs 9, 10.

1973 *Hypaniscolorata* Eichwald, 1829. – Grossu: 142–143, fig. 25, pl. 1, figs 13–15.

1973 *Hypanispontica* Eichwald, 1838. – Grossu: 143, fig. 26, pl. 1, fig. 11.

2006a *Hypaniscolorata* (Eichwald, 1829). – Munasypova-Motyash: 42–43.

?2006a *Hypanispontica* (Eichwald, 1838). – Munasypova-Motyash: 43–44.

?2006a *Hypanisangusticostataangusticostata* (Borcea, 1926). – Munasypova-Motyash: 44.

2009 *Monodacnapontica* Eichwald, 1838. – Popa et al.: 10, text fig. 2.

2009 *Monodacnacolorata* Eichwald, 1829. – Popa et al.: 10–11, text fig. 3.

2012 *Hypaniscolorata* (Eichwald, 1829). – Popa et al.: 153, 154.

2012 *Hypanisangusticostata* (Borcea, 1926). – Popa et al.: 153, 154.

2013 Adacna (Monodacna) colorata (Eichwald, 1829). – Kijashko in Bogutskaya et al.: 383, fig. 158.

2016 Adacna (Monodacna) angusticostata (Borcea, 1926). – Vinarski and Kantor: 66.

2016 Adacna (Monodacna) grossui (Scarlato et Starobogatov, 1972). – Vinarski and Kantor: 67.

2016 Adacna (Monodacna) ialpugensis (Borcea, 1926). – Vinarski and Kantor: 68.

**Status.** Pontocaspian species, native to Black Sea Basin (including lower Danube River), invasive in Caspian Sea and Volga River.

**Type locality.** “Hypanin fluvium, ad nigrum usque mare” [Lower course of the Yuzhnyi Bug River, all the way to the Black Sea, Ukraine].

**Distribution.** Native to all Black Sea Basin Pontocaspian habitats and lower courses of adjacent rivers such as the Danube, Dnieper, and Dniester; invasive in Caspian Sea Basin and lower Volga, as well as Lake Balkhash (Kazakhstan). Occurs hundreds of kilometres upstream in major tributaries (Danube: Popa et al. 2009; recent observations in Volga River upstream Volgograd by MV and AFS).

**Taxonomic notes.***Monodacnacolorata* appears to be a morphologically very variable species. Here, we propose to synonymise several local Black Sea species with this taxon. Given the difficulty to distinguish relatively flat shells typically associated with *M.colorata* from the more convex shells typically associated with *M.pontica* in, e.g., Lake Razim (Romania) and the apparent lack of genetic differentiation of convex specimens from *M.colorata* we assume that *M.pontica* is a synonym of *M.colorata*. Shell differences have been attributed to substrate differences. Further investigations to confirm the synonymy are required. *Monodacnaangusticostata* was synonymised by Popa et al. (2012) based on molecular evidence, even though some morphological distinction was reported from *M.colorata*, which they attributed to differential habitat preference (sediment type).

**Conservation status.** Not assessed.

***Monodacnafilatovae* (Logvinenko & Starobogatov, 1967)**

1876 *Cardiumcaspium*, Eichw. – Grimm: 134–136 [pars].

*1967 *Hypaniscaspiafilatovae* Logvinenko and Starobogatov: 231.

1973 *Hypaniscaspiafilatovae* Logvinenko & Starobogatov, 1967. – Grossu: 139, text fig. 19a.

2013 Adacna (Monodacna) caspiafilatovae (Logvinenko & Starobogatov, 1967). – Kijashko in Bogutskaya et al.: 381, fig. 155, photo 52.

2016 Adacna (Monodacna) caspiafilatovae (Logvinenko & Starobogatov, 1967). – Vinarski and Kantor: 67.

**Status.** Pontocaspian species, endemic to Caspian Sea. Uncertain whether it concerns a morph of *M.caspia*.

**Type locality.** Gulf of Baku, Caspian Sea, Azerbaijan.

**Distribution.** Southern Caspian Sea Basin.

**Taxonomic notes.** See remarks under *Monodacnacaspia* above for uncertain status of *M.filatovae*.

**Conservation status.** Not assessed.

***Monodacnaknipowitschi* (Logvinenko & Starobogatov, 1966)**

*1966a *Hypaniscaspiaknipowitschi* Logvinenko & Starobogatov: 15, fig. 2.

1973 *Hypaniscaspiaknipowitschi* Logvinenko & Starobogatov, 1967. – Grossu: 140, text fig. 20.

2013 Adacna (Monodacna) caspiaknipowitschi (Logvinenko & Starobogatov, 1966). – Kijashko in Bogutskaya et al.: 381–382, figs 152, 153, photo 53.

2016 Adacna (Monodacna) caspiaknipowitschi (Logvinenko & Starobogatov, 1966). – Vinarski and Kantor: 67.

**Status.** Pontocaspian species, endemic to Caspian Sea. Uncertain whether it concerns a morph of *M.caspia*.

**Type locality.** Middle Caspian Sea Basin.

**Distribution.** Caspian Sea (middle and southern basins). This species was mentioned from depths between 200 and 300 m in the South Caspian Basin of Azerbaijan (Mirzoev and Alekperov 2017, who reported the species as *Hypaniscaspiaknipowitchi*).

**Taxonomic notes.** See remarks under *Monodacnacaspia* above for uncertain status of *M.knipowitschi*.

**Conservation status.** Not assessed.

***Monodacnapolymorpha* (Logvinenko & Starobogatov, 1967)**

*1967 *Hypanisangusticostatapolymorpha* Logvinenko & Starobogatov, 1967: 232.

1973 *Hypanisangusticostatapolymorpha* Logvinenko & Starobogatov, 1967. – Grossu: 141, fig. 22, pl. 1, fig. 7.

2013 Adacna (Monodacna) polymorpha (Logvinenko & Starobogatov, 1967). – Kijashko in Bogutskaya et al.: 383–384, fig. 159, photo 54.

2016 Adacna (Monodacna) polymorpha (Logvinenko & Starobogatov, 1967). – Vinarski and Kantor: 68.

**Status.** Pontocaspian species, endemic to Caspian Sea. Status uncertain.

**Type locality.** Central part of northern Caspian Sea.

**Distribution.** Northern Caspian Sea.

**Taxonomic notes.** See remarks under *M.albida* for uncertain species status.

**Conservation status.** Not assessed.

***Monodacnasemipellucida* (Logvinenko & Starobogatov, 1967)**

*1967 *Hypanissemipellucida* Logvinenko & Starobogatov: 232–233.

1973 *Hypanissemipellucida* Logvinenko & Starobogatov, 1967. – Grossu: 144, text fig. 27.

2013 Adacna (Monodacna) semipellucida (Logvinenko & Starobogatov, 1967). – Kijashko in Bogutskaya et al.: 384, fig. 161, photo 55.

2016 Adacna (Monodacna) semipellucida (Logvinenko & Starobogatov, 1967). – Vinarski and Kantor: 68–69.

**Status.** Pontocaspian species, endemic to Caspian Sea.

**Type locality.** Off Tokmak Cape (also as Toqmaq Müyis), southern Kazakhstan, Caspian Sea.

**Distribution.** Middle Caspian Sea.

**Conservation status.** Not assessed.

#### Family Semelidae Stoliczka, 1870

***Abrasegmentum* (Récluz, 1843)**

°1836 *Erycinaovata* Philippi: 13, pl. 1 fig. 13 [**non***Erycinaovata* Gray, 1825].

*1843 *Syndosmyasegmentum* Récluz: 365–366.

1969 *Abraovata* (Phil.). – Logvinenko and Starobogatov: 339, fig. 355, pl. 5, fig. 12.

2013 *Abrasegmenta* (Récluz, 1843). – Kijashko in Bogutskaya et al.: 391, fig. 165.

2015 *Abraovata* (Philippi, 1836). – Latypov: 240.

**Status.** Invasive Pontocaspian species.

**Type locality.** Mediterranean coast near Taranto (Italy).

**Distribution.** Mediterranean, Black Sea coastal regions, Sea of Azov, Caspian Sea, Aral Sea.

**Taxonomic notes.** This species has been reported in much of the 20^th^ century literature as *Abraovata* (Philippi, 1836), which is invalid since the original name (*Erycinaovata* Philippi, 1836) represents a junior primary homonym of *Erycinaovata* Gray, 1825.

**Remarks.** The first transfer of *Abrasegmentum* into the Caspian Sea occurred in 1947–1948, and the species has not been detected since 1955 (Latypov, 2015).

**Conservation status.** Not assessed.

#### Family Cyrenidae Gray, 1840

***Corbiculafluminalis* (Müller, 1774)**

*1774 *Tellinafluminalis* Müller: 205–206.

1952 *Corbiculafluminalis* (Müller, 1774). – Zhadin: 317, fig. 283.

2012 *Corbiculafluminalis* (Müller, 1774). – Welter-Schultes: 15, unnumbered text figures.

2016 *Corbiculafluminalis* (O.F. Müller, 1774). – Nabozhenko and Nabozhenko: 62, text fig. 1(3, 4).

2016 *Corbiculafluminalis* (O.F. Müller, 1774). – Vinarski and Kantor: 80.

**Status.** Native/Invasive Pontocaspian species.

**Type locality.** Euphrates River.

**Distribution.** Native to large parts of western Asia (including southern Caspian river systems) and northern Africa, introduced in 1939 to southern North America and in 1980 from there to Europe (Seddon and Van Damme 2016). The species has been recently recorded from the Caspian Dagestan coast (Nabozhenko and Nabozhenko 2016).

**Remarks.** This species has been native to south Caspian rivers including the Kura river system (Zhadin 1952) and has expanded several times in the Late Pleistocene into the Caspian Sea, where in time intervals it survived in proximal lacustrine habitats. A recent introduction and expansion of the species has been recorded in the Kizlyarsky Gulf in Dagestan (Nabozhenko and Nabozhenko 2016) and the strong increase in fresh material found around the gulf in subsequent years, including whole specimens (AS Gasanova, Makhachkala, pers. comm.) suggests the species may have established there.

**Conservation status.** Least Concern (Seddon and Van Damme 2016).

#### Family Dreissenidae Gray, 1840

**Remarks.** Pontocaspian dreissenid taxonomy suffers from a lack of coordinated shell and DNA analyses. A large part of our considerations relies on the work of Rosenberg and Ludyanskiy (1994) who examined and illustrated all type material of Pontocaspian *Dreissena*.

***Dreissenabugensis* Andrusov, 1897**

*1897 *Dreissensiabugensis* Andrusov: 285–286, pl. 15, figs 31–37.

1972 *Dreissenarostriformisbugensis* (Andrusov, 1897). – Scarlato and Starobogatov: 232–233, pl. 6, fig. 16.

1994 *Dreissenabugensis* (Andrusov, 1897). – Rosenberg and Ludyanskiy: 1479–1480, fig. 1a–e.

2013 *Dreissenabugensis* (Andrusov, 1897). – Kijashko in Bogutskaya et al.: 331, fig. 119.

2016 *Dreissenabugensis* (Andrusov, 1897). – Vinarski and Kantor: 78.

**Status.** Until mid-20^th^ century endemic to northern Black Sea liman coast, since then invasive elsewhere in Black Sea Basin, Volga catchment, western Europe, and North America.

**Type locality.** Bug Liman near Nikolaev, Ukraine.

**Distribution.** Endemic to western Ukrainian liman coast, introduced in Danube Delta, Azov Sea, Volga catchment, western and central Europe, and North America (Orlova et al. 2005, Coughlan et al. 2017).

**Taxonomic notes.** This species has been considered as a subspecies of *D.rostriformis* (Deshayes, 1838) by some authors (e.g., Orlova et al. 2005), yet we follow the argumentation of Kijashko in Bogutskaya et al. (2013) to consider it as a distinct species. The proposed synonymy of Caspian *D.rostriformis* (= *D.grimmi*) and Black Sea *D.bugensis* by Stepien et al. (2013) is discussed below under *D.grimmi*.

**Conservation status.** Least Concern (von Rintelen and Van Damme 2011a).

***Dreissenacaspia* Eichwald, 1855**

*1855 *Dreissenacaspia* Eichwald: 311–312, pl. 10, figs 19–21.

1969 *Dreissenacaspia* (Eichw.). – Logvinenko and Starobogatov: 316–318, fig. 341(2).

1994 *Dreissenacaspia* Eichwald, 1855. – Rosenberg and Ludyanskiy: 1482, fig. 3e, f.

2013 *Dreissenacaspia* Eichwald, 1855. – Kijashko in Bogutskaya et al.: fig. 109.

2016 Dreissena (Dreissena) caspiacaspia Eichwald, 1855. – Vinarski and Kantor: 76.

**Status.** Caspian endemic, probably extinct.

**Type locality.** Chistyi Bank and Cheleken Island, Caspian Sea, Russia.

**Distribution.** Caspian Sea and Aral Sea, probably extinct.

**Taxonomic notes.** The species is commonly subdivided into a Caspian subspecies (*D.caspiacaspia*) and an Aral Sea subspecies (*D.caspiapallasi* Andrusov, 1897). However, syntypes of the latter illustrated in Rosenberg and Ludyanskiy (1994, fig. 3f) show a broad and keeled *Dreissena* that has major morphological characters in common with *D.polymorpha/elata* rather than *D.caspia*. Filippov and Riedel (2009) reported *Dreissenacaspia* from Holocene core deposits of Aral Sea, but given the juvenile status of their material they noted they were uncertain whether it might comprise *D.polymorpha*. *Dreissenacaspia* was reported alive from the remaining “small Aral Sea” by Plotnikov et al. (2016). However, this latter record concerns more likely *D.polymorpha* and needs confirmation. Andreeva and Andreev (2003) mentioned that this subspecies has not been found in the Aral Sea since 1989.

**Conservation status.** Critically endangered, possibly extinct (von Rintelen and Van Damme 2011b).

***Dreissenaelata* Andrusov, 1897**

*1897 Dreissensiapolymorphavar.elata Andrusov: 353, pl. 20, fig. 25.

1969 *Dreissenaelata* (Andr.). – Logvinenko and Starobogatov: 316, fig. 341(1).

1994 *Dreissenaelata* Andrusov, 1897. – Rosenberg and Ludyanskiy: 1482, fig. 3g.

2013 *Dreissenaelata* (Andrusov, 1897). – Kijashko in Bogutskaya et al.: fig. 108.

2016 Dreissena (Dreissena) elata (Andrusov, 1897). – Vinarski and Kantor: 76.

**Status.** Pontocaspian species, endemic to the Caspian Sea, probably extinct. Species status uncertain.

**Type locality.** Kuuli Cape, Dazmyk, Apsheron Peninsula, Azerbaijan (Vinarski and Kantor 2016).

**Distribution.** Caspian Sea. Probably extinct.

**Taxonomic notes.***Dreissenaelata* has morphological features in common with *D.polymorpha*, including a relatively wide shell and a well-pronounced keel located close to the ventral margin. However, the *D.elata* shell is in general wider, flatter, and has a more rounded abapical margin even though shell characters are higly variable. *Dreissenaelata* has been reported from areas in the Caspian Sea with salinities well above 5 ‰, which is unusual for *D.polymorpha* elsewhere. We are uncertain whether *D.elata* might be a sibling species. Its apparently distinct morphology and autecological preferences suggest it is different from *D.polymorpha*, but it will require molecular comparison to investigate whether it concerns a mere morph that has undergone “ecological release” (Kohn 1972) or is a different species. However, no living specimens of *D.elata* have been recorded since 1957 (Kostianoy and Kosarev 2005) when its Caspian habitats were invaded by *Mytilasterminimus*.

**Conservation status.** Not assessed. It was reported as extinct by Kostianoy and Kosarev (2005, and references therein). If *D.elata* is accepted as a valid species, it might qualify for the same conservation status as *D.caspia* (critically endangered, possibly extinct; von Rintelen and Van Damme 2011b).

***Dreissenagrimmi* (Andrusov, 1890)**

Fig. 4b

1877 *DreyssenaBrardii* var. *caspia* Grimm: 74–75 [**non***Dreissenacaspia* Eichwald, 1855].

*1890 *Dr.* [*eissena*] *Grimmi* Andrusov: 233 [**nom. nov.** pro *Dreissenacaspia* Grimm, 1877, **non** Eichwald, 1855].

1897 *DreissensiaGrimmi* Andrus. – Andrusov: 279–282, pl. 16, figs 16–18.

1897 Dreissensiarostriformisvar.distincta Andrusov: 273–278, pl. 14, figs 18–24.

1897 *Dreissensia Tschaudae* var. *pontocaspica* Andrusov: 294–297, pl. 9, figs 27–32, pl. 15, figs 29, 30.

1966a *Dreissenarostriformiscompressa* Logvinenko and Starobogatov: 15–16, fig. 3.

1969 *Dreissenarostriformisgrimmi* Andr. – Logvinenko and Starobogatov: 318, fig. 341(3).

1969 *Dreissenarostriformispontocaspica* (Andr.). – Logvinenko and Starobogatov: 319, fig. 341(6).

1994 *Dreissenarostriformis* (Deshayes, 1838). – Rosenberg and Ludyanskiy: 1477–1479, figs 1f, 2a–j [**non***Mytilusrostriformis* Deshayes, 1838].

2013 *Dreissenarostriformis* (Deshayes, 1838). – Kijashko in Bogutskaya et al.: 330 [**non** Deshayes, 1838].

2013 *D.* [*reissena*] *rostriformis compressa* Logvinenko & Starobogatov, 1966. – Kijashko in Bogutskaya et al.: 331, fig. 117a, photo 38.

2013 *D.* [*reissena*] *rostriformis distincta* (Andrusov, 1897). – Kijashko in Bogutskaya et al.: 331, fig. 117c.

2013 *D.* [*reissena*] *rostriformis grimmi* (Andrusov, 1890). – Kijashko in Bogutskaya et al.: 331, fig. 117b.

2013 *D.* [*reissena*] *rostriformis pontocaspica* (Andrusov, 1897). – Kijashko in Bogutskaya et al.: 331, fig. 117d.

**Status.** Caspian Sea endemic.

**Type locality.** Caspian Sea.

**Distribution.** Middle to southern Caspian Sea basins. This species was mentioned from depths between 200 and 400 m in the South Caspian Basin of Azerbaijan (Mirzoev and Alekperov 2017, who reported the species as *D.rostriformiscompressa*) and found living offshore Aktau (Kazakhstan) in 2017 below 20 m water depth.

**Taxonomic notes.** This Caspian species is very often cited as *Dreissenarostriformis*. Rosenberg and Ludyanskiy (1994: 1497) discuss the uncertainties of this attribution but state that “*D. pontocaspica, D. distincta, D. compressa*, and *D. grimmi* are synonyms of *D. rostriformis*” even though they find “some justification for maintaining a distinction between an extinct subspecies, *D.rostriformisrostriformis* and a living one, for which *D.rostriformisgrimmi* is the oldest name”. Their figure of the lectotype of *D.rostriformis* (Rosenberg and Ludyanskiy 1994: fig. 2a), which derives from Pliocene deposits of the Black Sea Basin, concerns a relative small, thick-shelled, and low *Dreissena* with a pointed beak and lacking a keel. On interior view, the shell area outside the pallial line is thick. Deshayes’s lectotype has several characters in common with modern Caspian *D.rostriformis* and the closely related Black Sea Basin *D.bugensis*. Yet, the Pliocene form has a broader umbonal area that results in a more subquadrangular shape, which is different from the modern Caspian *Dreissena* that have tear-drop to pear-shaped shells. The subquadrangular shape of Deshayes’s material is even more pronounced in the pallial line on the shell’s interior, a feature not seen in any modern Caspian material. The Pliocene Black Sea *D.rostriformis* has its general shape in common with Apsheronian (Early Pleistocene) Caspian dreissenids referred to as *D.carinatocurvata* as illustrated in Kolesnikov (1950, pl. 14, figs 14–16). Hence, we conclude that the recent Caspian species should be treated different from Pliocene *D.rostriformis* and the name *D.grimmi* should be applied instead.

Various subspecies have been attributed to Caspian *Dreissenarostriformis* (see, e.g., Kijashko in Bogutskaya et al. 2013 for a synonymy list). Even though morphological differences appear to be large, intermediates are known between the morphs. Stepien et al. (2013) reviewed molecular evidence for species boundaries within *Dreissena*. They concluded that (1) all Caspian Sea forms that have been mentioned in literature as (sub-) species of *D.rostriformis* (= *D.grimmi*) are one and the same species and (2) there is not enough molecular evidence and great difficulty in morphology to separate the Caspian species from the Black Sea Basin *D.bugensis*. We agree with the first point made by Stepien et al. (2013); all forms reported from the middle and southern Caspian Sea basins appear to be mere morphs of a single species, a feature also noted by Rosenberg and Ludyanskiy (1994). However, we disagree with their second proposal. *Dreissenabugensis* and *D.grimmi* have non-overlapping ecological tolerances and are separated geographically (Rosenberg and Ludyanskiy 1994). This fact together with the very limited but consistent genetic differentiation suggests that it may concern very recently evolved sister species. In the early 1980s, *D.bugensis* was introduced in the Volga (Zhulidov et al. 2005) and since then spread from there to central and western Europe and North America. So far, *Dreissenabugensis* has only been reported from the Volga itself and its delta but not from the northern Caspian Sea Basin. If it would be conspecific with the middle-southern Caspian species, which lives at higher salinities and deeper habitats, we would expect that the invasive populations in the north would have been blended with the Caspian population in the south. With no such intermediate populations found so far we consider both taxa as viable species.

**Conservation status.** Least Concern (for *Dreissenarostriformis*; von Rintelen and Van Damme 2011c).

***Dreissenapolymorpha* (Pallas, 1771) s.l.**

*1771 *Mytiluspolymorphus* Pallas: 368, 435, 478.

1897 *Dreissensia Andrusovi* Andrusov: 374–376 pl. 18, figs 21–23.

1897 *Dreissensia Pallasi* Andrusov: 671–672, pl. 20, figs 33–35.

1897 Dreissensiapolymorphavar.aralensis Andrusov: 354–355.

1897 Dreissensiapolymorphavar.obtusecarinata Andrusov: 354.

1994 *Dreissenapolymorpha* (Pallas, 1771). – Rosenberg and Ludyanskiy: 1480–1482, fig. 3a, b.

1994 *Dreissenapolymorphaaralensis* Andrusov, 1897. – Rosenberg and Ludyanskiy: 1480, fig. 3c.

1994 *Dreissenapolymorphaobtusecarinata* Andrusov, 1897. – Rosenberg and Ludyanskiy: 1481, fig. 3d.

1994 *Dreissenacaspiapallasi* Andrusov, 1897. – Rosenberg and Ludyanskiy: 1482, fig. 3f.

2003 *Dreissenacaspiapallasi* (Andrusov, 1897). – Andreeva and Andreev: 80, fig. 4.1(7–9).

2003 *Dreissenapolymorphaaralensis* (Andrusov, 1897). – Andreeva and Andreev: 79, fig. 4.1(1–3).

2003 *Dreissenaobtusecarinata* (Andrusov, 1897). – Andreeva and Andreev: 80, fig. 4.1(4–6).

2013 Dreissena (Dreissena) polymorpha (Andrusov, 1897). – Kijashko in Bogutskaya et al.: 328, fig 118a [pars, status fig. 118b uncertain].

2016 Dreissena (Dreissena) polymorphapolymorpha (Andrusov, 1897). – Vinarski and Kantor: 75.

?2016 Dreissena (Dreissena) polymorphaandrusovi (Brusina in Andrusov, 1897). – Vinarski and Kantor: 75.

?2016 Dreissena (Dreissena) polymorphaaralensis (Andrusov, 1897). – Vinarski and Kantor: 75.

?2016 Dreissena (Dreissena) polymorphaobtusecarinata (Andrusov, 1897). – Vinarski and Kantor: 76.

?2016 Dreissena (Dreissena) caspiapallasi (Andrusov, 1897). – Vinarski and Kantor: 7.

**Status.** Native Pontocaspian species.

**Type locality.** Volga and Yaik (Ural) rivers, Caspian Sea.

**Distribution.** Eurasian (native and invasive), North America (invasive) rivers, lakes, estuaries, deltas (Rosenberg and Ludyanskiy 1994, Cummings and Graf 2015, Coughlan et al. 2017). Several unique forms/species within this group reported from the Pontocaspian region.

**Taxonomic notes.***Dreissenapolymorpha* has been subject of intense DNA and ecological studies, but rarely were Caspian communities involved. Combined insights into the shell morphology, ecology, and molecular biology has to date not fully resolved several aspects of Pontocaspian records of this species. Occurrences in rivers and deltas of the Pontocaspian region are consistently attributed to *Dreissenapolymorpha*. However, slightly deviating morphs exist(ed) in salinities typically not favoured by *D.polymorpha* elsewhere in the Caspian and Aral seas. A particular form of *Dreissenapolymorpha*, documented by Kijashko in Bogutskaya et al. (2013), viz. *D.polymorphaandrusovi* (his figure 118b) will need further study as it has many morphological similarities with *D.caspia* (including general shape, location of semidiameter, and broad flat shape of hinge platform).

**Conservation status.** Least Concern (Van Damme 2014).

***Mytilopsisleucophaeata* (Conrad, 1831)**

*1831 *Mytilusleucophaeatus* Conrad: 263–264, pl. 11, fig. 13.

2013 *Mytilopsisleucophaeata* (Conrad, 1831). – Kijashko in Bogutskaya et al.: 320, fig. 107.

**Status.** Invasive Pontocaspian species.

**Type locality.** Southern coast of eastern United States.

**Distribution.** Black Sea Basin, Caspian Sea, coasts of western Europe, Caribbean, and northern South America.

**Remarks.** The species, native to the southern coast of North America, was first introduced in Europe in 1835 (Heiler et al. 2010). In the Pontocaspian region, it first appeared in the northern Black Sea Basin in 2002 and was first collected in the Caspian Sea in 2009 (Heiler et al. 2010). It is easily distinguished from Pontocaspian dreissenids by the presence of an aphophysis near the hinge.

**Conservation status.** Least Concern (Cummings 2011).

### Gastropoda

#### Family Neritidae Rafinesque, 1815

***Theodoxusdanubialis* (Pfeiffer, 1828)**

*1828 *Neritadanubialis* Pfeiffer: 48, pl. 8, figs 17, 18.

2009 *Theodoxusdanubialis* (C. Pfeiffer, 1828). – Fehér et al.: figs 2a–k, 4a–c, 5a–c.

2012 *Theodoxusdanubialis* (Pfeiffer, 1828). – Welter-Schultes: 27, unnumbered text figures.

2016 Theodoxus (Theodoxus) danubialis (Pfeiffer, 1828). – Vinarski and Kantor: 156 [and synonyms therein].

**Status.** Accepted native species.

**Type locality.** Danube River, Vienna, Austria.

**Distribution.** Danube River catchment, central to south-eastern Europe, as well as northern Italy (Fehér et al. 2009).

**Taxonomic notes.** The latest phylogenetic data supports a sister relationship between *Theodoxusdanubialis* and the clade containing *T.fluviatilis* and *T.velox* (AFS, unpublished data). Some authors believe *T.danubialis* and *T.prevostianus* may represent different species given some level of genetic, ecological, and morphological differentiation (Fehér et al. 2009, Welter-Schultes 2012; but see also Bandel 2001). More recent unpublished results may suggest that the genetic level of differentiation between these species is more indicative of intraspecific diversity within a single species (AFS, unpublished data).

**Conservation status.** Least Concern (Tomovic et al. 2010).

***Theodoxusfluviatilis* (Linnaeus, 1758)**

*1758 *Neritafluviatilis* Linnaeus: 777.

1865 Theodoxusfluviatilisvar.subthermalis Issel: 22–23.

1886 *Neritinaeuxina* Clessin: 55.

1908 Neritinadanubialisvar.danasteri Lindholm: 214–215.

?1972 *Theodoxusdniestroviensis* Put’: 80–82, text fig. 5.

?1999 *Th.dniestroviensis*Put’, 1972. – Anistratenko et al.: 19, figs 4, 8.

1999 *Th.fluviatilis* (Linnaeus, 1758). – Anistratenko et al.: 13–15, figs 3, 4.

2005 *Theodoxusfluviatilis* (Linnaeus, 1758). – Anistratenko: 7–8, text figs 3, 4.

2012 *Theodoxuseuxinus* (Clessin, 1886). – Welter-Schultes: 27, unnumbered text figures.

2012 *Theodoxusfluviatilis* (Linnaeus, 1758). – Welter-Schultes: 28, unnumbered text figures.

2015 *Theodoxusfluviatilis* (Linnaeus, 1758). – Glöer and Pešić: 88–91, figs 1, 3–5, 9, 13–34.

2016 Theodoxus (Theodoxus) fluviatilis (Linnaeus, 1758). – Vinarski and Kantor: 154–155 [pars, excluding synonyms *sarmatica* and *velox*].

2016 Theodoxus (Theodoxus) euxinus (Clessin, 1886). – Vinarski and Kantor: 155.

2016 Theodoxus (Theodoxus) subthermalis (Bourguignat in Issel, 1865). – Vinarski andKantor: 157–158.

**Status.** Accepted native species.

**Type locality.** Near Uppsala, Sweden. The lectotype was designated by Anistratenko (2005).

**Distribution.** Widely distributed all over Europe, Anatolia, and north-western Africa. Within the Pontocaspian region, it is a common component of the lower reaches of Black and Azov Sea drainages (specifically in Bulgaria, Romania, and Ukraine). Towards the east, the species extends at least as far as the Don River system in Russia and the coastal rivers of Georgia, but it is absent from the Caspian system. Records of this species from Iran and western Asia are likely misidentifications (AFS, unpublished data).

**Taxonomic notes.***Theodoxusfluviatilis* exhibits considerable variation in shell colouration and shape (Glöer and Pešić 2015). Unpublished molecular data confirm the synonymy of a number of taxa such as *Theodoxuseuxinus* syn. n., *T.danasteri*, and *T.subthermalis* syn. n., and further suggest the inclusion of *T.saulcyi* and *T.heldreichi* (AFS, unpublished data). A final decision concerning the status of *T.dniestroviensis*Put’, 1972 described from the Dniester River (Rukhotyn village, Khotyn district, Chernivtsi region, Ukraine) is not possible at the moment. Despite appropriate efforts, we were unable to trace the type specimens of this species. Based on the original description and illustration (Put’ 1972) it was considered as a junior synonym of *T.fluviatilis* by Anistratenko et al. (1999) having an unusual colour pattern. *Theodoxusmilachevichi* was described as a subfossil from the Crimean coast. It closely resembles morphotypes of both *T.fluviatilis* and *T.velox* V. Anistratenko in O. Anistratenko et al., 1999 and might be synonym of either species (compare type material illustrated in Kantor and Sysoev 2006). However, the morphological variability of the taxa involved, as well as the lacking possibility of acquiring genetic data for *T.milachevichi*, complicates a decision on the independence or synonymy of this species.

**Conservation status.** Least Concern (Kebapçı and Van Damme 2012).

***Theodoxuspallasi* Lindholm, 1924**

°1838 *Neritinaliturata* Eichwald: 156–157 [**non***Neritinaliturata* Schultze, 1826].

*1924 *Theodoxuspallasi* Lindholm: 33, 34 [**nom. nov.** pro *Neritinaliturata* Eichwald, 1838, **non** Schultze, 1826].

1947 Theodoxus (Theodoxus) pallasivar.nalivkini Kolesnikov: 106, 110.

1976 *Theodoxuspallasi* Lindholm, 1924. – Akramovskiy: 88, text fig. 23, pl. 1, figs 1, 2.

1994 *Theodoxusastrachanicus* Starobogatov in Starobogatov, Filchakov, Antonova and Pirogov: 8–9, fig. 1(1, 2).

1994 *Theodoxusastrachanicus* Starobogatov et al.: 8–9, fig. 1(1, 2).

2009 *Theodoxuspallasi* Lindholm, 1924. – Filippov and Riedel: 70, 72, 74, 76, fig. 4g–i.

2011 *Theodoxusastrachanicus* Starobogatov in Starobogatov, Filchakov, Antonova & Pirogov, 1994. – Anistratenko et al.: 54–55, fig. 1(6).

2012 *Theodoxuspallasi* Lindholm, 1924. – Welter-Schultes: 29, unnumbered text figures.

2016 Theodoxus (Theodoxus) astrachanicus Starobogatov in Starobogatov, Filchakov, Antonova & Pirogov, 1994. – Vinarski and Kantor: 155–156.

2016 Theodoxus (Theodoxus) pallasi (Lindholm, 1924). – Vinarski and Kantor: 156–157 [and synonyms therein].

2017 *Theodoxuspallasi* Lindholm, 1924. – Anistratenko et al.: 221, figs 4, 7, 10, 11.

2018 *Theodoxuspallasi* Lindholm, 1924. – Neubauer et al.: 48–51, fig. 4A–F.

**Status.** Accepted Pontocaspian species, name uncertain.

**Type locality.** “Inter Fucos littoris Derbendensis viva” (living among algae on the shores of Derbent), Dagestan, Russia.

**Distribution.** Present along the Caspian Sea shores, in the Volga River, and the Sea of Azov. Lived until the late 1980s in the Aral Sea but is possibly extinct there now (Andreev et al. 1992, Aladin et al. 1998, Micklin et al. 2014).

**Taxonomic notes.**Eichwald (1838) introduced the species *Neritinaliturata* based on material from the shores of Derbent (Dagestan, Russia, northwestern Caspian Sea). That name is invalid as it is a junior primary homonym of *N.liturata* Schultze, 1826; it was replaced by Lindholm (1924) with *Theodoxuspallasi* (see also Anistratenko et al. 2017). *Theodoxuspallasi* is a widely used name, but a major nomenclatural change might be due. Unpublished molecular data suggest that all *Theodoxus* from the Caspian Sea, Azov Sea, and Armenian lakes Sevan and Yerevan, as well as several mineral springs and streams in the Khorasan provinces of Iran, belong to a single species (AFS, unpublished results). The oldest name available for that group is *Theodoxusmajor* Issel, 1865, described from Lake Sevan in Armenia (originally as variety of the unavailable name *T.schirazensis*). Akramovskiy (1976) noted the similarity of *T.pallasi* and *T.major* and considered the latter as a morphotype of the former. Although he did not explicitly state it, he thereby suggested the two taxa to be synonymous. This view was adopted by Vinarski and Kantor (2016), who listed *major* in synonymy of *pallasi*, although Issel’s (1865) name has priority. The potential synonymy also involves *T.schultzii*. Despite the characteristic appearance of the syntypes, the presence of intermediate morphologies in samples taken on shores of Azerbaijan and Kazakhstan in 2016 and 2017 (pers. obs. OA, VA, FW) indicates a close relationship with *T.pallasi.* The radulae of these two species differ in the relative width of the central and marginal teeth (see Zettler 2007 and compare Anistratenko et al. 2017).

Unfortunately, the types of *T.major*, supposed to be in the Museo Regionale di Scienze Naturali, Torino, are inaccessible at the moment due to museum renovation (E Gavetti, pers. comm., Oct 2018). We refrain from a final conclusion on the synonymy of the species involved until information on the types of all taxa as well as published molecular data are available. For details on the taxonomic relationship between *T.pallasi* and *T.astrachanicus*, see discussion in Anistratenko et al. (2017).

**Conservation status.** Data Deficient (Van Damme and Kebapçı 2014).

***Theodoxusschultzii* (Grimm, 1877)**

*1877 *Neritina Schultzii* Grimm: 77–78, pl. 7, fig. 5, pl. 8, fig. 16.

1909 *Neritina (Ninnia) Schultzei* [sic] Grimm. – Andrusov: 106–107, pl. 6, fig. 38.

?1947 Theodoxus (Ninnia) schultzi [sic] var.jukovi Kolesnikov: 106, 110.

1950 Theodoxus (Ninnia) schultzei [sic] (Grimm). – Kolesnikov: 215–216, pl. 26, figs 12, 13.

1969 *Theodoxusschultzi* [sic] (Grimm, 1877). – Logvinenko and Starobogatov: 344, fig. 357.

?1974 *Theodoxuszhukovi* [sic] Kolesnikov, 1947. – Starobogatov: 255, text fig. 223.

2007 Theodoxus (Theodoxus) schultzii (Grimm, 1877). – Zettler: 249, figs 2–5.

2016 Theodoxus (Theodoxus) schultzii (Grimm, 1877). – Vinarski and Kantor: 157.

**Status.** Pontocaspian species, status uncertain.

**Type locality.** Caspian Sea, in two localities, given by Grimm (1877) as 43°17'N, 01°03'E, 40 fathoms, and 42°48'N, 01°22'E, 48 fathoms. Since the longitude was calculated relative to the geographic position of Baku, situated approximately at 50E, the correct longitude should be about 51°00'E (Vinarski and Kantor 2016).

**Distribution.** Middle and southern Caspian Sea basins, between 15 and 100 m (Logvinenko and Starobogatov 1969).

**Taxonomic notes.** See discussion of *T.pallasi* for notes on the potential synonymy with *T.major* Issel, 1865. The status of *T.jukovi* still requires confirmation (Vinarski and Kantor 2016).

**Conservation status.** Not assessed.

***Theodoxusvelox* V. Anistratenko in O. Anistratenko et al., 1999**

*1999 *Th.* [*eodoxus*] *velox* V. Anistratenko in O. Anistratenko et al.: 17–18, fig. 4(7).

**Status.** Pontocaspian species, name uncertain.

**Type locality.** Dnieper Delta, Zbur’ivka liman, Ukraine.

**Distribution.** This species was believed to be restricted to drainage systems of the northern Black Sea coast (even though the Oskol River lies far from the Black Sea coast), but unpublished molecular data suggest it may be distributed as far north as the eastern part of the Baltic Sea and as far south as Anatolia (AFS, unpublished data).

**Taxonomic notes.** The species was listed as junior synonym of *T.fluviatilis* by Vinarski and Kantor (2016). *Theodoxusvelox* is indeed challenging to differentiate from some regional morphotypes of that species given the overlap in shell patterns. Unpublished molecular data indicate however that *T.velox* belongs to a different molecular clade (AFS, unpublished data). The distribution range of that clade overlaps with the range of *T.sarmaticus* (Lindholm, 1901), which is widely accepted as a junior synonym of *T.fluviatilis* in the literature (e.g., Vinarski and Kantor 2016). A revision of the taxa involved and study of the type material is required to solve the synonymy issues.

**Conservation status.** Not assessed.

#### Family Cochliopidae Tryon, 1866

***Eupaludestrinastagnorum* (Gmelin, 1791)**

*1791 *Helixstagnorum* Gmelin: 3653.

1975 *Falsihydrobiastreletzkiensis* Chukhchin: 121.

2012 *Heleobiastagnorum* (Gmelin, 1791). – Welter-Schultes: 39, unnumbered text figures.

2012 *Semisalsastagnorum* (Gemlin, 1791). – Kroll et al.: 1520.

**Status.** Accepted, native Pontocaspian or immigrant species.

**Type locality.** Kaasjeswater, Zierikzee, the Netherlands.

**Distribution.** Coastal areas of Europe and the Mediterranean region, extending to North Africa and east to Iran (Glöer 2002). Occurrence in Black Sea according to, e.g., Chukhchin (1975) and in the Caspian Sea (TW, unpublished data).

**Taxonomic notes.** We find the attribution of this species to the genus *Eupaludestrina* unsatisfactory, yet a further revision is required to establish and stabilise the generic attribution as there is considerable confusion. It is commonly classified in the South American genus *Heleobia* (e.g., Prié 2011), whereas Kroll et al. (2012) suggested that this species belongs to the genus *Semisalsa*, a group of European Cochliopidae distinct from *Heleobia*. However, *Semisalsa* is currently listed as junior synonym of *Eupaludestrina* Mabille, 1877 (type species: *Hydrobiamacei* Paladilhe, 1867, by subsequent designation by Kadolsky 2008). Following Kadolsky (2008), Eupaludestrinais currently ranked assubgenusofHeleobia in MolluscaBase (2018), but both the phylogenetic and geographic distinction of the European and American species suggest separation on the genus level.

**Remarks.** It is unclear whether the species is native to the Pontocaspian area or a recent immigrant.

**Conservation status.** Least Concern (Prié 2011).

#### Family Hydrobiidae Stimpson, 1865

**Remarks.** The Hydrobiidae form the most species-rich mollusc group in the Pontocaspian region. However, in general, useful shell characters are few and highly variable (Wilke and Delicado in press). Descriptions in the past have often been very general, and illustrations of types are notably poor for several of the endemic taxa. A strong tendency of naming large numbers of species has developed throughout the 20^th^ century (e.g., Logvinenko and Starobogatov 1969), but for some groups where morphological and genetic analyses could be performed (e.g., *Caspiohydrobia* spp.) it has been demonstrated that actual species numbers were much lower than the number of species described (Haase et al. 2010). For many of the endemic species, especially in the genus *Turricaspia*, the apparent loss of types, combined with the lack of living material makes it impossible to assess their taxonomic status. Currently, a number of taxonomic works is in progress on the endemic Pontocaspian hydrobiid groups, and some different insights on the genus-level classifications exist. Here, we adopt a conservative approach, mostly based on Neubauer et al. (2018).

##### Subfamily Caspiinae Dybowski, 1913

**Remarks.** The distinction of the genera *Caspia*, *Ulskia*, and *Clathrocaspia* follows Neubauer et al. (2018). The three taxa are differentiated based on details of the protoconch and the expression of teleoconch sculpture. *Caspia* s. s. is characterised by a single distinct but fine spiral keel below the suture. It is usually smooth, yet within the type species some reticulate ornament can be found. Species of *Clathrocaspia* expose a distinctive, reticulate pattern on the teleoconch and a malleate protoconch with faint spiral threads. The aperture of *Clathrocaspia* often develops a distinct flat base. The discinction of the two genera is subject of current research. *Ulskia* also has a malleate protoconch but with more distinct spiral threads; teleoconch sculpture is occasionally present as minute elongate nodules.

***Caspiabaerii* Clessin & Dybowski in Dybowski, 1887**

*1887 *CaspiaBaerii* Clessin & Dybowski in Dybowski: 36–37.

1888 [*Caspia*] *Baerii* n. sp. – Dybowski: 79, pl. 3, fig. 4a, b.

1969 Pyrgula (Caspia) baerii (Cless. & Dyb.). – Logvinenko and Starobogatov: 377, fig. 367(3).

2016 *Caspiabaerii* Clessin & W. Dybowski in W. Dybowski, 1888. – Vinarski and Kantor: 224.

**Status.** Accepted Pontocaspian species.

**Type locality.** Caspian Sea (no details).

**Distribution.** Caspian Sea and possibly Danube Delta (Romania). This species was mentioned from depths between 200 and 400 m in the South Caspian Basin of Azerbaijan (Mirzoev and Alekperov 2017, who reported the species as *Turricaspiabaerii*).

**Taxonomic notes.** The type material is stored in the von Baer collection of Caspian Sea molluscs in the Zoological Museum of Lviv University (Ukraine) and comprises more than a hundred syntypes (Anistratenko et al. 2018). The slender shell, the presence of a fine spiral keel below the suture, and the occasionally weakly reticulated surface distinguish this species from congeners.

**Conservation status.** Not assessed.

***Caspiavalkanovi* (Golikov & Starobogatov, 1966)**

*1966 *P.* [*yrgula*] (*Caspia*) *baeri* [sic] *valkanovi* Golikov & Starobogatov: 354–355, fig. 1(9).

2006 *Caspiavalkanovi* (Golikov & Starobogatov, 1966). – Kantor and Sysoev: 88, pl. 41, fig. N.

**Status.** Pontocaspian species, identity uncertain.

**Type locality.** Off Crimea, station 18, sample 173.

**Distribution.** Endemic to the Black Sea Basin.

**Taxonomic notes.** The identity and status of this subfossil taxon, described from phaseoline silt, are somewhat uncertain. The holotype illustrated in Kantor and Sysoev (2006) is poorly preserved and does not allow a proper assessment of its validity. The general shape and size are indicative of the genus *Caspia* and it looks like a variety that might even be a synonym of *C.baerii*. Furthermore, we are not entirely certain as to the stratigraphic age of the stratigraphic origin of this species. The phaseoline silt is a marine Holocene unit, yet it contains reworked Late Pleistocene Neoeuxinian (Pontocaspian) species (FW, pers. obs.).

**Conservation status.** Not assessed.

***Clathrocaspiabrotzkajae* (Starobogatov in Anistratenko & Prisyazhniuk, 1992)**

*1992 Caspia (Clathrocaspia) brotzkajae Starobogatov in Anistratenko & Prisyazhniuk: 18–19, fig. 2a.

2016 *Caspiabrotzkajae* Starobogatov in Anistratenko & Prisyazhniuk, 1992. – Vinarski and Kantor: 224.

**Status.** Accepted Pontocaspian species.

**Type locality.** Caspian Sea shores of Dagestan, Russia, at ca. 60 m.

**Distribution.** Presently endemic to the Caspian Sea. The species was also recorded from the Holocene of Danube Delta, Ukraine (Anistratenko and Prisyazhniuk 1992).

**Taxonomic notes.** The species differs from its congeners in the bulbous shape, with a ratio of body whorl height/shell height of approx. 3/4, as well as regarding the expanded aperture.

**Conservation status.** Not assessed.

***Clathrocaspiagmelinii* (Clessin & Dybowski in Dybowski, 1887)**

*1887 *CaspiaGmelinii* Clessin & Dybowski in Dybowski: 37–38.

1888 [*Caspia*] *Gmelini* [sic] n. sp. – Dybowski: 79, pl. 3, fig. 7a, b.

1969 Pyrgula (Caspia) gmelinii (Cless. & W. Dyb.). – Logvinenko and Starobogatov: 378, fig. 367(7).

?1969 Pyrgula (Caspia) sowinskyi Logvinenko and Starobogatov: 378, fig. 367(4).

?1977 Pyrgula (Caspia) gaillardi Tadjalli-Pour: 107, pl. 2, fig. 8.

2015 *Caspiagmelinii* Clessin & W. Dybowski, 1887. – Boeters et al.: 178, figs 1–6.

2016 *Caspiagmelinii* Clessin & W. Dybowski in W. Dybowski, 1888. – Vinarski andKantor: 224.

**Status.** Accepted Pontocaspian species.

**Type locality.** Caspian Sea (no details).

**Distribution.** Endemic to the Caspian Sea, recorded from the middle and southern parts. This species was mentioned from depths between 200 and 300 m in the South Caspian Basin of Azerbaijan (Mirzoev and Alekperov 2017, who reported the species as *Turricaspiagmelinii*).

**Taxonomic notes.** The broad shell and the heavily reticulated surface distinguish this species from congeners. *Pyrgulasowinskyi*, from the middle and southern Caspian Sea, and *P.gaillardi*, from the Caspian Sea shore between Astara and Hashtpar (= Talesh), Iran, closely resemble *C.gmelinii* in terms of shell shape, the shape of the aperture, and the distinct reticulate teleoconch sculpture. Very likely, the two species are synonyms of *C.gmelini*. Since the type material of Logvinenko and Starobogatov (1969) has not been found, and the whereabouts of the material of Tadjalli-Pour (1977) is unknown, a re-examination of these species has to be postponed. Here, we suggest to treat them as nomina dubia until more information becomes available.

**Conservation status.** Data Deficient (same for *P.sowinskyi*; Son 2011a, Vinarski 2011o).

***Clathrocaspiaisseli* (Logvinenko & Starobogatov, 1969)**

*1969 Pyrgula (Caspia) isseli Logvinenko & Starobogatov: 378, fig. 367(6).

2016 *Pyrgulaisseli* Logvinenko & Starobogatov, 1968. – Vinarski and Kantor: 239.

**Status.** Pontocaspian species, identity uncertain.

**Type locality.** Southern Caspian Sea (no details), between 40–75 m water depth.

**Distribution.** Endemic to the Caspian Sea.

**Taxonomic notes.** This species hardly differs from *C.pallasii* and might be a junior synonym. Observations on Holocene material from the southern and northern Caspian Sea shores (VA, TN, FW) suggest that the minor differences range within intraspecific variability but further studies (preferentially involving DNA) are required to solve the identity of this taxon. The classification in *Clathrocaspia* is based on the reticulate sculpture typical of that genus.

**Conservation status.** Data Deficient (Vinarski 2011j).

***Clathrocaspiaknipowitschii* (Makarov, 1938)**

*1938 *Caspiagmelini* [sic] var. *Knipowitschii* Makarov: 1058.

?1966 *P.* [*yrgula*] (*Caspia*) *gmelini* [sic] *aluschtensis* Golikov and Starobogatov: 354, fig. 1(8).

1966 *P.* [*yrgula*] (*Caspia*) *makarovi* Golikov and Starobogatov: 353–354, fig. 1(5).

?1987 *Caspiagmeliniistanislavi* Alexenko and Starobogatov: 33, fig. 1.

1992 Caspia (Clathrocaspia) knipowitchi Makarov, 1938. – Anistratenko and Prisyazhniuk: 19, fig. 2b.

2006 *Caspiaknipowitchi* [sic] Makarov, 1938. – Kantor and Sysoev: 87–88, pl. 41, fig. J.

2006 *Caspiamakarovi* (Golikov et Starobogatov, 1966). – Kantor and Sysoev: 88, pl. 41, fig. L.

2013 *Caspiaknipowitchii* [sic] Makarov, 1938. – Anistratenko: 53–55, figs 1A–I, 3A–D, 5A–D.

2013 *Caspiamakarovi* (Golikov & Starobogatov, 1966). – Anistratenko: 56–59, figs 2A–E, 3E.

2016 *Caspiaknipowitchi* [sic] Makarov, 1938. – Vinarski and Kantor: 224.

2016 *Caspiamakarovi* (Golikov & Starobogatov, 1966). – Vinarski and Kantor: 225.

?2016 *Caspiastanislavi* Alexenko & Starobogatov, 1987. – Vinarski and Kantor: 225.

**Status.** Accepted Pontocaspian species.

**Type locality.** Ukraine, in the Dniester River (exact locality not specified).

**Distribution.** Azov Sea and northern Black Sea Basin. Known from the Holocene of Danube Delta, Ukraine (Anistratenko and Prisyazhniuk 1992).

**Taxonomic notes.***Clathrocaspiaknipowitschii*, *C.makarovi*, *C.gmelinialuschtensis*, and *C.stanislavi* were all described from the northern margin of the Black Sea. After detailed morphological comparison of *C.knipowitschii* and *C.makarovi* syn. n. and preliminary genetic analyses (TW, unpublished data), we conclude that both taxa should be considered synonyms. Very likely, also *C.gmelinialuschtensis* and *C.stanislavi* are synonyms of *C.knipowitschii*, but a final decision on that matter requires investigation of the type material.

**Conservation status.** Least Concern (same for *C.makarovi*; Son 2011b, c).

***Clathrocaspialogvinenkoi* (Golikov & Starobogatov, 1966)**

*1966 *P.* [*yrgula*] (*Caspia*) *logvinenkoi* Golikov & Starobogatov: 354, fig. 1(7).

2006 *Caspialogvinenkoi* (Golikov & Starobogatov, 1966). – Kantor and Sysoev: 88, pl. 41, fig. I.

2007a Caspia (Clathrocaspia) logvinenkoi (Golikov & Starobogatov, 1966). – Anistratenko: 25–26, fig. 2.

2016 *Caspialogvinenkoi* (Golikov & Starobogatov, 1966). – Vinarski and Kantor: 224–225.

**Status.** Accepted Pontocaspian species.

**Type locality.** Don Delta, Russia.

**Distribution.** Known only from the type locality.

**Taxonomic notes.** The species has distinctive shell characters: broad conical shape with a weak subsutural bulge and apically thickened peristome.

**Remarks.** The type material was collected by Mordukhay-Boltovskoy in 1937 and comprises two specimens, the holotype and the paratype. Three additional specimens were collected from the same region in 2006 (Anistratenko 2007a). The salinity at the type locality ﬂuctuates between freshwater and ca. 1‰.

**Conservation status.** Not assessed. In the fifty years since the description of this species five specimens have been collected; this is likely evidence of its rarity. Known only from two close localities, *C.logvinenkoi* appears to have an extremely narrow distributional range in the Azov–Black Sea Basin, being endemic to the Taganrog province (e.g., Anistratenko 2007a).

***Clathrocaspiamilae* (Boeters, Glöer & Georgiev, 2015)**

*2015 *Caspiamilae* Boeters, Glöer & Georgiev in Boeters et al.: 180–183, figs 9–21.

**Status.** Pontocaspian species, identity uncertain.

**Type locality.** Bulgaria, Danube Island Vardim (43°37'N, 25°28'E).

**Distribution.** Only known from type locality.

**Taxonomic notes.** This species closely resembles *C.knipowitschii* concerning shape, size, and sculpture. According to Boeters et al. (2015), the two species differ in the degree of cover of the umbilicus, the shape of the peristome and the size and number of whorls of the protoconch. Molecular and/or more in-depth morphological and anatomical studies are required to confirm that these apparently minor differences are sufficient to separate the species.

**Remarks.** If the species would be confirmed, it concerns a Pontocaspian species whose distribution currently is outside prime Pontocaspian habitat, yet Boeters et al. (2015) implied they would expect that several of the *Caspia* records from the lower Danube and Razim Lake complex might be attributed to *C.milae* as well. The Razim Lake complex is Pontocaspian habitat.

**Conservation status.** Not assessed.

***Clathrocaspiapallasii* (Clessin & Dybowski in Dybowski, 1887)**

*1887 *Caspia Pallasii* Clessin & Dybowski in Dybowski: 37.

1888 *Caspia Pallasii* n. sp. – Dybowski: 79, pl. 3, fig. 3a, b.

1969 Pyrgula (Caspia) pallasii (Cless. & W. Dyb.). – Logvinenko and Starobogatov: 378, fig. 367(5).

2016 *Pyrgulapallasii* (Clessin & W. Dybowski in W. Dybowski, 1888). – Vinarski and Kantor: 241.

**Status.** Accepted Pontocaspian species.

**Type locality.** Caspian Sea (no details).

**Distribution.** Endemic to the Caspian Sea.

**Taxonomic notes.** This species differs from the other Caspian species *C.gmelinii* in its very slender shape.

**Conservation status.** Not assessed.

***Ulskiabehningi* (Logvinenko & Starobogatov, 1969)**

*1969 Pyrgula (Ulskia) behningi Logvinenko & Starobogatov: 380, fig. 367(13).

2016 *Pyrgulabehningi* Logvinenko & Starobogatov, 1968. – Vinarski and Kantor: 236.

**Status.** Pontocaspian species, identity uncertain.

**Type locality.** Western part of the southern Caspian Sea, in the vicinity of the Kura River mouth, 39°05'N, 49°48'E, 120 m.

**Distribution.** Type locality only.

**Taxonomic notes.** The drawings provided by Logvinenko and Starobogatov (1969) sketch a broad and conical shell. As such, it differs from the more elongate and ovoid *Ulskiaulskii* (Neubauer et al. 2018). A revision is required to clarify its taxonomic status.

**Conservation status.** Data Deficient (Vinarski 2011f).

? ***Ulskiaderzhavini* (Logvinenko & Starobogatov, 1969)**

*1969 Pyrgula (Ulskia) derzhavini Logvinenko & Starobogatov: 379, fig. 367(9).

2016 *Pyrguladerzhavini* Logvinenko & Starobogatov, 1968. – Vinarski and Kantor: 237.

**Status.** Pontocaspian species, identity uncertain.

**Type locality.** Middle and southern Caspian Sea, 45–81 m.

**Distribution.** Type locality only.

**Taxonomic notes.** The species differs from *U.ulskii* and *U.behningi* in the very slender elongate shape and the presence of a subsutural band; this suggests *P.derzhavini* might be likely a member of *Caspia* s.s. A revision is required to clarify its taxonomic status and generic placement.

**Conservation status.** Not assessed.

***Ulskiaulskii* (Clessin & Dybowski in Dybowski, 1887)**

*1887 *CaspiaUlskii* Clessin & Dybowski in Dybowski: 38–39.

1888 [*Caspia*] *Ulskii* n. sp. – Dybowski: 79, pl. 3, fig. 8a, b.

1969 Pyrgula (Ulskia) nana Logvinenko and Starobogatov: 379–380, fig. 367(12).

1969 Pyrgula (Ulskia) schorygini Logvinenko and Starobogatov: 379, fig. 367(11).

2016 *Pyrgulaulskii* (Clessin & W. Dybowski in W. Dybowski, 1888). – Vinarski and Kantor: 244.

2018 *Ulskiaulskii* (Clessin & W. Dybowski in W. Dybowski, 1887). – Neubauer et al.: 52–54, fig. 5A–K [and synonyms therein].

**Status.** Accepted Pontocaspian species.

**Type locality.** Caspian Sea (no details).

**Distribution.** Western part of the Caspian Sea. This species was mentioned from depths between 200 and 400 m in the South Caspian Basin of Azerbaijan (Mirzoev and Alekperov 2017, who reported the species as *Turricaspiaulskii*, *T.schorgyni*, and *T.nana*).

**Taxonomic notes.** This species was recently studied by Neubauer et al. (2018), who considered *P.nana* and *P.schorygini* as its junior synonyms.

**Conservation status.** Not assessed.

##### Subfamily Hydrobiinae Stimpson, 1865

**Remarks.** In addition to the taxa discussed below, the following species of Hydrobiinae have been mentioned from the Black Sea basin (updated statuses after MolluscaBase 2018a): *Hydrobiaaciculina* (Bourguignat, 1876), *H.acuta* (Draparnaud, 1805), *H.euryomphala* (Bourguignat, 1876), *H.mabilli* (Bourguignat, 1876) [currently accepted as *Peringiamabilli*], *H.macei* Paladilhe, 1867 [currently accepted as *Heleobiamacei*], *H.procerula* (Paladilhe, 1869) [currently considered a synonym of *H.acuta*] (Anistratenko et al. 2011). These species were described from the Western Mediterranean and their occurrence in the Black Sea region requires re-investigation; partly the records might be misidentifications of the species of *Ecrobia* listed below or *Eupaludestrina* (Cochliopidae) listed above.

***Ecrobiagrimmi* (Clessin in Dybowski, 1887)**

*1887 *Hydrobiagrimmi* Clessin in Dybowski: 55–56.

1888 [*Hydrobia*] *grimmi* Clessin. – Dybowski: 79, pl. 3, fig. 2.

2009 *Caspiohydrobiagrimmi* (Clessin & Dybowski, 1888). – Filippov and Riedel: 70–72, 74–76, fig. 4a–d.

**Status.** Accepted native Pontocaspian species.

**Type locality.** Caspian Sea (no details).

**Distribution.** Caspian Sea; Aral Sea; salt lakes near Chelyabinsk, Russia (Shishkoedova 2010); Lake Sawa, Iraq (Haase et al. 2010); Arabian (Persian) Gulf (Glöer and Pešić 2012); possibly also northern and central Kazakhstan and Tajikistan (Vinarski and Kantor 2016), however, no molecular data are known to confirm the identity of the Central Asian snails. This species was mentioned from depths between 200 and 500 m in the South Caspian Basin of Azerbaijan (Mirzoev and Alekperov 2017, who reported the species as *Caspiohydrobiacurta* and *C.gemma*).

**Taxonomic notes.** Most of the species that have been assigned to the genus *Caspiohydrobia* Starobogatov, 1970, including its type species, *Pyrgohydrobiaeichwaldiana* Golikov & Starobogatov, 1966, range within the morphological variability of *E.grimmi*. Previous examination of some *Caspiohydrobia* juvenile shells (Filippov and Riedel 2009, Anistratenko 2013, fig. 4A–C) as well as reproductive systems and radula did not find any criteria to support differentiation. Probably, all of the thirty *Caspiohydrobia* species listed by Kantor and Sysoev (2006) and Vinarski and Kantor (2016) for the Caspian Sea are morphotypes of a single species. Prelimary genetic analyses of *Caspiohydrobia* spp. from salt lakes near Chelyabinsk, Russia (TW, unpublished data) support this assumption.

**Conservation status.** Data Deficient (Vinarski 2011b).

***Ecrobiamaritima* (Milaschewitch, 1916)**

*1916 *Hydrobiamaritima* Milaschewitch: 60–61, pl. 2, fig. 34.

1973 *Hydrobiapontieuxini* Radoman: 15–16.

1977 *Ventrosiapontieuxini* (Radoman, 1973). – Radoman: 210, pl. 21, figs 19, 20.

1992 *Pseudopaludinellacygnea* Anistratenko in Anistratenko and Prisyazhniuk: 17, fig. 1a.

1992 *Pseudopaludinellainflata* Anistratenko in Anistratenko and Prisyazhniuk: 17–18, fig. 1b.

1992 *Pseudopaludinellaismailensis* Anistratenko in Anistratenko and Prisyazhniuk: 18, fig. 1c.

2011 *Pseudopaludinellapontieuxini* (Radoman, 1973). – Anistratenko et al.: 78, pl. 3, fig. 4.

2015 *Graecoanatolicayildirimi* Glöer and Pešić: 49–50, figs 10–14.

**Status.** Accepted, Pontocaspian species.

**Type locality.** Black Sea, at Feodosiya and Adler (Crimea, Ukraine).

**Distribution.** Black Sea Basin; northern Aegean Sea; Lake Sarikum, Turkey; northern Adriatic Sea.

**Taxonomic notes.***Hydrobiapontieuxini*, described from the Black Sea coast in Mangalia, Romania, has been considered a synonym of *E.maritima* based on molecular data (Kevrekidis et al. 2005). Herein, we also consider the *Pseudopaludinella* species introduced by Anistratenko and Prisyazhniuk (1992) as junior synonyms of *E.maritima* based on morphological similarities. A proper revision is still pending.

**Conservation status.** Not assessed.

***Ecrobiaventrosa* (Montagu, 1803)**

*1803 *Turboventrosus* Montagu: 317, pl. 12, fig. 13.

2012 *Ecrobiaventrosa* (Montagu, 1803). – Kadolsky: 69–70.

2012 *Hydrobiaventrosa* (Montagu, 1803). – Welter-Schultes: 40, unnumbered text figures.

**Status.** Accepted, immigrant species.

**Type locality.** On the Kent coast (United Kingdom), at Folkstone and Sandwich.

**Distribution.** Widespread along the coastal zones of northern and western Europe, the Mediterranean Sea, the Russia White Sea; introduced into the western Black Sea.

**Taxonomic notes.** Unpublished genetic data (TW) suggest that most previous records of *E.ventrosa* in the Black Sea are likely misidentifications of *E.grimmi*. A notable exception is a recent, genetically confirmed record from Constanța, Romania (Osikowski et al. 2016). Probably, the French species *Paludestrinaarenarum* Bourguignat, 1876, *P.leneumicra* Bourguignat, 1876, *P.paludinelliformis* Bourguignat, 1876, and *Ventrosiacissana* Radoman, 1977, which have been listed for the Black Sea Basin (Anistratenko 1991, Anistratenko and Prisyazhniuk 1992, Anistratenko et al. 2011), are junior synonyms or misidentifications of this species.

**Conservation status.** Least Concern (Van Damme 2011a).

##### Subfamily Pyrgulinae Brusina, 1882

**Remarks.** The genus concepts of Pontocaspian Pyrgulinae follow the revision of Neubauer et al. (2018). Further change is expected in several of the keeled species here listed under ?*Turricaspia* (?*T.aenigma*, ?*T.basalis*, ?*T.dimidiata*, ?*T.pseudobacuana*, and ?*T.pseudodimiata*) that may be grouped in their own genus for which the name *Trachycaspia* Dybowski & Grochmalicki, 1917 (type species: *Rissoadimidiata* Eichwald, 1838) is available. However, such a decision will require further documentation.

***Clessiniolavariabilis* (Eichwald, 1838)**

*1838 *Paludinavariabilis* Eichwald: 151–152.

1838 *Paludina Triton* Eichwald: 152.

1874 *Bithynia*? *Eichwaldi* Martens: 81.

?1887 *CaspiaGrimmi* Clessin and Dybowski in Dybowski: 39

?1888 [*Caspia*] *Grimmi* n. sp. – Dybowski: 79, pl. 3, fig. 5a, b.

1887 *Clessinia Martensii* Clessin and Dybowski in Dybowski: 43.

1888 *Clessinia Martensii* n. sp. – Dybowski: 79, pl. 2, fig. 5.

1902a *Clessiniaahngeri* Westerlund: 45–46.

1966 *P.*[*yrgula*] (*Clessiniola*) *pseudotriton* Golikov and Starobogatov: 356–357, fig. 2(3

?1969 Pyrgula (Caspiella) derbentina Logvinenko and Starobogatov: 374, fig. 366(8).

1969 Pyrgula (Caspiella) ovum Logvinenko and Starobogatov: 374, fig. 366(9).

1969 Pyrgula (Caspiella) trivialis Logvinenko and Starobogatov: 374–375, fig. 366(10).

1987 Turricaspia (Clessiniola) variabilis (Eichwald, 1838). – Alexenko and Starobogatov: 34, text fig. 5.

1987 Turricaspia (Clessiniola) triton (Eichwald, 1838). – Alexenko and Starobogatov: 34, text fig. 3.

1987 Turricaspia (Clessiniola) martensii (Clessin & Dybowski in Dybowski, 1888). – Alexenko and Starobogatov: 34, text fig. 4.

1987 Turricaspia (Clessiniola) bogensis (Küster, 1852). – Alexenko and Starobogatov: 34.

2006 *Turricaspiavariabilis* (Eichwald, 1838). – Kantor and Sysoev: 111, pl. 49, fig. J.

2011 *Turricaspiamartensii* (Clessin & W. Dybowski in W. Dybowski, 1888). – Anistratenko et al.: 86, fig. 3(17).

2011 *Turricaspiatriton* (Eichwald, 1838). – Anistratenko et al.: 85–86, fig. 3(16).

2011 *Turricaspiavariabilis* (Eichwald, 1838). – Anistratenko et al.: 85, fig. 3(15).

2014 *Turricaspiavariabilis*. – Taviani et al.: 4, fig. 3b.

?2016 *Turricaspiaderbentina* (Logvinenko & Starobogatov, 1968). – Vinarski and Kantor: 247.

2016 *Turricaspiamartensii* (Clessin & W. Dybowski in W. Dybowski, 1888). – Vinarski and Kantor: 248.

2016 *Turricaspiaovum* (Logvinenko & Starobogatov, 1968). – Vinarski and Kantor: 248–249.

2016 *Turricaspiapseudotriton* (Golikov & Starobogatov, 1966). – Vinarski and Kantor: 249.

2016 *Turricaspiatriton* (Eichwald, 1838). – Vinarski and Kantor: 250.

2016 *Turricaspiatrivialis* (Logvinenko & Starobogatov, 1968). – Vinarski and Kantor: 250–251.

2016 *Turricaspiavariabilis* (Eichwald, 1838).– Vinarski and Kantor: 251.

2018 *Clessiniolavariabilis* (Eichwald, 1838). – Neubauer et al.: 60–63, fig. 7A–I.

**Status.** Accepted Pontocaspian species.

**Type locality.** At the Volga River mouth near Astrakhan, and towards the Caspian Sea; also in recently lithified fossil limestone at the shores of Dagestan, Russia.

**Distribution.** Caspian Sea, Azov Sea, and northern Black Sea region. This species was mentioned in the South Caspian Basin of Azerbaijan (Mirzoev and Alekperov 2017, who reported the species as *Turricaspiavariabilis, T.derbentica*, and *T.trivialis*).

**Taxonomic notes.**Neubauer et al. (2018) recently demonstrated the high variability of this species. Comparison of available illustrations and descriptions of the species listed in the synonymy list indicates that all of them range within this species’ variability. Consequently, we consider all of them as junior synonyms of *C.variabilis*. A more in-depth review of the type material of the species involved is required to confirm this approach.

The status of *Paludinabogensis* Dubois in Küster, 1852, which was listed as a valid species of *Turricaspia* by Anistratenko and Stadnichenko (1995), is still unclear. That species was described from the Zapadnyi Bug River in Poland and closely resembles *C.variabilis*. It is, however, unlikely that a Pontocaspian species typical of oligohaline conditions occurs so far away in a pure freshwater environment. “*Paludinaeichwaldi* Krynicki, 1837” found in the literature is a nomen nudum. Martens (1874) provided measurements and made the name available, but he listed *Paludinavariabilis* Eichwald, 1838 in synonymy, which has priority. Dybowski (1887) obviously overlooked this and considered *Nematurellaeichwaldi* Krynicki a valid species. We follow Vinarski and Kantor (2016) and consider the species as a junior synonym of *Clessiniolavariabilis*.

**Conservation status.** Least Concern (Cioboiu et al. 2011).

***Laevicaspiaabichi* (Logvinenko & Starobogatov, 1969)**

*1969 Pyrgula (Caspiella) abichi Logvinenko & Starobogatov: 372, fig. 366(3).

2016 *Pyrgulaabichi* Logvinenko & Starobogatov, 1968. – Vinarski and Kantor: 235.

**Status.** Accepted Pontocaspian species.

**Type locality.** Southern and western parts of the Middle Caspian Sea, 36–120 m.

**Distribution.** Middle and South Caspian Basin. This species was mentioned from depths between 200 and 400 m in the South Caspian Basin of Azerbaijan (Mirzoev and Alekperov 2017, who reported the species as *Turricaspiaabichi*).

**Taxonomic notes.** The species differs from the *L.cincta* in its much larger size, the conical shape, the narrower subsutural band, and the larger aperture (compare Neubauer et al. 2018).

**Conservation status.** Data Deficient (Vinarski 2011e).

***Laevicaspiacaspia* (Eichwald, 1838)**

*1838 *Rissoacaspia* Eichwald: 154–155.

non 1888 *Micr.* [*omelania*] *caspia* Eichw. sp. – Dybowski: 78, pl. 1, fig. 1.

?1896 *B.* [*uliminus*] (*Napaeus*?) *goebeli* Westerlund: 188.

1915 *Micromelania* (?) *curta* Nalivkin: 21–22, 31, pl. 6, figs 1, 2 [pars, non figs 3, 4, 7, 9–14].

1915 [*Micromelania* (?) *curta*] var. *plano-convexa* Nalivkin: 22, 31, pl. 6, figs 15–18.

non 1915 *Micromelaniacaspia* Eichw. – Nalivkin: 22, 31, pl. 6, figs 5, 6 [pars, non fig. 8].

non 1917 *Micromelania* (*Turricaspia, Laevicaspia*) *caspia* Eichw. – Dybowski and Grochmalicki: 5–8, 36–38, pl. 1, figs 1–3.

non 1969 *Pyrgulacaspia* (Eichw.). – Logvinenko and Starobogatov: 369–370, fig. 364(1).

2006 *Turricaspiacaspia* (Eichwald, 1838). – Kantor and Sysoev: 106, pl. 49, fig. M.

2016 *Turricaspiacaspia* (Eichwald, 1838). – Vinarski and Kantor: 246.

2018 *Laevicaspiacaspia* (Eichwald, 1838). – Neubauer et al.: 63–66, fig. 8A–K [and synonyms therein].

**Status.** Accepted Pontocaspian species.

**Type locality.** In fossil limestone of Dagestan, Russia.

**Distribution.** Endemic to the Caspian Sea. This species was mentioned from depths between 200 and 500 m in the South Caspian Basin of Azerbaijan (Mirzoev and Alekperov 2017, who reported the species as *Turricaspiacaspia* and *T.curta*).

**Taxonomic notes.** For a detailed discussion about the identity of this species, its synonyms and former misidentifications, see Neubauer et al. (2018).

**Conservation status.** IUCN indicates “Least Concern” (Vinarski 2012), but the true status of this species is highly uncertain.

***Laevicaspiacincta* (Abich, 1859)**

*1859 *Rissoacincta* Abich: 57, pl. 2, fig. 6.

?1887 *CaspiaOrthii* Clessin & Dybowski in Dybowski: 40.

?1888 [*Caspia*] *Orthii* n. sp. – Dybowski: 79, pl. 3, fig. 6.

1969 Pyrgula (Caspiella) cincta (Abich). – Logvinenko and Starobogatov: 372, fig. 366(4).

2006 *Pyrgulacincta* (Abich, 1859). – Kantor and Sysoev: 98, pl. 47, fig. L.

2016 *Pyrgulacincta* (Abich, 1859). – Vinarski and Kantor: 236–237.

2018 *Laevicaspiacincta* (Abich, 1859). – Neubauer et al.: 66–68, fig. 9A–H.

**Status.** Accepted Pontocaspian species.

**Type locality.** Gulf of Baku, Azerbaijan.

**Distribution.** Southern Caspian Sea (Logvinenko and Starobogatov 1969).

**Taxonomic notes.** For a detailed discussion about the identity of this species and its synonym, see Neubauer et al. (2018).

**Conservation status.** Data Deficient (Vinarski 2011g).

***Laevicaspiaconus* (Eichwald, 1838)**

*1838 *RissoaConus* Eichwald: 155.

non 1876 *Eulimaconus*, Eichw?. – Grimm: 154–156, pl. 6, fig. 14.

non 2006 *Turricaspiaconusconus* (Eichwald, 1838). – Kantor and Sysoev: 106, pl. 48, fig. J.

2016 *Turricaspiaconusconus* (Eichwald, 1838). – Vinarski and Kantor: 246–247.

2018 *Laevicaspiaconus* (Eichwald, 1838). – Neubauer et al.: 69–71, fig. 9I–P [and synonyms therein].

**Status.** Accepted Pontocaspian species.

**Type locality.** In fossil limestone of Dagestan, Russia.

**Distribution.** Endemic to the Caspian Sea (Logvinenko and Starobogatov 1969). This species was mentioned from depths between 200 and 300 m in the South Caspian Basin of Azerbaijan (Mirzoev and Alekperov 2017, who reported the species as *Turricaspiaconus*).

**Taxonomic notes.** For a detailed discussion about the identity of this polymorphic species and previous misidentifications, see Neubauer et al. (2018).

**Conservation status.** Data Deficient (Vinarski 2011p).

? ***Laevicaspiaebersini* (Logvinenko & Starobogatov, 1969)**

*1969 Pyrgula (Oxypyrgula) ebersini Logvinenko & Starobogatov: 368, fig. 363(7).

2016 *Pyrgulaebersini* Logvinenko & Starobogatov, 1968. – Vinarski and Kantor: 238.

**Status.** Pontocaspian species, identity uncertain.

**Type locality.** Western part of the middle Caspian Sea, 0–50 m water depth.

**Distribution.** Type locality only.

**Taxonomic notes.** We cannot verify the status of this species given the inadequate descriptions and illustrations and its general resemblance to other species that were described earlier.

**Conservation status.** Data Deficient (Vinarski 2011h).

? ***Laevicaspiaismailensis* (Golikov & Starobogatov, 1966)**

*1966 *P.* [*yrgula*] *ismailensis* Golikov & Starobogatov: 358, fig. 2(11).

2006 *Turricaspiaismailensis* (Golikov & Starobogatov, 1966). – Kantor and Sysoev: 108, pl. 50, fig. A.

2016 *Turricaspiaismailensis* (Golikov & Starobogatov, 1966). – Vinarski and Kantor: 248.

**Status.** Accepted Pontocaspian species.

**Type locality.** Ukraine, Danube Delta, lakes Yalpug and Kugurlui.

**Distribution.** North-western Black Sea Basin (Anistratenko and Stadnichenko 1995).

**Taxonomic notes.** Based on the illustration of the holotype in Kantor and Sysoev (2006), we tentatively place the species in the genus *Laevicaspia*. A more detailed study is necessary to clarify its systematic position.

**Conservation status.** Vulnerable (Son and Cioboiu 2011).

***Laevicaspiakolesnikoviana* (Logvinenko & Starobogatov in Golikov & Starobogatov, 1966)**

*1966 *P.* [*yrgula*] (*Caspiella*) *kolesnikoviana* Golikov & Starobogatov: 357–358, fig. 2(8–9).

1969 *Pyrgula* [(*Caspiella*)] *kolesnikoviana* Logv. & Star. – Logvinenko and Starobogatov: 372, fig. 366(1).

2006 *Pyrgulakolesnikoviana* Logvinenko & Starobogatov in Golikov & Starobogatov, 1966. – Kantor and Sysoev: 100, pl. 47, fig. N.

2016 *Pyrgulakolesnikoviana* Logvinenko & Starobogatov in Golikov & Starobogatov, 1966. – Vinarski and Kantor: 239.

2018 *Laevicaspiakolesnikoviana* (Logvinenko & Starobogatov in Golikov & Starobogatov, 1966). – Neubauer et al.: 71–73, fig. 10A–E, K, N.

**Status.** Accepted Pontocaspian species.

**Type locality.** Caspian Sea, northward of Apsheron Peninsula, north-westward from Kamni Dva Brata Island, 40°47'N, 49°42'E, 30 m water depth.

**Distribution.** Endemic to the Caspian Sea. This species was mentioned from depths between 200 and 400 m in the South Caspian Basin of Azerbaijan (Mirzoev and Alekperov 2017, who reported the species as *Turricaspiakolesnikoviana*).

**Taxonomic notes.** For a detailed discussion about the identity of this species, see Neubauer et al. (2018).

**Conservation status.** Data Deficient (Vinarski 2011k).

***Laevicaspiakowalewskii* (Clessin & Dybowski in Dybowski, 1887)**

*1887 *CaspiaKowalewskii* Clessin & Dybowski in Dybowski: 40–41.

1888 [*Caspia*] *Kowalewskii* n. sp. – Dybowski: 79, pl. 3, fig. 9a–c.

2006 *Pyrgulakowalewskii* (Clessin & W. Dybowski in W. Dybowski, 1888). – Kantor and Sysoev: 100, pl. 47, fig. M.

2016 *Pyrgulakowalewskii* (Clessin & W. Dybowski in W. Dybowski, 1888). – Vinarski and Kantor: 239–240.

**Status.** Accepted Pontocaspian species.

**Type locality.** Caspian Sea (no details).

**Distribution.** Caspian Sea, recorded from southern basin (Logvinenko and Starobogatov 1969) and middle basin (personal observation based on material from Dagestan region, TAN, FW). This species was mentioned from depths between 200 and 300 m in the South Caspian Basin of Azerbaijan (Mirzoev and Alekperov 2017, who reported the species as *Turricaspiakowalewskii*).

**Taxonomic notes.** This species differs from *L.kolesnikoviana* in its bigger size, broader shape, and thinner peristome. *Laevicaspiacincta* can be distinguished based on the stouter shape and the presence of a narrow subsutural band.

**Conservation status.** Not assessed.

***Laevicaspialencoranica* (Logvinenko & Starobogatov, 1969)**

*1969 Pyrgula (Eurycaspia) lencoranica Logvinenko & Starobogatov: 357, fig. 358(14).

2016 *Pyrgulalencoranica* Logvinenko & Starobogatov, 1968. – Vinarski and Kantor: 240.

**Status.** Pontocaspian species, identity uncertain.

**Type locality.** Caspian Sea (no details).

**Distribution.** Caspian Sea (Logvinenko and Starobogatov 1969).

**Taxonomic notes.** Based on the illustrations provided in Kantor and Sysoev (2006), this species differs from *L.cincta* and *L.kowalewskii* in the conical shape and large body whorl. A revision is required to assure its status as distinct species.

**Conservation status.** Not assessed.

***Laevicaspialincta* (Milaschewitch, 1908)**

*1908 *Micromelanialincta* Milaschewitch: 991.

?1966 *P.*[*yrgula*] (*Caspiella*) *azovica* Golikov and Starobogatov: 357, fig. 2(7).

?1966 *P.*[*yrgula*] (*Caspiella*) *boltovskoji* Golikov and Starobogatov: 357, fig. 2(4).

?1966 *P.*[*yrgula*] (*Caspiella*) *crimeana* Golikov and Starobogatov: 358, fig. 2(10).

?1966 *P.*[*yrgula*] (*Caspiella*) *limanica* Golikov and Starobogatov: 357, fig. 2(6).

?1966 *P.*[*yrgula*] (*Caspiella*) *lindholmiana* Golikov and Starobogatov: 357, fig. 2(5).

?1966 *P.*[*yrgula*] (*Laevicaspia*) *iljinae* Golikov and Starobogatov: 358–359, fig. 2(14).

?1966 *P.*[*yrgula*] (*Laevicaspia*) *milachevitchi* Golikov and Starobogatov: 359, fig. 2(15).

?1966 *P.*[*yrgula*] (*Laevicaspia*) *ostroumovi* Golikov and Starobogatov: 358, fig. 2(13).

?1966 *P.*[*yrgula*] (*Turricaspia*) *borceana* Golikov and Starobogatov: 359, fig. 2(16).

?1966 *P.*[*yrgula*] (*Turricaspia*) *nevesskae* Golikov and Starobogatov: 359, fig. 2(17).

?1987 *Turricaspiaabichiphaseolinica* Alexenko and Starobogatov: 33.

?1987 Turricaspia (Caspiella) derbentinaborysthenica Alexenko adn Starobogatov: 34–35, fig. 6.

?1987 Turricaspia (Laevicaspia) grigorievi Alexenko and Starobogatov: 35, fig. 7.

?1987 Turricaspia (Laevicaspia) meneghinianaukrainica Alexenko and Starobogatov: 35, fig. 9.

?2006 *Euxinipyrgulaazovica* (Golikov & Starobogatov, 1966). – Kantor and Sysoev: 95, pl. 44, fig. K.

?2006 *Euxinipyrgulaborysthenica* (Alexenko & Starobogatov, 1987). – Kantor and Sysoev: 95, pl. 44, fig. J.

?2006 *Euxinipyrgulagrigorievi* (Alexenko & Starobogatov, 1987). – Kantor and Sysoev: 95, pl. 44, fig. I.

?2006 *Euxinipyrgulalimanica* (Golikov & Starobogatov, 1966). – Kantor and Sysoev: 95, pl. 44, fig. H.

2006 *Euxinipyrgulalincta* (Milaschewitsch, 1908). – Kantor and Sysoev: 95–96, pl. 45, fig. D.

?2006 *Euxinipyrgulamilachevitchi* (Golikov & Starobogatov, 1966). – Kantor and Sysoev: 96, pl. 45, fig. C.

?2006 *Euxinipyrgulaostroumovi* (Golikov & Starobogatov, 1966). – Kantor and Sysoev: 96, pl. 45, fig. B.

?2006 *Euxinipyrgulaukrainica* (Alexenko & Starobogatov, 1987). – Kantor and Sysoev: 95, pl. 45, fig. A.

?2006 *Turricaspiaboltovskoji* (Golikov & Starobogatov, 1966). – Kantor and Sysoev: 105–106, pl. 48, fig. K.

?2006 *Turricaspiaborceana* (Golikov & Starobogatov, 1966). – Kantor and Sysoev: 106, pl. 49, fig. B.

?2006 *Turricaspiaconuslindholmiana* (Golikov & Starobogatov, 1966). – Kantor and Sysoev: 107, pl. 48, fig. L.

?2006 *Turricaspiacrimeana* (Golikov & Starobogatov, 1966). – Kantor and Sysoev: 107, pl. 48, fig. C.

?2006 *Turricaspiailjinae* (Golikov & Starobogatov, 1966). – Kantor and Sysoev: 108, pl. 49, fig. D.

?2006 *Turricaspianevesskae* (Golikov & Starobogatov, 1966). – Kantor and Sysoev: 109, pl. 49, fig. L.

**Status.** Accepted Pontocaspian species.

**Type locality.** Kotlabukh Lake, Odessa Region, Ukraine (approximately 45°25'35"N, 28°59'41"E).

**Distribution.** Limans and lower reaches of rivers Don, Dnieper, Dniester, and Southern Bug entering the northern Black Sea Basin and the Azov Sea (Taganrog Bay), as well as in coastal lakes Kotlabukh and Yalpug (Vinarski and Kantor 2016). The record of an undescribed subspecies of *T.boltovskoji* from the Caspian Sea mentioned by Anistratenko and Stadnichenko (1995) is probably based on a misidentification.

**Taxonomic notes.**Golikov and Starobogatov (1966) and Alexenko and Starobogatov (1987) introduced a plethora of names for morphologically similar species from the northern Black Sea Basin, partly deriving from subfossil horizons. They differ from *Laevicaspialincta* slightly in the number of whorls and outline shape, but overall range within its morphological variability. Here, we consider them tentatively all junior synonyms of *L.lincta*. Since Starobogatov’s type material is unknown, support for this approach requires collection of new material from the type localities of these taxa. Molecular data confirmed the conspecifity of *L.lincta* and *L.milachevitchi* (Wilke et al. 2007).

**Conservation status.** Least Concern (Son 2011e).

? ***Laevicaspiamarginata* (Westerlund, 1902)**

*1902a *Nematurellamarginata* Westerlund: 45.

2013 *Pyrgulamarginata* (Westerlund, 1902). – Vinarski et al.: 85, fig. 2F.

2016 *Pyrgulamarginata* (Westerlund, 1902). – Vinarski and Kantor: 240.

**Status.** Pontocaspian species, identity uncertain.

**Type locality.** Caspian Sea, “near Krasnojarsk” (Westerlund 1902a). This statement is clearly erroneous since Krasnojarsk is situated in Siberia. Most probably, Westerlund meant Krasnovodsk (nowadays Turkmenbashi) in Turkmenistan (Vinarski et al. 2013).

**Distribution.** Endemic to the Caspian Sea. This species was mentioned from depths between 200 and 300 m in the South Caspian Basin of Azerbaijan (Mirzoev and Alekperov 2017, who reported the species as *Turricaspiamarginata*).

**Taxonomic notes.** The status of this species is uncertain. The illustrations of the type material by Vinarski et al. (2013) suggest a tentative placement in the genus *Laevicaspia*. It shows close similarities with *L.sieversii* (Clessin in Dybowski, 1887). A careful revision of the species is required to clarify its taxonomic status and systematic placement.

**Conservation status.** Not assessed.

***Laevicaspiasieversii* (Clessin in Dybowski, 1887)**

*1887 *Nematurella Sieversii* Clessin in Dybowski: 45–46.

1888 *Nematurella Sieversi* [sic] n. sp. – Dybowski: 78, pl. 2, fig. 1.

**Status.** Pontocaspian species, identity uncertain.

**Type locality.** Caspian Sea (no details).

**Distribution.** Endemic to the Caspian Sea.

**Taxonomic notes.** This species has not been found since its first description, its identity is unclear (Vinarski and Kantor 2016). Judging from the description and drawing in Dybowski (1887), we suggest a systematic placement in *Laevicaspia*. It might be related to *L.conus* (Eichwald, 1838).

**Conservation status.** Not assessed.

? ***Turricaspiaaenigma* (Logvinenko & Starobogatov, 1969)**

*1969 Pyrgula (Celekenia) aenigma Logvinenko & Starobogatov: 375, fig. 366(12).

2016 *Pyrgulaaenigma* Logvinenko & Starobogatov, 1968. – Vinarski and Kantor: 235.

**Status.** Pontocaspian species, identity uncertain.

**Type locality.** Caspian Sea, northward of Apsheron Peninsula, 75 m.

**Distribution.** Type locality only.

**Taxonomic notes.** The identity of this species is unclear. The illustrations of the holotype in Kantor and Sysoev (2006) show a small shell with four whorls, of which the latter two bear a distinct keel. The small size and the relatively large protoconch suggest that the type specimen is a juvenile shell. More specimens (including adult material) are required to shed light on this species’ identity.

**Conservation status.** Not assessed.

***Turricaspiaandrussowi* (Dybowski & Grochmalicki, 1915)**

*1915 Micromelania (Turricaspia) andrussowi Dybowski & Grochmalicki: 125–126, pl. 3, fig. 31a, b.

?1969 Pyrgula (Oxypyrgula) dubia Logvinenko and Starobogatov: 368, fig. 363(5).

?1969 Pyrgula (Oxypyrgula) turkmenica Logvinenko and Starobogatov: 368, fig. 363(6).

2006 *Turricaspiaandrussowi* (B. Dybowski & Grochmalicki, 1915). – Kantor and Sysoev: 104–105, pl. 48, fig. A [pars, excluding synonymy].

2016 *Turricaspiaandrussowi* (B. Dybowski & Grochmalicki, 1915). – Vinarski and Kantor: 245 [pars, excluding synonymy].

2018 *Turricaspiaandrussowi* (B. Dybowski & Grochmalicki, 1915). – Neubauer et al.: 74–76, fig. 11A, BB.

**Status.** Accepted Pontocaspian species.

**Type locality.** Caspian Sea (no details).

**Distribution.** Endemic to the Caspian Sea. The two tentative synonyms were recorded from the western part of the middle Caspian Sea and the eastern part of the southern Caspian Sea, respectively. This species was mentioned from depths between 200 and 500 m in the South Caspian Basin of Azerbaijan (Mirzoev and Alekperov 2017, who reported the species as *T.turkmenica*, *T.dubia*, and *T.andrussowi*).

**Taxonomic notes.** The species was recently investigated by Neubauer et al. (2018). *Pyrguladubia* and *P.turkmenica* are tentatively considered juveniles and thus junior synonyms of this species.

**Conservation status.** Not assessed.

? ***Turricaspiabasalis* (Dybowski & Grochmalicki, 1915)**

*1915 Micromelaniadimidiatavar.basalis Dybowski & Grochmalicki: 131, pl. 3, fig. 36a, b.

1969 Pyrgula (Trachycaspia) laticarinata Logvinenko and Starobogatov: 359, fig. 359(3).

2006 *Pyrgulabasalisbasalis* (B. Dybowski & J. Grochmalicki, 1915). – Kantor and Sysoev: 97, pl. 46, fig. A.

2006 *Pyrgulabasalislaticarinata* Logvinenko & Starobogatov, 1968. – Kantor and Sysoev: 97, pl. 46, fig. B.

2016 *Pyrgulabasalisbasalis* (B. Dybowski & Grochmalicki, 1915). – Vinarski and Kantor: 236.

2016 *Pyrgulabasalislaticarinata* Logvinenko & Starobogatov, 1968. – Vinarski and Kantor: 236.

**Status.** Pontocaspian species, identity uncertain.

**Type locality.** Caspian Sea (no details).

**Distribution.** Middle and southern Caspian Sea (Logvinenko and Starobogatov 1969). This species was mentioned from depths between 200 and 400 m in the South Caspian Basin of Azerbaijan (Mirzoev and Alekperov 2017, who reported the species as *T.laticarinata*).

**Taxonomic notes.** The species is characterised by a massive keel near the lower suture. ?*Turricaspiadimidiata* is distinguished based on its more centrally placed keel. This distinction is tentative and only based on comparison of available illustrations; we are aware of the possibility that these differences might not be diagnostic. Moreover, the keel seems to become stronger with increasing water depth (Starobogatov 1968). *Pyrgulalaticarinata* Logvinenko & Starobogatov, 1969, which differs from *T.basalis* only in the strength of the keels, was considered a junior synonym by Neubauer et al. (2018).

**Conservation status.** Not assessed.

? ***Turricaspiabogatscheviana* (Logvinenko & Starobogatov, 1969)**

*1969 Pyrgula (Oxypyrgula) bogatscheviana Logvinenko & Starobogatov: 367, fig. 363(2).

2016 *Turricaspiabogatscheviana* (Logvinenko & Starobogatov, 1968). – Vinarski and Kantor: 245.

**Status.** Pontocaspian species, identity uncertain.

**Type locality.** Western part of the Caspian Sea.

**Distribution.** Type locality only.

**Taxonomic notes.** The description and drawing of this species provided by Logvinenko and Starobogatov (1969) do not allow an evaluation whether it is a distinct species or synonym of a previously species.

**Conservation status.** Not assessed.

***Turricaspiachersonica* Alexenko & Starobogatov, 1987**

*1987 Turricaspia (Oxypyrgula) chersonica Alexenko & Starobogatov: 35–36, fig. 10.

2016 *Turricaspiachersonica* Alexenko & Starobogatov, 1987. – Vinarski and Kantor: 246.

**Status.** Pontocaspian species, identity uncertain.

**Type locality.** Ukraine, in the Dnieper Delta.

**Distribution.** Type locality only.

**Taxonomic notes.** The status of this species is highly uncertain. The slender conical shell illustrated by Alexenko and Starobogatov (1987) suggest classification in the genus *Turricaspia*, which is otherwise only known from the Caspian Sea.

**Conservation status.** Data Deficient (Son 2011d).

***Turricaspiacolumna* (Logvinenko & Starobogatov, 1969)**

*1969 Pyrgula (Oxypyrgula) columna Logvinenko & Starobogatov: 368, fig. 363(8).

2016 *Pyrgulacolumna* Logvinenko & Starobogatov, 1968. – Vinarski and Kantor: 237.

**Status.** Pontocaspian species, identity uncertain.

**Type locality.** Western part of the southern Caspian Sea.

**Distribution.** Type locality only.

**Taxonomic notes.** The species has not been found since its first description, and the whereabouts of the type material is unknown. Logvinenko and Starobogatov (1969) illustrate a small slender shell with convex whorls. It might well be a juvenile of another species.

**Conservation status.** Not assessed.

***Turricaspiaconcinna* (Logvinenko & Starobogatov, 1969)**

*1969 Pyrgula (Turricaspia) concinna Logvinenko & Starobogatov: 365, fig. 362(3).

2016 *Pyrgulaconcinna* Logvinenko & Starobogatov, 1968. – Vinarski and Kantor: 237.

**Status.** Pontocaspian species, identity uncertain.

**Type locality.** Middle Caspian Sea, 25–80 m.

**Distribution.** Type locality only.

**Taxonomic notes.** The illustrations provided by Logvinenko and Starobogatov (1969) indicate a large conical shell with nine convex whorls and a large, slightly inflated last whorl. These features are reminiscent of *T.meneghiniana* (Issel, 1865). However, *T.concinna* has not been found since its first description. The type material has been very recently detected in the collections of ZIN and awaits further study.

**Conservation status.** Not assessed.

***Turricaspiadagestanica* (Logvinenko & Starobogatov, 1969)**

*1969 Pyrgula (Turricaspia) dagestanica Logvinenko & Starobogatov: 361, fig. 360(3).

2016 *Turricaspiadagestanica* (Logvinenko & Starobogatov, 1968). – Vinarski and Kantor: 247.

**Status.** Pontocaspian species, identity uncertain.

**Type locality.** Western shore of the middle Caspian Sea.

**Distribution.** Middle and south Basin of Caspian Sea. This species was mentioned from depths between 200 and 300 m in the South Caspian Basin of Azerbaijan (Mirzoev and Alekperov 2017).

**Taxonomic notes.** The status of this species is highly uncertain. The illustrations of Logvinenko and Starobogatov (1969) show a slightly distorted shell with weakly convex whorls and a thin line below the suture. We are uncertain whether it might concern a growth aberration of a more common species.

**Conservation status.** Data Deficient (Vinarski 2011r).

***Turricaspiadimidiata* (Eichwald, 1838)**

*1838 *Rissoadimidiata* Eichwald: 156.

?1947 *Turricaspiabakuana* Kolesnikov: 108, 112.

2006 *Pyrguladimidiata* (Eichwald, 1838). – Kantor and Sysoev: 99, pl. 46, fig. K.

?2006 *Pyrgulabakuana* (Kolesnikov, 1947). – Kantor and Sysoev: 97, pl. 47, fig. C.

2016 *Pyrguladimidiata* (Eichwald, 1838). – Vinarski and Kantor: 238.

2016 *Pyrgulabakuana* (Kolesnikov, 1947). – Vinarski and Kantor: 236–237.

**Status.** Accepted Pontocaspian species.

**Type locality.** In fossil limestone of Dagestan, Russia.

**Distribution.** Middle and southern Caspian Sea (Logvinenko and Starobogatov 1969). This species was mentioned from depths between 200 and 500 m in the South Caspian Basin of Azerbaijan (Mirzoev and Alekperov 2017).

**Taxonomic notes.** Although there is little doubt about the validity of this species, its true identity and possible synonyms are unclear. Eichwald’s (1838) description clearly indicates a slender shell with median keel. His type material is unfortunately unknown. The high number of keeled species complicates an evaluation what is the “true” *T.dimidiata* and what are synonyms. We tentatively consider *Turricaspiabakuana* Kolesnikov, 1947 a junior synonym of this species, based on its slender shell with median keel matching Eichwald’s description as well as the prevailing concept of *T.dimidiata* (compare Kantor and Sysoev 2006). More data are required to support this view.

**Conservation status.** Not assessed.

***Turricaspiaeburnea* (Logvinenko & Starobogatov, 1969)**

*1969 Pyrgula (Laevicaspia) eburnea Logvinenko & Starobogatov: 370, fig. 365(1).

2016 *Turricaspiaeburnea* (Logvinenko & Starobogatov, 1968). – Vinarski and Kantor: 247.

**Status.** Pontocaspian species, identity uncertain.

**Type locality.** Eastern part of the southern Caspian Sea.

**Distribution.** South Caspian Basin. This species was mentioned from depths between 200 and 500 m in the South Caspian Basin of Azerbaijan (Mirzoev and Alekperov 2017).

**Taxonomic notes.** The identity of this species is unclear. Its shell resembles *T.lyrata* (Dybowski & Grochmalicki, 1915) in terms of general shape and the large, flat protoconch; it differs from that species in the large size. The type material has been very recently found in the collection of ZIN and awaits further study. Until then, we refrain from a final decision on the species’ status, but we have severe doubt that *Pyrgulaeburnea* is a distinct species.

**Conservation status.** Not assessed.

***Turricaspiaelegantula* (Clessin & Dybowski in Dybowski, 1887)**

*1887 *Micromelaniaelegantula* Clessin & Dybowski in Dybowski: 33.

1888 [*Micromelania*] *elegantula* n. sp. – Dybowski: 78, pl. 1, fig. 7a–c.

2016 *Turricaspiaelegantula* (Clessin & W. Dybowski in W. Dybowski, 1888). – Vinarski and Kantor: 247–248.

**Status.** Pontocaspian species, identity uncertain.

**Type locality.** Caspian Sea (no details).

**Distribution.** Endemic to the Caspian Sea. This species was mentioned from depths between 200 and 300 m in the South Caspian Basin of Azerbaijan (Mirzoev and Alekperov 2017).

**Taxonomic notes.** There is considerable confusion about the identity of this species. Dybowski (1887) described and illustrated a very slender shell with a distinct whorl profile showing a straight-sided upper half and a convex lower half. In contrast, the illustrations in Logvinenko and Starobogatov (1969) suggest a similarly slender yet distorted shell with near almost sided whorls and expanded aperture. A restudy of the type material of *T.elegantula* show close similarities to *T.spica*. It differs from that species in the more slender outline and flattened whorls.

**Conservation status.** Not assessed.

***Turricaspiaeulimellula* (Dybowski & Grochmalicki, 1915)**

*1915 Micromelania (Turricaspia) eulimellula Dybowski & Grochmalicki: 123–125, pl. 3, fig. 27a, b.

2006 *Pyrgulaeulimellula* (B. Dybowski & J. Grochmalicki, 1915). – Kantor and Sysoev: 99–100, pl. 46, fig. L.

2016 *Pyrgulaeulimellula* (B. Dybowski & Grochmalicki, 1915). – Vinarski and Kantor: 238–239.

**Status.** Accepted Pontocaspian species.

**Type locality.** Caspian Sea (no details).

**Distribution.** Middle Caspian Sea Basin (Logvinenko and Starobogatov 1969). This species was mentioned from depths between 200 and 400 m in the South Caspian Basin of Azerbaijan (Mirzoev and Alekperov 2017).

**Taxonomic notes.** The nearly straight-sided, strongly attached whorls easily distinguish this species from most other *Turricaspia* species. Only *Turricaspiagrimmi* (Clessin & Dybowski in Dybowski, 1887) has a similar whorl arrangement, but its shell is slightly wider and the whorls are weakly stepped and bear a thin subsutural band.

**Conservation status.** Not assessed.

***Turricaspiafedorovi* (Logvinenko & Starobogatov, 1969)**

*1969 Pyrgula (Turricaspia) fedorovi Logvinenko & Starobogatov: 362, fig. 360(2).

2016 *Pyrgulafedorovi* Logvinenko & Starobogatov, 1968. – Vinarski and Kantor: 239.

**Status.** Pontocaspian species, identity uncertain.

**Type locality.** Western part of the middle Caspian Sea, 80 m.

**Distribution.** Middle and South Caspian Basin. This species was mentioned from depths between 200 and 400 m in the South Caspian Basin of Azerbaijan (Mirzoev and Alekperov 2017).

**Taxonomic notes.** The slender elongate shell with whorls slowly increasing in height distinguishes this species from its congeners. However, a proper assessment of the species’ status requires investigation. The whereabouts of the type material is unknown and no other records of this species are known, so we are not able to verify the status of this species.

**Conservation status.** Not assessed.

***Turricaspiagrimmi* (Clessin & Dybowski in Dybowski, 1887)**

*1887 *MicromelaniaGrimmi* Clessin & Dybowski in Dybowski: 27–29.

1888 [*Micromelania*] *Grimmi* n. sp. – Dybowski: 78, pl. 1, fig. 2a–c.

2006 *Pyrgulagrimmi* (Clessin & W. Dybowski in W. Dybowski, 1888). – Kantor and Sysoev: 100, pl. 46, fig. L.

2016 *Pyrgulagrimmi* (Clessin & W. Dybowski in W. Dybowski, 1888). – Vinarski and Kantor: 239.

**Status.** Accepted Pontocaspian species.

**Type locality.** Caspian Sea (no details).

**Distribution.** Southern Caspian Sea Basin (Logvinenko and Starobogatov 1969). This species was mentioned from depths between 200 and 300 m in the South Caspian Basin of Azerbaijan (Mirzoev and Alekperov 2017).

**Taxonomic notes.** The peculiar morphology with straight-sided, weakly stepped whorls with a thin subsutural band is unique among Caspian Pyrgulinae. See above for a comparison with *T.eulimellula*.

**Conservation status.** Data Deficient (Vinarski 2011i).

***Turricaspialyrata* (Dybowski & Grochmalicki, 1915)**

*1915 Micromelania (Turricaspia) spicavar.lyrata Dybowski & Grochmalicki: 117, pl. 2, fig. 18.

2006 *Pyrgulalirata* [sic] (B. Dybowski & J. Grochmalicki, 1915). – Kantor and Sysoev: 101, pl. 46, fig. E.

2016 *Pyrgulalirata* [sic] (B. Dybowski & Grochmalicki, 1915). – Vinarski and Kantor: 240.

2018 *Turricaspialyrata* (B. Dybowski & Grochmalicki, 1915). – Neubauer et al.: 77–79, fig. 12A–K [and synonyms therein].

**Status.** Accepted Pontocaspian species.

**Type locality.** Caspian Sea (no details).

**Distribution.** Endemic to the Caspian Sea (after Logvinenko and Starobogatov 1969); it occurs in the western part of the middle and southern Caspian Sea basins, but these authors used a slightly different concept of the species. This species was mentioned from depths between 200 and 300 m in the South Caspian Basin of Azerbaijan (Mirzoev and Alekperov 2017, who reported the species as *Turricaspialirata*).

**Taxonomic notes.** See Neubauer et al. (2018) for a detailed discussion of the species and its synonyms.

**Conservation status.** Not assessed.

***Turricaspiamarisnigri* Starobogatov in Alexenko & Starobogatov, 1987**

*1987 *Turricaspialiratamarisnigri* Starobogatov in Alexenko & Starobogatov: 33.

**Status.** Pontocaspian species, identity uncertain.

**Type locality.** “Meotida” station 24, sample 229, near the coast of Crimea, in phaseoline silt (Holocene).

**Distribution.** Type locality only.

**Taxonomic notes.** The species can be distinguished based on its extremely slender shell with whorls slowly increasing in size. Still, clarification of its identity as well as its generic classification requires investigation of additional material.

**Conservation status.** So far only known from Holocene deposits of the type locality; species might be extinct. Within Holocene deposits in the Black Sea small amounts of reworked Late Pleistocene “Neoeuxinian” faunas are found (FW, pers. obs.), and therefore the stratigraphic origin of such Pontocaspian species is uncertain.

***Turricaspiameneghiniana* (Issel, 1865)**

*1865 *Bythinia Meneghiniana* Issel: 21, pl. 1, figs 12, 13.

1902a *Micromelaniasubulata* Westerlund: 47.

?1969 *Pyrgulacaspia* (Eichw). – Logvinenko and Starobogatov: 369–370, fig. 364(1) [**non***Rissoacaspia* Eichwald, 1838].

non 1987 *T.*[*urricaspia*] *meneghiniana meneghiniana* (Iss.). – Alexenko and Starobogatov: 35, fig. 8.

2006 *Turricaspiameneghiniana* (Issel, 1865). – Kantor and Sysoev: 109, pl. 49, fig. E.

2016 *Turricaspiameneghiniana* (Issel, 1865). – Vinarski and Kantor: 248.

2018 *Turricaspiameneghiniana* (Issel, 1865). – Neubauer et al.: 79–81, fig. 13A–K [and synonyms therein].

**Status.** Accepted Pontocaspian species.

**Type locality.** Baku, Azerbaijan; (sub?)fossil.

**Distribution.** Middle and southern Caspian Sea (Logvinenko and Starobogatov 1969).

**Taxonomic notes.** The species was recently discussed in detail by Neubauer et al. (2018), who also discussed previous misidentifications.

**Conservation status.** Not assessed.

***Turricaspianossovi* Kolesnikov, 1947**

*1947 *Turricaspianossovi* Kolesnikov: 108, 111.

2006 *Pyrgulanossovi* (Kolesnikov, 1947). – Kantor and Sysoev: 101, pl. 45, fig. G.

2016 *Pyrgulanossovi* (Kolesnikov, 1947). – Vinarski and Kantor: 241.

**Status.** Accepted Pontocaspian species.

**Type locality.** Caspian Sea (no details).

**Distribution.** Southern Caspian Sea (Logvinenko and Starobogatov 1969). This species was mentioned from depths between 200 and 500 m in the South Caspian Basin of Azerbaijan (Mirzoev and Alekperov 2017).

**Taxonomic notes.** The very slender shape and the characteristic, highly convex whorls that slowly and regularly increase in height distinguish the species from most congeners. *Pyrgulavinogradovi* Logvinenko & Starobogatov, 1969 and *P.astrachanica* Pirogov, 1971, which show very similar traits, might be junior synonyms. A more in-depth study is required to solve their statuses.

**Conservation status.** Data Deficient (Vinarski 2011l).

? ***Turricaspiaobventicia* (Anistratenko in Anistratenko & Prisyazhniuk, 1992)**

*1992 Caspia (Clathrocaspia) obventicia Anistratenko in Anistratenko & Prisyazhniuk: 19–20, fig. 2b.

**Status.** Uncertain Pontocaspian species.

**Type locality.** Well 37 near Kiliya, Izmail district, Odessa region, Ukraine (from Holocene sediments).

**Distribution.** Type locality only.

**Taxonomic notes.** This species was originally attributed to the genus *Caspia* due to its small shell. A study of the holotype of this species, specifically its protoconch characteristics, suggest placement in the genus *Turricaspia*. Further studies are required to assure its validity.

**Remarks.** The species is known only from the holotype. The occurrence of *Turricaspia* in the Black Sea Basin is unusual, as almost all other pyrguline Black Sea species are assigned to the genus *Laevicaspia* (but see remark at *T.spica* for another unusual occurrence).

**Conservation status.** So far only known from Holocene deposits of the type locality; species might be extinct.

? ***Turricaspiapseudobacuana* (Logvinenko & Starobogatov, 1969)**

*1969 Pyrgula (Eurycaspia) pseudobacuana Logvinenko & Starobogatov: 358, fig. 358(16).

2016 *Pyrgulapseudobacuana* Logvinenko & Starobogatov, 1968. – Vinarski and Kantor: 241.

**Status.** Pontocaspian species, probably junior synonym.

**Type locality.** Southern Caspian Sea, 50–80 m.

**Distribution.** South Caspian Basin. This species was mentioned from depths between 200 and 300 m in the South Caspian Basin of Azerbaijan (Mirzoev and Alekperov 2017).

**Taxonomic notes.** The slender shell with a keel near the lower suture is reminiscent of *T.basalis* (Dybowski & Grochmalicki, 1915). The short description and poor drawing precluded the verification of its status. The type material has been very recently detected in the collection of ZIN and awaits further study.

**Conservation status.** Not assessed.

? ***Turricaspiapseudodimidiata* (Dybowski & Grochmalicki, 1915)**

*1915 Micromelania (Turricaspia) pseudodimidiata Dybowski & Grochmalicki: 126–128, pl. 3, fig. 32a, b.

?1969 Pyrgula (Eurycaspia) pseudodimidiata (Dyb. et Gr.). – Logvinenko and Starobogatov: 357, fig. 358(15).

?2006 *Pyrgulapseudodimidiata* (B. Dybowski & Grochmalicki, 1915). – Kantor and Sysoev: 102, pl. 47, fig. G.

2016 *Pyrgulapseudodimidiata* (B. Dybowski & Grochmalicki, 1915). – Vinarski and Kantor: 241.

**Status.** Pontocaspian species, identity uncertain.

**Type locality.** Caspian Sea (no details).

**Distribution.** Southern Caspian Sea (Logvinenko and Starobogatov 1969). This species was mentioned from depths between 200 and 300 m in the South Caspian Basin of Azerbaijan (Mirzoev and Alekperov 2017).

**Taxonomic notes.** The identity of this species is uncertain. Dybowski and Grochmalicki (1915) describe and illustrate a shell with eight convex whorls bearing a weak, hardly protruding, irregular shaped keel near the lower suture. According to these authors, the keel varies considerably between a thin thread, a blunt bulge, or a weak thickening at the suture. In contrast, the drawings provided by Logvinenko and Starobogatov (1969) and reproduced by Kantor and Sysoev (2006) suggest a shell with straight-sided whorls and a distinct keel. Inspection of the type material is required to clarify the status of this species.

**Conservation status.** Not assessed.

***Turricaspiapseudospica* (Logvinenko & Starobogatov, 1969)**

*1969 Pyrgula (Oxypyrgula) pseudospica Logvinenko & Starobogatov: 366, fig. 363(1).

2016 *Pyrgulapseudospica* Logvinenko & Starobogatov, 1968. – Vinarski and Kantor: 241–242.

**Status.** Pontocaspian species, identity uncertain.

**Type locality.** Middle and southern Caspian Sea, 15–75 m.

**Distribution.** Type locality only.

**Taxonomic notes.** The identity of this species is unclear. Judging from the drawing by Logvinenko and Starobogatov (1969), showing a small slender shell with ca. 6.5 convex whorls, the species might be based on a juvenile specimen. Moreover, it could be a junior synonym of the similarly shaped *T.spica* (Eichwald, 1855).

**Conservation status.** Not assessed.

***Turricaspiapulla* (Dybowski & Grochmalicki, 1915)**

*1915 Micromelania (Turricaspia) caspiavar.pulla Dybowski & Grochmalicki: 111, pl. 1, fig. 6a.

1969 *Pyrgula* [(*Turricaspia*)] *pulla* (Dyb. et Gr.). – Logvinenko and Starobogatov: 361–

362, fig. 360(8).

2006 *Pyrgulapulla* (B. Dybowski & Grochmalicki, 1915). – Kantor and Sysoev: 102, pl. 46, fig. C.

2016 *Pyrgulapulla* (B. Dybowski & Grochmalicki, 1915). – Vinarski and Kantor: 242.

2018 *Turricaspiapulla* (B. Dybowski & Grochmalicki, 1915). – Neubauer et al.: 81–82, fig. 14A–J.

**Status.** Accepted Pontocaspian species.

**Type locality.** Caspian Sea (no details).

**Distribution.** Endemic to the Caspian Sea, reported from the middle and southern Caspian Sea basins (Logvinenko and Starobogatov 1969). This species was mentioned from depths between 200 and 300 m in the South Caspian Basin of Azerbaijan (Mirzoev and Alekperov 2017).

**Taxonomic notes.** The species can be easily distinguished from other *Turricaspia* species based on its relatively broad shell, the low-convex whorls, and its small size (Neubauer et al. 2018).

**Conservation status.** Data Deficient (Vinarski 2011m).

***Turricaspiapullula* (Dybowski & Grochmalicki, 1915)**

*1915 Micromelania (Turricaspia) caspiavar.pullula Dybowski & Grochmalicki: 111–112, pl. 1, fig. 7.

1969 *Pyrgula* [(*Turricaspia*)] *pullula* (Dyb. et Gr.). – Logvinenko and Starobogatov:

366–367, fig. 363(3).

2006 *Turricaspiapullula* (B. Dybowski & Grochmalicki, 1915). – Kantor and Sysoev: 109, pl. 50, fig. B.

2016 *Turricaspiapullula* (B. Dybowski & Grochmalicki, 1915). – Vinarski and Kantor: 249.

2018 *Turricaspiapullula* (B. Dybowski & Grochmalicki, 1915). – Neubauer et al.: 82–84, fig. 14K–L.

**Status.** Accepted Pontocaspian species.

**Type locality.** Caspian Sea (no details).

**Distribution.** Endemic to the Caspian Sea, reported from the western part of the middle Caspian Sea (Logvinenko and Starobogatov 1969).

**Taxonomic notes.** The very characteristic tripartite whorl profile allows an easy identification and discrimination from other Pontocaspian Pyrgulinae (Neubauer et al. 2018).

**Conservation status.** Data Deficient (Vinarski 2011s).

***Turricaspiarudis* (Logvinenko & Starobogatov, 1969)**

*1969 Pyrgula (Turricaspia) rudis Logvinenko & Starobogatov: 362, fig. 360(5).

2016 *Pyrgularudis* Logvinenko & Starobogatov, 1968. – Vinarski and Kantor: 242.

**Status.** Pontocaspian species, identity uncertain.

**Type locality.** Middle and southern Caspian Sea, 50–100 m.

**Distribution.** Type locality only.

**Taxonomic notes.** The status of this species is unclear. The drawing provided by Logvinenko and Starobogatov (1969) shows strong similarities to *T.grimmi* in terms of the nearly straight-sided whorls and the large aperture. Since the whereabouts of the type material is unknown, we refrain from a final conclusion on the potential synonymy.

**Conservation status.** Data Deficient (Vinarski 2011n).

***Turricaspiasajenkovae* (Logvinenko & Starobogatov, 1969)**

*1969 Pyrgula (Turricaspia) sajenkovae Logvinenko & Starobogatov: 361, fig. 360(4).

2016 *Turricaspiasajenkovae* (Logvinenko & Starobogatov, 1968). – Vinarski and Kantor: 249–250.

**Status.** Pontocaspian species, identity uncertain.

**Type locality.** Middle Caspian Sea.

**Distribution.** Type locality only.

**Taxonomic notes.** The available drawing of this species suggests a very slender shell with highly convex whorls bearing a subsutural band. The type material has not been found, and the identity of this species remains unclear.

**Conservation status.** Data Deficient (Vinarski 2011t).

***Turricaspiasimilis* (Logvinenko & Starobogatov, 1969)**

*1969 Pyrgula (Caspiella) similis Logvinenko & Starobogatov: 375, fig. 366(11).

2016 *Pyrgulasimilis* Logvinenko & Starobogatov, 1968. – Vinarski and Kantor: 243.

**Status.** Pontocaspian species, identity uncertain.

**Type locality.** Eastern part of the middle Caspian Sea, 20–50 m.

**Distribution.** Middle and southern Caspian Basin. This species was mentioned from depths between 200 and 300 m in the South Caspian Basin of Azerbaijan (Mirzoev and Alekperov 2017).

**Taxonomic notes.** Judging from the drawing in Logvinenko and Starobogatov (1969), presenting a small slender shell with ca. 5.5 highly convex whorls, the species might be based on a juvenile specimen. It might be a junior synonym of the similarly shaped *T.meneghiniana* (Issel, 1865). Without investigating the type material, which has not been found in the ZIN collection, the identity of this species remains unclear.

**Conservation status.** Not assessed.

***Turricaspiasimplex* (Logvinenko & Starobogatov, 1969)**

*1969 Pyrgula (Oxypyrgula) simplex Logvinenko & Starobogatov: 367–368, fig. 363(4).

2016 *Pyrgulasimplex* Logvinenko & Starobogatov, 1968. – Vinarski and Kantor: 243.

**Status.** Pontocaspian species, identity uncertain.

**Type locality.** Middle Caspian Sea, 40–120 m.

**Distribution.** Middle and southern Caspian Sea. This species was mentioned from depths between 200 and 900 m in the South Caspian Basin of Azerbaijan (Mirzoev and Alekperov 2017).

**Taxonomic notes.** As for the previous species, it is highly uncertain whether this taxon is a distinct species. It might also be based on a juvenile and could be a synonym of an earlier described species, perhaps *T.pulla* or *T.lyrata*.

**Conservation status.** Not assessed.

***Turricaspiaspasskii* (Logvinenko & Starobogatov, 1969)**

*1969 Pyrgula (Turricaspia) spasskii Logvinenko & Starobogatov: 361, fig. 360(7).

2016 *Turricaspiaspasskii* (Logvinenko & Starobogatov, 1968). – Vinarski and Kantor: 250.

**Status.** Accepted Pontocaspian species.

**Type locality.** Western part of the middle Caspian Sea.

**Distribution.** Middle and southern Caspian Sea. This species was mentioned from depths between 200 and 300 m in the South Caspian Basin of Azerbaijan (Mirzoev and Alekperov 2017).

**Taxonomic notes.** The fast growing whorls terminating in a large body whorl with expanded aperture are characteristic for this species and facilitate discrimination from other *Turricaspia* species.

**Conservation status.** Data Deficient (Vinarski 2011u).

***Turricaspiaspica* (Eichwald, 1855)**

*1855 *Paludinaspica* Eichwald: 303–304, pl. 10, figs 8, 9.

?1992 *Turricaspiaspica* (Eichw.). – Anistratenko and Prisyazhniuk: 18, fig. 2d.

2006 *Turricaspiaspica* (Eichwald, 1855). – Kantor and Sysoev: 110, pl. 49, fig. F.

2009 Turricaspiacf.spica (Eichwald, 1855). – Filippov and Riedel: 70, 72, 74, 76, fig. 4e, f.

2016 *Turricaspiaspica* (Eichwald, 1855). – Vinarski and Kantor: 250.

**Status.** Accepted Pontocaspian species.

**Type locality.** Ostrov Chechen’ (island in NW Caspian Sea), Dagestan, Russia.

**Distribution.** Endemic to the Caspian Sea. Occurred also in the Aral Sea during the Holocene (Filippov and Riedel 2009) but now extinct there. It has been reported from the Holocene of Danube Delta (Anistratenko and Prisyazhniuk 1992) (see below).

**Taxonomic notes.** As the oldest described species presently attributed to *Turricaspia*, the validity of this species is without doubt. Its identity, however, is poorly known, given the limited information and poor drawing provided by Eichwald (1855), as well as the largely diverging concepts applied by later authors (see Neubauer et al. 2018 for a detailed discussion of the matter). We have a geographic record (Anistratenko and Prisyazhniuk 1992) that is outside the Caspian–Aral distribution range of this genus. Comparison of the Danube material with Caspian specimens suggests the identification might be correct, yet further detail study is required to assess whether the Danube record might actucally not be an unusual form of *Laevicaspialincta*.

**Conservation status.** Not assessed.

***Turricaspiaturricula* (Clessin & Dybowski in Dybowski, 1887)**

*1887 *Micromelaniaturricula* Clessin & Dybowski in Dybowski: 34.

1888 [*Micromelania*] *turricula* n. sp. – Dybowski: 78, pl. 1, fig. 3a–c.

2006 *Turricaspiaturricula* (Clessin & W. Dybowski in W. Dybowski, 1888). – Kantor and Sysoev: 111, pl. 49, fig. I.

2016 *Turricaspiaturricula* (Clessin & W. Dybowski in W. Dybowski, 1888). – Vinarski and Kantor: 244.

**Status.** Accepted Pontocaspian species.

**Type locality.** Caspian Sea (no details).

**Distribution.** Middle and southern Caspian Sea. This species was mentioned from depths between 200 and 500 m in the South Caspian Basin of Azerbaijan (Mirzoev and Alekperov 2017).

**Taxonomic notes.** The species is characterised by a slender conical shell with weakly convex whorls with weak subsutural swelling and a slightly inflated body whorl with large aperture.

**Conservation status.** Not assessed.

***Turricaspiauralensis* (Logvinenko & Starobogatov, 1969)**

*1969 Pyrgula (Turricaspia) uralensis Logvinenko & Starobogatov: 359, fig. 360(1).

2016 *Pyrgulauralensis* Logvinenko & Starobogatov, 1968. – Vinarski and Kantor: 244.

**Status.** Pontocaspian species, identity uncertain.

**Type locality.** Eastern part of the northern Caspian Sea.

**Distribution.** Type locality only.

**Taxonomic notes.**Logvinenko and Starobogatov (1969) illustrated a comparably small shell with eight highly convex whorls, large body whorl, and large aperture. Reliable assessment of the species’ status requires investigation of the type material, which has only been discovered in ZIN in June 2018 and awaits further study.

**Conservation status.** Not assessed.

***Turricaspiavinogradovi* (Logvinenko & Starobogatov, 1969)**

*1969 Pyrgula (Oxypyrgula) vinogradovi Logvinenko & Starobogatov: 368, fig. 363(9).

?1971 *Pyrgulaastrachanica* Pirogov: 249–251, fig. 1.

?2006 *Turricaspiaastrachanica* (Pirogov, 1971). – Kantor and Sysoev: 105, pl. 48, fig. B.

2006 *Turricaspiavinogradovi* (Logvinenko & Starobogatov, 1968). – Kantor and Sysoev: 111, pl. 50, fig. C.

2016 *Turricaspiavinogradovi* (Logvinenko & Starobogatov, 1968). – Vinarski and Kantor: 251.

**Status.** Pontocaspian species, identity uncertain.

**Type locality.** Northern Caspian Sea.

**Distribution.** Northern Caspian Sea and Volga Delta (Logvinenko and Starobogatov 1969).

**Taxonomic notes.** The species as illustrated by Logvinenko and Starobogatov (1969) is based on a slender shell with highly convex whorls. The same traits are also typical for *Pyrgulaastrachanica*; in fact, the type of *T.vinogradovi* could be a juvenile of that species. Moreover, both of them might be synonyms of *Turricaspianossovi* Kolesnikov, 1947. Since a part of the type material of the species involved is lacking and some of the taxa are based on incomplete or presumably juvenile specimens, the identities of *Pyrgulaastrachanica* and *Turricaspiavinogradovi* remain unresolved.

**Conservation status.***Turricaspiavinogradovi* has not been assessed by the IUCN, *T.astrachanica* is marked as “Data Deficient” (Vinarski 2011q).

#### Hydrobiidae incertae sedis

***Abeskunusbrusinianus* (Clessin & Dybowski in Dybowski, 1887)**

*1887 *Zagrabica Brusiniana* Clessin & Dybowski in Dybowski: 52–53.

1888 *Zagrabica Brusiniana* n. sp. – Dybowski: 79, pl. 2, fig. 7.

2006 *Pseudamnicolabrusiniana* (Clessin & W. Dybowski in W. Dybowski, 1888). – Kantor and Sysoev: 114, pl. 51, fig. J.

2016 *Pseudamnicolabrusiniana* (Clessin & W. Dybowski in W. Dybowski, 1888). – Vinarski and Kantor: 222.

2018 *Abeskunusbrusinianus* (Clessin & W. Dybowski in W. Dybowski, 1887). – Neubauer et al.: 87–88, fig. 16A–I.

**Status.** Accepted Pontocaspian species.

**Type locality.** Caspian Sea (no details).

**Distribution.** Middle and southern Caspian Sea (Logvinenko and Starobogatov 1969, Parr et al. 2007). Mirzoev and Alekperov (2017) mention *Pseudamnicolabrusinianus* from depths between 200 and 400 m in the South Caspian Basin of Azerbaijan but we are not entirely certain whether these records might include other *Abeskunus* species as well.

**Taxonomic notes.** For a detailed description and discussion, see Neubauer et al. (2018).

**Conservation status.** Least Concern (Vinarski 2011c).

***Abeskunusdepressispira* (Logvinenko & Starobogatov, 1969)**

*1969 Pseudamnicola (Abeskunus) depressispira Logvinenko & Starobogatov: 381, fig. 367(14).

2016 *Pseudamnicoladepressispira* Logvinenko & Starobogatov, 1968. – Vinarski and Kantor: 222–223.

**Status.** Accepted Pontocaspian species.

**Type locality.** Western part of the southern Caspian Sea, northward of Kuraginsky Kamen’ [= Kür Daşı] Island (approximately 39°01'05"N, 49°20'02"E), 81 m water depth.

**Distribution.** In addition to the type locality, specimens have been found in Holocene material retrieved near the Kura Delta, a few kilometres north of the type locality.

**Taxonomic notes.** Current investigations on recently collected Holocene material from the south-western Caspian Sea confirm that this species belongs to the genus *Abeskunus*. The finely ribbed, low trochiform shell facilitates distinction from its congeners. The species epithet is based on the Latin noun *spira*, spire, and is to be considered a noun in apposition (ICZN 1999, Art. 31.2.1.).

**Conservation status.** Data Deficient (Vinarski 2011d).

***Abeskunusexiguus* (Eichwald, 1838)**

°1837 *Lithoclypus* [sic] *Caspius* m. – Krynicki: 58 (nomen nudum).

*1838 *Paludinaexigua* Eichwald: 152–153.

1863 *Bithiniasphaerion* Mousson: 409–410.

1874 *Lithoglyphus*? *Caspius* Krynicki. – Martens: 80.

1877 *Lithoglyphuscaspius* Grimm: 82–84, pl. 9, fig. 8.

1977 Pseudamnicola (Abeskunus) brusinianamichelae Tadjalli-Pour: 108, pl. 2, fig. 9.

2016 *Pseudamnicolaexigua* (Eichwald, 1838). – Vinarski and Kantor: 223.

2016 *Pseudamnicolasphaerion* (Mousson, 1863). – Vinarski and Kantor: 223.

**Status.** Accepted Pontocaspian species.

**Type locality.** In fossil (likely Pleistocene) limestone of Dagestan, Russia.

**Distribution.** Western Caspian Sea, known from northern and southern parts. Records from the eastern Caspian Sea by Logvinenko and Starobogatov (1969) could not be confirmed.

**Taxonomic notes.** An in-depth study of the literature suggests that the names *Paludinaexigua*, *Bithiniasphaerion* syn. n., and *Lithoglyphuscaspius* all refer to the same species. The name *Lithoglyphuscaspius* was made available by Martens (1874) by referring to the description and illustration of Eichwald’s species, rendering *L.caspius* a junior objective synonym of *Abeskunusexiguus*. All three taxa share the globular shape, short spire, and inflated last whorl. The subspecies *Pseudamnicolabrusinianamichelae* syn. n. from Iranian coasts of the Caspian Sea closely resembles *A.exiguus* and is herein considered a synonym as well. *Abeskunusexiguus* differs from *A.brusinianus* in the highly globular shell with small spire. A revision of the species is currently being prepared.

**Conservation status.** Not assessed.

***Andrusoviaandrusovi* Starobogatov, 2000**

*2000 *Andrusoviaandrusovi* Starobogatov: 39–41, fig. 1B.

2016 *Andrusoviaandrusovi* Starobogatov, 2000. – Vinarski and Kantor: 214.

**Status.** Pontocaspian species, identity uncertain.

**Type locality.** Eastern part of the South Caspian Sea (39°05'N, 52°35'E).

**Distribution.** Middle and southern Caspian Sea (Starobogatov 2000).

**Taxonomic notes.** The species is very similar to the type species of *Andrusovia*, *A.dybowskii*, regarding the low spire. Investigation of the type material is required to clarify whether both taxa are distinct.

**Remarks.** Only recently, paratypes of this species were detected at the Zoological Museum of Moscow University. A study of the taxonomy of *Andrusovia* is currently under way.

**Conservation status.** Not assessed.

***Andrusoviabrusinai* Starobogatov, 2000**

*2000 *Andrusoviabrusinai* Starobogatov: 41, fig. 1C.

2016 *Andrusoviabrusinai* Starobogatov, 2000. – Vinarski and Kantor: 214.

2018 *Andrusoviabrusinai* Starobogatov, 2000. – Neubauer et al.: 54–56, fig. 6F–K, M–N.

**Status.** Pontocaspian species, identity uncertain.

**Type locality.** Eastern part of the middle Caspian Sea (42°42.5'N, 51°32.5'E), at 80 m water depth.

**Distribution.** Northern, middle, and southern Caspian Sea (Starobogatov 2000, Neubauer et al. 2018).

**Taxonomic notes.** The species was recently described in detail by Neubauer et al. (2018). The species was distinguished from *A.dybowskii* and *A.andrusovi* by the higher spire, but this is a variable character. Currently, the taxonomy of *Andrusovia* species is the subject of further study.

**Remarks.**Starobogatov (2000) mentioned that the type material is housed in the ZIN collection, but we were unable to find the holotype and it is presumed lost. Only recently, paratypes of this species were detected at the Zoological Museum of Moscow University and are currently being studied.

**Conservation status.** Not assessed.

***Andrusoviadybowskii* Brusina in Westerlund, 1902b**

*1902b *AndrusoviaDybowskii* Westerlund: 133.

? 2000 *Andrusoviadybowskii* Brusina in Westerlund, 1903. – Starobogatov: 39, fig. 1A.

2016 *Andrusoviadybowskii* Brusina in Westerlund, 1903. – Vinarski and Kantor: 214.

**Status.** Accepted Pontocaspian species.

**Type locality.** Caspian Sea (no details).

**Distribution.** Middle and southern Caspian Sea (Starobogatov 2000).

**Taxonomic notes.** Apparently, Brusina considered both the more conical and flatter shells (“conoidea vel discoidea”) to belong to a single species. Starobogatov (2000) in turn referred only the flat type to as *Andrusoviadybowskii* and considered the conical ones to belong to separate species (*A.brusinai* and *A.marina*). The recently rediscovered type material represents the conico-globular type and is currently subject of study by V. Anistratenko and collaegues.

**Conservation status.** Not assessed.

***Andrusoviamarina* (Logvinenko & Starobogatov, 1969)**

*1969 Horatia (Caspiohoratia) marina Logvinenko & Starobogatov: 382, fig. 367(18).

2000 *Andrusoviamarina* (Logvinenko & Starobogatov, 1969). – Starobogatov: 41–42, fig. 1D.

2016 *Andrusoviamarina* (Logvinenko & Starobogatov, 1968). – Vinarski and Kantor: 214–215.

**Status.** Pontocaspian species, identity uncertain.

**Type locality.** Northern slope of the middle Caspian Sea Basin, 43°32.5'N, 49°17.5'E, 60 m water depth.

**Distribution.** Middle and southern Caspian Sea (Starobogatov 2000). This species was mentioned from depths between 200 and 400 m in the South Caspian Basin of Azerbaijan (Mirzoev and Alekperov 2017, who reported the species as *Horatiamarina*).

**Taxonomic notes.** According to Neubauer et al. (2018), this species might be a senior synonym of *A.brusinai* Starobogatov, 2000. Inspection of recently discovered type material appears to support that view, but more in-depth studies are required to evaluate the status of this species.

**Remarks.** The holotype is not traced and presumed lost. Only recently, paratypes of this species were detected at the Zoological Museum of Moscow University and are currently being studied.

**Conservation status.** Not assessed.

#### Family Lithoglyphidae Tryon, 1866

***Lithoglyphusnaticoides* (Pfeiffer, 1828)**

*1828 *Paludinanaticoides* Pfeiffer: 45–46, pl. 8, figs 1, 2, 4.

2012 *Lithoglyphusnaticoides* (Pfeiffer, 1828). – Welter-Schultes: 41, unnumbered text figures.

2016 *Lithoglyphusnaticoides* (C. Pfeiffer, 1828). – Vinarski and Kantor: 253.

**Status.** Accepted native species.

**Type locality.** In the Danube at Vienna, Austria, and at Pesth (today part of Budapest), Hungary.

**Distribution.** Originally only in rivers entering the Black Sea, in the Danube up to Regensburg (Germany). After 1800, also introduced to Elbe and Rhine regions by artificial canals; after 1900 in France (Welter-Schultes 2012). Very common in the Volga Delta (Vinarski et al. 2018).

**Conservation status.** Least Concern (Van Damme 2011b).

#### Family Tateidae Thiele, 1925

***Potamopyrgusantipodarum* (Gray, 1843)**

*1843 *Amnicolaantipodarum* Gray: 241.

1951 *Potamopyrgusjenkinsi* E. A. Smith 1889. – Grossu: 693–695, fig. 1a–d.

1966 *P.*[*yrgula*] (*Trachycaspia*?) *grossui* Golikov and Starobogatov: 359.

1991 *Potamopyrguspolistchuki* Anistratenko: 75, fig. 1(2).

1995 *Potamopyrgusalexenkoae* Anistratenko in Anistratenko and Stadnichenko: 92–93, fig. 69.

2012 *Potamopyrgusantipodarum* (Gray, 1843). – Welter-Schultes: 40, unnumbered text figures.

**Status.** Accepted species, invasive.

**Type locality.** New Zealand (no details).

**Distribution.** Originally from New Zealand, probably introduced in 1859 to England, in 1872 to Tasmania, in 1895 to mainland Australia, in ca. 1900 to European mainland (Ponder 1988), and in 1987 to North America (Zaranko et al. 1997).

**Taxonomic notes.** The two Black Sea species *P.polistchuki* syn. n. and *P.alexenkoae* syn. n. are here considered as junior synonyms of *P.antipodarum*, differing only very weakly in outline. Vinarski and Kantor (2016) listed *Pyrgula* (*Trachycaspia*?) *grossui* syn. n. Golikov & Starobogatov in the synonymy of *T.dimidiata* (Eichwald, 1838). Golikov and Starobogatov (1966) introduced this species as new name for the supposedly misidentified *Potamopyrgusjenkinsi* sensu Grossu (1951) from Razim Lake in Romania. The shell they later illustrated (Golikov and Starobogatov 1972) indeed shows similarities with *T.dimidiata*. The shell illustrated in Grossu (1951), however, is completely different and shows a keeled form of *P.antipodarum*.

**Conservation status.** Least Concern (Van Damme 2013).

#### Family Planorbidae Rafinesque, 1815

***Gyrauluseichwaldi* (Clessin & Dybowski in Dybowski, 1887)**

°1876 *Pl.*[*anorbis*] *Eichwaldi*. – Grimm: 157 (nomen nudum).

*1887 *PlanorbisEichwaldi* Clessin & Dybowski in Dybowski: 49–52.

1888 *PlanorbisEichwaldi* Grimm. – Dybowski: 79, pl. 2, fig. 11a–c, pl. 3, fig. 10a–c.

?1966b Anisus (Andrusowia) [sic] *eichwaldiinfundibularis* Logvinenko and Starobogatov: 1472, fig. 4.

?1977 *Anisusdjalali* Tadjalli-Pour: 109, pl. 2, fig. 10.

2016 Gyraulus (Gyraulus) eichwaldi (Grimm in W. Dybowski, 1888). – Vinarski and Kantor, 2016: 378.

**Status.** Accepted Pontocaspian species.

**Type locality.** Caspian Sea (no details).

**Distribution.** Middle and southern Caspian Sea (Logvinenko and Starobogatov 1969). This species was mentionedfrom depths between 200 and 900 m in the South Caspian Basin of Azerbaijan (Mirzoev and Alekperov 2017, who reported the species as *Anisuseichwaldi*).

**Taxonomic notes.** The species is characterised by a relatively large, asymmetrical shell. *Anisuseichwaldiinfundibularis* is probably a morphotype of *G.eichwaldi*. We are uncertain about the status of *Anisusdjalali* Tadjalli-Pour, 1977 as the description is very brief and the photographs are not very clear. It may be within the range of the morphological variability of *G.eichwaldi*.

**Conservation status.** Not assessed.

***Gyraulusdybowskii* (Kolesnikov, 1947)**

*1947 Planorbiseichwaldivar.dybowskii Kolesnikov: 109, 112, fig. in tab. 1.

1966b Anisus (Andrusowia) [sic] *kolesnikovi* Logvinenko and Starobogatov: 1473, fig. 5.

1966b Anisus (Andrusowia) [sic] *kolesnikovi sublittoralis* Logvinenko and Starobogatov: 1472–1473, fig. 6.

2016 Gyraulus (Gyraulus) kolesnikovi (Logvinenko & Starobogatov, 1966). – Vinarski and Kantor, 2016: 379.

**Status.** Pontocaspian species, identity uncertain.

**Type locality.** Caspian Sea, 40°37'N, 50°52'E, 115 m.

**Distribution.** Middle and southern Caspian Sea (Logvinenko and Starobogatov 1969). This species was mentioned from depths between 200 and 300 m in the South Caspian Basin of Azerbaijan (Mirzoev and Alekperov 2017, who reported the species as *Anisuscolesnikovi* [sic]).

**Taxonomic notes.**Logvinenko and Starobogatov (1966b) considered this species and *Andrusoviadybowskii* Brusina in Westerlund, 1902b to belong in the same genus, Anisus (Andrusovia), rendering *P. dybowskii* Kolesnikov, 1947 a junior homonym. Therefore, they introduced *A.kolesnikovi* as replacement name. Since both taxa do clearly not belong to the same genus or even the same family, the replacement name is to be discarded.

The species resembles *G.eichwaldi* regarding the general habitus; it differs in the more pronounced angle at the transition between whorl flank and apical plane. A revision is required to investigate if the Caspian *Gyraulus* species are distinct species or morphotypes of *G.eichwaldi*. The generic placement follows Vinarski and Kantor (2016). Note that those authors listed the earlier described *P. eichwaldi dybowskii* Kolesnikov, 1947 as a synonym of *G.kolesnikovi*.

**Conservation status.** Least Concern (for *Anisuskolesnikovi*; Vinarski 2011a).

***Gyraulussulcatus* (Logvinenko & Starobogatov, 1966, non Hilgendorf, 1867)**

*1966b Anisus (Andrusowia) [sic] *sulcatus* Logvinenko & Starobogatov: 1474, fig. 7.

2016 Gyraulus (Gyraulus) sulcatus (Logvinenko & Starobogatov, 1966). – Vinarski and Kantor, 2016: 382.

**Status.** Pontocaspian species, identity uncertain, name invalid.

**Type locality.** Caspian Sea, 42°45'N, 48°29'E, 79 m.

**Distribution.** Middle Caspian Sea (Logvinenko and Starobogatov 1969).

**Taxonomic notes.** The species in its present combination as *Gyraulussulcatus* (following Vinarski and Kantor 2016) is invalid as it is a secondary homonym of the Miocene *Gyraulussulcatus* (Hilgendorf, 1867). We refrain here from introducing a replacement name as the species’ status is uncertain. It resembles *G.eichwaldi* and *G.kolesnikovi* in outline shape and differs only in the more pronounced angle between whorl flank and apical plane and the shallow furrow on the apical side. An in-depth revision is required to clarify if *Gyraulussulcatus* is a distinct species or a mere morphotype of *G.eichwaldi* (Clessin & Dybowski in Dybowski, 1887).

**Conservation status.** Not assessed.

## Discussion and conclusions

The annotated check-list presented here is a first attempt to assess the species diversity of the Pontocaspian molluscs by experts working in different countries and fields (neontology, palaeontology, biogeography, phylogenetics). Hitherto, progress has been limited by a number of factors: (1) fresh material for genetic studies is available only for few nominal species, and (2) the type series of many species are lost or at least have not yet been found. This concerns not only the species described by Eichwald or Grimm in the 19^th^ century; the type specimens of many species established by Starobogatov and his co-workers in the 1960–2000s could not be traced in ZIN (Kantor and Sysoev 2006, Vinarski and Kantor 2016). Furthermore, progress has been limited by (3) a lack of representative shell samples to undertake quantitative statistical analyses of conchological variation, and (4) insufficient ecological and distribution data for many of the species.

Three species that have been reported from the Pontocaspian region are not included in this list. The bithyniid gastropod *Alocinmacaspica* (Westerlund, 1902) has been described from the east side of the Caspian Sea (Beriozkina et al. 1995 indicated this record is probably from the vicinity of Krasnovodsk, Turkmenistan). However, Starobogatov et al. (2004) argued the species lives in waterbodies of Bol’shoy Balkhan (Turkmenistan) and probably not in the Caspian Sea itself (Vinarski et al. 2013, Vinarski and Kantor 2016). Furthermore, two *Pseudamnicola* species have been described from Lake Razim in Romania (*P.leontina* Grossu, 1986 and *P.razelmiana* Grossu, 1986) that is prime Pontocaspian habitat. Like bithyniids, *Pseudamnicola* has not been reported as a Pontocaspian group elsewhere, and probably they are freshwater species that live in the surrounding streams or in springs. For now, we have excluded these species from the Pontocaspian species list.

This list contains 55 accepted and a further 44 uncertain endemic Pontocaspian mollusc species (Table 2), here defined as species that are considered to be endemic for at least one of the Pontocaspian basins. There are 14 native and three immigrant species (at least in one of the Pontocaspian basins), even though some species may be native or endemic in one of the basins and have become invasive in another of the Pontocaspian Basins. All species that have an uncertain status belong to the Pontocaspian category. The Caspian Sea Basin has the highest number of accepted endemic Pontocaspian species (48) but also poses the greatest taxonomic challenges, with a further 37 species whose status are unclear.

**Table 2.** Pontocaspian mollusc species list. Abbreviations: Status: A – accepted, U – uncertain. Basins: AS – Aral Sea, BSB – Black Sea Basin, CSB – Caspian Sea Basin. Species are E – endemic, EX – extinct, IM – immigrant, IN – invasive, N – native (definitions in Table 1); *species encountered alive during the PRIDE program expeditions by participants; ^†^very fresh material of species encountered, but not living specimens.

Species	Status	BSB	CSB	AS
*Mytilasterminimus* (Poli, 1795)*	A	N	IN	IM/EX
*Adacnalaeviuscula* (Eichwald, 1829)	A	?	E	
*Adacnafragilis* Milaschewitsch, 1908	U	E		
*Adacnaminima* Ostroumov, 1907	A		E	E/EX?
*Adacnaminimaostroumovi* (Logvinenko & Starobogatov, 1967)	U		E	
*Adacnavitrea* (Eichwald, 1829)	A	E	E	E/EX?
*Adacnavitreaglabra* Ostroumov, 1905	U	E	E	
*Adacnavitreabergi* (Starobogatov, 1974)	U			E/EX?
*Cerastodermaglaucum* (Bruguière, 1789) s.l.*	A	N	IN	IN?
*Cerastoderma* sp. A [non *C.rhomboides* (Lamarck, 1819)]*	A	N	IN	IN?
*Didacnabaeri* (Grimm, 1877)	A		E	
*Didacnabarbotdemarnii* (Grimm, 1877)*	A		E	
*Didacnaeichwaldi* (Krynicki, 1837)	A		E	
*Didacnalongipes* (Grimm, 1877)*	A		E	
*Didacnaparallela* Bogachev, 1932	A		E	
*Didacnapraetrigonoides* Nalivkin & Anisimov, 1914	A		E/EX	
*Didacnaprofundicola* Logvinenko & Starobogatov, 1966^†^	A		E	
*Didacnaprotracta* (Eichwald, 1841)	A		E	
*Didacnapyramidata* (Grimm, 1877)	A		E	
*Didacnatrigonoides* (Pallas, 1771)*	A		E	
*Hypanisplicata* (Eichwald, 1829)	A	E	E	
*Monodacnaacuticosta* (Logvinenko & Starobogatov, 1967)	A		E	
*Monodacnaalbida* (Logvinenko & Starobogatov, 1967)	A		E	
*Monodacnacaspia* (Eichwald, 1829)	A		E	?
*Monodacnacolorata* (Eichwald, 1829)*	A	E	IM	
*Monodacnafilatovae* (Logvinenko & Starobogatov, 1967)	U		E	
*Monodacnaknipowitschi* (Logvinenko & Starobogatov, 1966)	U		E	
*Monodacnapolymorpha* (Logvinenko & Starobogatov, 1967)	U		E	
*Monodacnasemipellucida* (Logvinenko & Starobogatov, 1967)	A		E	
*Abrasegmentum* (Récluz, 1843)*	A	N	IN	IN
*Corbiculafluminalis* (Müller, 1774)	A		N/IN	
*Dreissenabugensis* Andrussov, 1897^†^	A	E/IN	IN	
*Dreissenacaspia* Eichwald, 1855	A		E/EX	E/EX
*Dreissenaelata* Andrusov, 1897	U		E/EX	
*Dreissenagrimmi* (Andrusov, 1890)*	A		E	
*Dreissenapolymorpha* (Pallas, 1771) s.l.*	A	N	N	N
*Mytilopsisleucophaeata* (Conrad, 1831)*	A	IN	IN	
*Theodoxusdanubialis* (Pfeiffer, 1828)*	A	N		
*Theodoxusfluviatilis* (Linnaeus, 1758)	A	N		
*Theodoxuspallasi* Lindholm, 1924*	A	N	N	N/EX?
*Theodoxusschultzii* (Grimm, 1877)*	U		E	
*Theodoxusvelox* V. Anistratenko in O. Anistratenko et al., 1999	A	N		
*Eupaludestrinastagnorum* (Gmelin, 1791)	A	N/IM	N/IM	
*Caspiabaerii* Clessin & Dybowski in Dybowski, 1887	A	E?	E	
?*Caspiavalkanovi* (Golikov & Starobogatov, 1966)	U	E		
*Clathrocaspiabrotzkajae* (Starobogatov in Anistratenko & Prisyazhniuk, 1992)	A	?E	E	
*Clathrocaspiagmelinii* (Clessin & Dybowski in Dybowski, 1887)	A		E	
*Clathrocaspiaisseli* (Logvinenko & Starobogatov, 1969)	U		E	
*Clathrocaspiaknipowitschii* (Makarov, 1938)	A	E		
*Clathrocaspialogvinenkoi* (Golikov & Starobogatov, 1966)	A	E		
*Clathrocaspiamilae* Boeters, Glöer & Georgiev, 2015	U	E		
*Clathrocaspiapallasii* (Clessin & Dybowski in Dybowski, 1887)	A		E	
*Ulskiabehningi* (Logvinenko & Starobogatov, 1969)	U		E	
?*Ulskiaderzhavini* (Logvinenko & Starobogatov, 1969)	U		E	
*Ulskiaulskii* (Clessin & Dybowski in Dybowski, 1887)	A		E	
*Ecrobiagrimmi* (Clessin in Dybowski, 1887)*	A		N	N
*Ecrobiamaritima* (Milaschewitsch, 1916)*	A	N		
*Ecrobiaventrosa* (Montagu, 1803)	A	IM		
*Clessiniolavariabilis* (Eichwald, 1838)	A	E	E	
*Laevicaspiaabichi* (Logvinenko & Starobogatov, 1969)	A		E	
*Laevicaspiacaspia* (Eichwald, 1838)	A		E	
*Laevicaspiacincta* (Abich, 1859)	A		E	
*Laevicaspiaconus* (Eichwald, 1838)	A		E	
?*Laevicaspiaebersini* (Logvinenko & Starobogatov, 1969)	U		E	
?*Laevicaspiaismailensis* (Golikov & Starobogatov, 1966)	A	E		
*Laevicaspiakolesnikoviana* (Logvinenko & Starobogatov in Golikov & Starobogatov, 1966)	A		E	
*Laevicaspiakowalewskii* (Clessin & Dybowski in Dybowski, 1887)	A		E	
*Laevicaspialencoranica* (Logvinenko & Starobogatov, 1969)	U		E	
*Laevicaspialincta* (Milaschewitsch, 1908)	A	E		
?*Laevicaspiamarginata* (Westerlund, 1902)	U		E	
*Laevicaspiasieversii* (Clessin in Dybowski, 1887)	U		E	
?*Turricaspiaaenigma* (Logvinenko & Starobogatov, 1969)	U		E	
*Turricaspiaandrussowi* (Dybowski & Grochmalicki, 1915)	A		E	
?*Turricaspiabasalis* (Dybowski & Grochmalicki, 1915)	U		E	
?*Turricaspiabogatscheviana* (Logvinenko & Starobogatov, 1969)	U		E	
*Turricaspiachersonica* Alexenko & Starobogatov, 1987	U	E		
*Turricaspiacolumna* (Logvinenko & Starobogatov, 1969)	U		E	
*Turricaspiaconcinna* (Logvinenko & Starobogatov, 1969)	U		E	
*Turricaspiadagestanica* (Logvinenko & Starobogatov, 1969)	U		E	
*Turricaspiadimidiata* (Eichwald, 1838)	A		E	
*Turricaspiaeburnea* (Logvinenko & Starobogatov, 1969)	U		E	
*Turricaspiaelegantula* (Clessin & Dybowski in Dybowski, 1887)	U		E	
*Turricaspiaeulimellula* (Dybowski & Grochmalicki, 1915)	A		E	
*Turricaspiafedorovi* (Logvinenko & Starobogatov, 1969)	U		E	
*Turricaspiagrimmi* (Clessin & Dybowski in Dybowski, 1887)	A		E	
*Turricaspialyrata* (Dybowski & Grochmalicki, 1915)	A		E	
*Turricaspiamarisnigri* Starobogatov in Alexenko & Starobogatov, 1987	U	E/EX?		
*Turricaspiameneghiniana* (Issel, 1865)	A		E	
*Turricaspianossovi* Kolesnikov, 1947	A		E	
?*Turricaspiaobventicia* (Anistratenko in Anistratenko & Prisyazhniuk, 1992)	U	E		
?*Turricaspiapseudobacuana* (Logvinenko & Starobogatov, 1969)	U		E	
?*Turricaspiapseudodimidiata* (Dybowski & Grochmalicki, 1915)	U		E	
*Turricaspiapseudospica* (Logvinenko & Starobogatov, 1969)	U		E	
*Turricaspiapulla* (Dybowski & Grochmalicki, 1915)	A		E	
*Turricaspiapullula* (Dybowski & Grochmalicki, 1915)	A		E	
*Turricaspiarudis* (Logvinenko & Starobogatov, 1969)	U		E	
*Turricaspiasajenkovae* (Logvinenko & Starobogatov, 1969)	U		E	
*Turricaspiasimilis* (Logvinenko & Starobogatov, 1969)	U		E	
*Turricaspiasimplex* (Logvinenko & Starobogatov, 1969)	U		E	
*Turricaspiaspasskii* (Logvinenko & Starobogatov, 1969)	A		E	
*Turricaspiaspica* (Eichwald, 1855)	A	?E	E	?E
*Turricaspiaturricula* (Clessin & Dybowski in Dybowski, 1887)	A		E	
*Turricaspiauralensis* (Logvinenko & Starobogatov, 1969)	U		E	
*Turricaspiavinogradovi* (Logvinenko & Starobogatov, 1969)	U		E	
*Abeskunusbrusinianus* (Clessin & Dybowski in Dybowski, 1887)	A		E	
*Abeskunusdepressispira* (Logvinenko & Starobogatov, 1969)	A		E	
*Abeskunusexiguus* (Eichwald, 1838)	A		E	
*Andrusoviaandrusovi* Starobogatov, 2000	U		E	
*Andrusoviabrusinai* Starobogatov, 2000	U		E	
*Andrusoviadybowskii* Brusina in Westerlund, 1902	A		E	
*Andrusoviamarina* (Logvinenko & Starobogatov, 1969)	U		E	
*Lithoglyphusnaticoides* (Pfeiffer, 1828)*	A	N	IM?	
*Potamopyrgusantipodarum* (Gray, 1843)*	A	IM		
*Gyrauluseichwaldi* (Clessin & Dybowski in Dybowski, 1887)^†^	A		E	
*Gyraulusdybowskii* (Kolesnikov, 1947)	U		E	
*Gyraulussulcatus* (Logvinenko & Starobogatov, 1966)	U		E	

The species richness estimate reflects the current shift of molluscan systematics from morphology-based to integrated studies, with increasing contributions of molecular and statistical species delineation approaches (Vinarski 2018). It has recently been shown that many nominal taxa of fresh- and brackish-water snails and mussels described on the basis of their shell characters (the Pontocaspian molluscs rarely were described on the base of anatomical studies) lack a genetic support (with few exceptions such as e.g., Popa et al. 2012, Stepien et al. 2013) and thus do not represent evolutionary meaningful units. On the other hand, cryptic speciation is known within many taxa of molluscs in long-lived lakes (Albrecht et al. 2006), and the Pontocaspian biota may include some previously unrecognised species. Thus, we consider our check-list rather as a starting point for further integrated research, not a definitive and fixed inventorisation of the Pontocaspian molluscs.

Anyone who reads this list or works such as Logvinenko and Starobogatov (1969) or Vinarski and Kantor (2016) may think that the Caspian Sea still maintains its unique and species-rich mollusc fauna. However, the actual state of affairs is problematic as many species thought to be endemic to this large saline lake have not been found since their description, and recent attempts to obtain fresh material for genetic studies mostly failed. Clearly, the conservation status of Pontocaspian species is insufficiently known. With our working list we aim to assist in the necessary follow-up conservation assessments.

Most taxonomic difficulties were encountered for the bivalve genera *Monodacna* and *Dreissena* and the Pyrgulinae gastropods (especially genera *Turricaspia* and *Laevicaspia*). Furthermore, there is an urgent need to assess whether representatives of species complexes in the three main Pontocaspian basins (Aral Sea, Caspian Sea, Black Sea) concern separate species as several of these regional populations are in immediate danger of extinction or already extinct (for example with the disappearance of the Aral Sea). Combined methodological efforts will enable us to estimate the extent and characterise the nature of Pontocaspian faunal turnover, and this species list is a first attempt in the required uniform taxonomic base.
